# Database derived from an electronic medical record-based surveillance network of US emergency department patients with acute respiratory illness

**DOI:** 10.1186/s12911-023-02310-4

**Published:** 2023-10-17

**Authors:** Jeffrey A. Kline, Brian Reed, Alex Frost, Naomi Alanis, Meylakh Barshay, Andrew Melzer, James W. Galbraith, Alicia Budd, Amber Winn, Eugene Pun, Carlos A. Camargo

**Affiliations:** 1 Department of Emergency Medicine, Wayne State University, Detroit, MI USA; 2 Department of Emergency Medicine, Wayne State University, Detroit, MI USA; 3 Newton Massachusetts, Newton, MA USA; 4 Department of Emergency Medicine, John Peter Smith Hospital, Ft. Worth, TX USA; 5 Department of Emergency Medicine, George Washington University School of Medicine, Washington, DC USA; 6 Department of Emergency Medicine, George Washington University School of Medicine, Washington, DC USA; 7 Department of Emergency Medicine, University of Mississippi Medical Center, Jackson, MS USA; 8 Influenza Division, National Center for Immunization and Respiratory Diseases, Centers for Disease Control and Prevention, Atlanta, USA; 9 Coronavirus and Other Respiratory Viruses Division, National Center for Immunization and Respiratory Diseases, Centers for Disease Control and Prevention, Atlanta, USA; 10 General Dynamics Contractor to the Influenza Division, National Center for Immunization and Respiratory Diseases, Centers for Disease Control and Prevention, Atlanta, USA; 11 Present Address: Department of Emergency Medicine, Massachusetts General Hospital, Harvard Medical School, Boston, MA USA

**Keywords:** Epidemiology, Emergency medicine, SARS-CoV-2, Electronic medical records

## Abstract

**Background:**

For surveillance of episodic illness, the emergency department (ED) represents one of the largest interfaces for generalizable data about segments of the US public experiencing a need for unscheduled care. This protocol manuscript describes the development and operation of a national network linking symptom, clinical, laboratory and disposition data that provides a public database dedicated to the surveillance of acute respiratory infections (ARIs) in EDs.

**Methods:**

The Respiratory Virus Laboratory Emergency Department Network Surveillance (RESP-LENS) network includes 26 academic investigators, from 24 sites, with 91 hospitals, and the Centers for Disease Control and Prevention (CDC) to survey viral infections. All data originate from electronic medical records (EMRs) accessed by structured query language (SQL) coding. Each Tuesday, data are imported into the standard data form for ARI visits that occurred the prior week (termed the index file); outcomes at 30 days and ED volume are also recorded. Up to 325 data fields can be populated for each case. Data are transferred from sites into an encrypted Google Cloud Platform, then programmatically checked for compliance, parsed, and aggregated into a central database housed on a second cloud platform prior to transfer to CDC.

**Results:**

As of August, 2023, the network has reported data on over 870,000 ARI cases selected from approximately 5.2 million ED encounters. Post-contracting challenges to network execution have included local shifts in testing policies and platforms, delays in ICD-10 coding to detect ARI cases, and site-level personnel turnover. The network is addressing these challenges and is poised to begin streaming weekly data for dissemination.

**Conclusions:**

The RESP-LENS network provides a weekly updated database that is a public health resource to survey the epidemiology, viral causes, and outcomes of ED patients with acute respiratory infections.

**Supplementary Information:**

The online version contains supplementary material available at 10.1186/s12911-023-02310-4.

## Background

The emergency department (ED) serves as a valuable site for surveillance because of its large patient volume and diverse representation of illness. At least 120 million Americans visit an ED each year, and about 5–10% of these patients present with an acute respiratory illness (ARI) [1]. This report describes the methodology for the creation and operation of a national network of EDs providing linked symptom, clinical, laboratory and disposition data weekly for patients presenting to the ED with ARI, aggregated in a single database. This work began as a retrospective registry known as RECOVER (REgistry of suspected COVID-19 in EmeRgency care) in March 2020 with the explicit purpose of tracking clinical features and outcomes of patients tested for SARS-CoV-2 [2]. That registry led to the development of the present network and database, funded by contract 75D30121C11813, and named the Respiratory Virus Laboratory Emergency Department Network Surveillance (RESP-LENS). A primary and unique approach of this surveillance network–that distinguishes it from existing networks–is the use of weekly extractions from electronic medical records to provide nearly real-time insight into the dynamics of viral transmission and severity of illness in patients seeking unscheduled care. Unlike other networks, RESP-LENS will link patient level data from the ED visit, such as vital signs, with results of laboratory testing, and then with 30 day outcome. These data are especially relevant to tracking the severity of illness associated with outbreaks of new SARS-CoV-2 variants [3, 4]. and comparing the relative impact of SARS-CoV-2, influenza and RSV circulation.

## Construction and content

### Overview

**Fig. 1:**
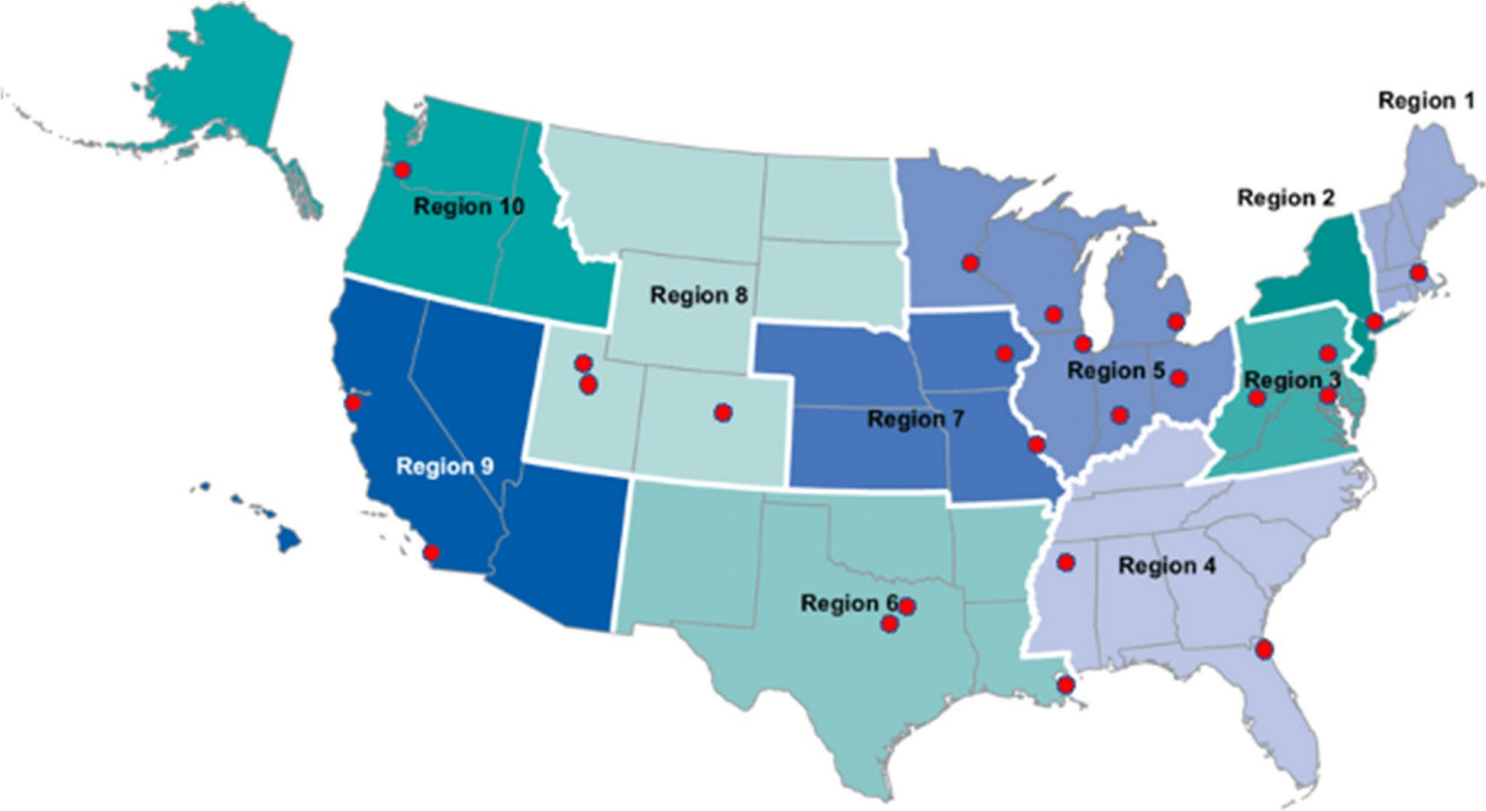
Locations of the 24 sites participating in RECOVER-CDC. Many sites have more than one hospital

The database input is entirely dependent upon the RESP-LENS network, which represents a consortium of 24 emergency medicine-based clinician investigators from 21 US states. These investigators lead 24 sites (network hubs) that report data from 109 hospitals (network nodes), including 5 children’s hospitals. The sites and hospitals represent all 10 Department of Health and Human Services regions (Fig. 1 and Supplemental Table 1). All sites were recruited from the larger RECOVER network [2]. In terms of representativeness, the network includes hospitals that serve inner city urban populations (e.g., Detroit MI, San Francisco CA and New York City), many metropolitan and suburban regions, rural areas (e.g. Morganton, WV and Iowa City, IA) as well as cities at higher altitude (Colorado Springs CO and Salt Lake City, UT). Five hospitals are children’s hospitals. The network lacks any representation from the island regions such as Hawaii.

For RESP-LENS, the definition of an ARI comes from one or more of the 130 ICD-10 diagnosis codes associated with the ED visit (Supplemental Table 2). These ICD-10 codes were derived from a consensus effort by investigators and CDC personnel and remain comprehensive of symptoms of illness caused by SARS-CoV-2 variants and other respiratory viruses [5]. All ED visits are identified electronically, using either Epic (Verona, WI) or Cerner (Kansas City, MO) electronic medical record systems. Because many patients have more than one visit within a week, hereafter we use the nomenclature “case” to define any given patient visit that satisfied the ICD definition of an ARI.

**Fig. 2:**
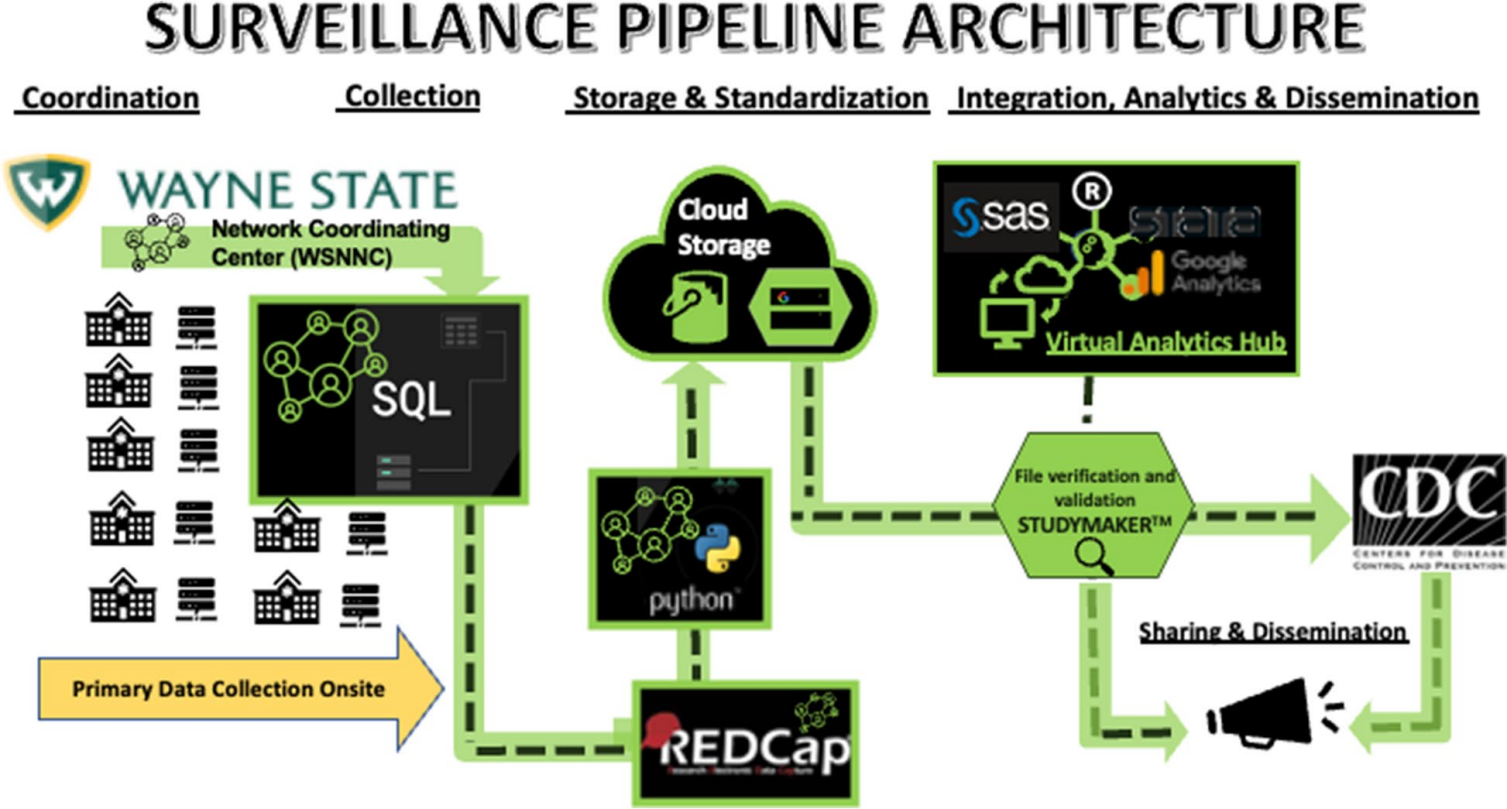
Architecture of the RECOVER-CDC surveillance network

All case data are acquired from the electronic health record and auto-populate a database via structured query language (SQL), using either the Clarity or Electronic Data Warehouse (*HealtheEDW*^SM^, Kansas City, MO) data analytical systems [6]. Fig. 2 shows the overview of the data transmission workflow which begins at each site and is then transmitted to a centralized Google Cloud (Mountain View, CA) storage owned by the coordinating site, analyzed, parsed, and aggregated in the Study Maker (Newton, MA) cloud platform, and then ultimately transferred to CDC. This process runs weekly: sites transmit data on Mondays or Tuesdays to Google Cloud and the coordinating site transmits to CDC on Wednesdays.

### Network management

Wayne State University in Detroit, MI functions as the overall network coordinating center, which encompasses the roles of data coordinating center, site recruitment, contract management, creation of the REDCap data forms and their content, site payment, and repository of network documents including the manual of operations, data management plan, and study protocol. The network communicates via listserv email, quarterly all-site meetings via teleconference, and using Slack Teams™ (San Francisco, CA). The kick-off meeting occurred in September of 2021 via teleconference. All-site meetings always included representatives from CDC. After the program launched, sites proceeded to share information, including SQL code, toward creating the methods required for weekly data abstraction from the EMRs of the 24 sites. Each site requires, at minimum, a principal investigator (MD or PhD), and an informatics/coding professional.

### Roles and responsibilities

The original protocol that described the role of Wayne State as the network and data coordinator was written by the Contact PI at Wayne State and submitted in response to a publicly posted funding opportunity announcement from CDC. With input from CDC, the Contact PI oversaw the recruitment of sites to represent all 10 DHHS regions, their subcontracting, and the content of the REDCap case report form. The Contact PI sets the agenda and leads the quarterly all-site meetings, answers questions about field definitions, and handles unexpected problems. The Contact PI wrote a data use agreement that was used by most sites and attends weekly organizational meetings with CDC. This meeting is open to all network participants. The Wayne State network data manager was responsible for setting up the secure Google Cloud platform and encrypted, password-protected folders for each site and maintaining a living field definition document that explains the standardization of codes to signify missing data (999 and -999 for string or numeric data respectively), unexpected characters (e.g., > or <), and out of range data (e.g., age > 120 years). The Wayne State project manager oversees communications, including scheduling, audiovisual recordings of meetings, maintenance of contact information and delegation of authority logs for all sites, storing documents and other files, and maintaining a manual of operations.

At each site, the site PI initiates and takes responsibility for executing the terms of the subcontract, data use agreement, and other regulatory aspects (i.e., IRB), attending all site meetings and addressing problems such as updating the SQL code in response to local changes in viral platform testing/ordering, and interruptions in data transfer because of local personnel changes. Additionally, Wayne State has subcontracted with StudyMaker to provide technical support and software systems for data ingestion, verification, export, and transmission, as well as to maintain a contemporaneous aggregated dataset mirrored back to Wayne State and delivered via exports to CDC.

Site analysts are responsible for writing and updating the SQL code and setting up the data transfer process to the Wayne Google Cloud using an API. The site analyst assists with communications with the Wayne State project manager and ensures timely data transfer on a weekly basis.

Representatives of CDC have been involved in all stages from the outset of creating the data collection forms, site selection, all-site meetings, and weekly feedback to sites. CDC is responsible for general oversight and execution of the contract supporting this work and managing the project to ensure data needed to meet CDC objectives are obtained weekly. To accomplish this, CDC staff were involved in developing all aspects of the data collection protocol and forms, provided input on site selection as needed, analyzed weekly data and provided feedback to the coordinating site regarding data anomalies and questions to ensure accurate data interpretation.

Overall governance is run by an executive committee consisting of the contact PI, four members of CDC, and the two consultants (a StudyMaker representative and the outside epidemiologist). Data sharing is overseen by the data use committee, comprising all 24 site PIs.

### Regulatory issues and registrations

The protocol has been reviewed by all site institutional review boards and deemed non-human subjects research, exempted from review or approved with expedited review. Except for patient ages and dates of service, the data transmitted for RESP-LENS contains no other protected health information. The data management plan has been registered using the DMP tool and is publicly available (https://dmptool.org/plans/70148). The University of Colorado was instrumental in this project's development of the first SQL script for use with Epic; unfortunately, the State of Colorado had a constitutional statement that barred the University of Colorado from remaining in the network because of risks associated with Federal Acquisition Regulation (FAR) clauses in the Prime agreement.

### Case data acquisition

**Fig. 3:**
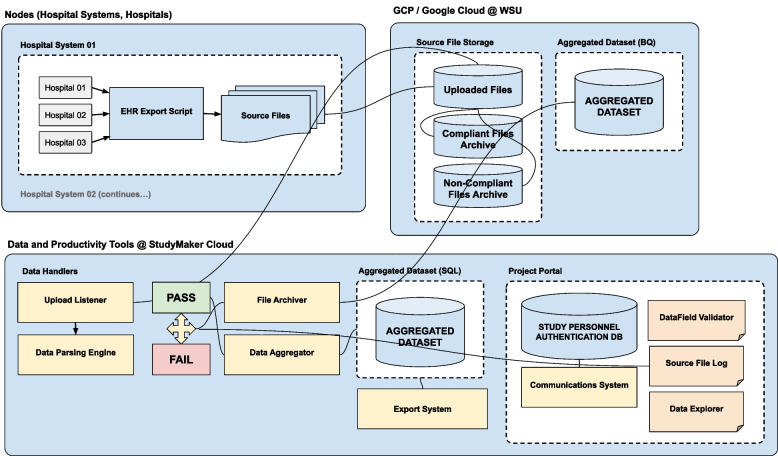
Schematic of the flow and processing of data

Figure 3 shows a schematic overview of the network and flow of data. REDCap was utilized to develop a shareable mechanism for data standardization and validation (in the form of CSV project data dictionaries or XML project files). It also provided a facile approach for distributing project updates as revisions were made to the data collection process.

The REDCAP database was constructed in three data collection forms: (1) the 173 field “ndex visit” form that captures initial ED visit data (clinical, laboratory, disposition and demographic) from ARI cases, (2) the “30-day” form that includes 136 fields for results of new or repeated laboratory and diagnostic testing, as well as, admission and survival outcome for ARI cases; and (3) the third file is the “ED volume” form that includes overall ED visit data, including age and admission status, for both ARI and non-ARI cases. Data are captured at the encounter level rather than patient level with each qualifying visit logged consecutively, without regard to prior entry while still maintaining patient-level identifiers. Sites transmit encrypted or hashed identifiers (e.g., Financial record number[FIN] and the Medical record number [MRN]) rather than the original identifiers to protect confidentiality while aligning index and 30-day data and facilitating the tracking of individual subjects over time. The index and 30-day files include 325 fields shown in Supplemental File 3. For sex, race and ethnicity fields, all sites used the designations provided by the patient. The ED volume file shows the age and admission status of each ED patient seen on an individual row.

Each site’s local copy of the network-wide REDCap projects provided them with the means to perform automated data validation on their EMR query results. All reports generated by site EMR queries (possibly subject to post-processing) can be tested for compliance via a manual or API directed import into the site’s local REDCap projects. If query files load successfully into the site’s unaltered copy of the network-wide REDCap projects, they will load successfully into the corresponding projects housed in Wayne State’s REDCap environment.

### Case data submission

On a weekly basis, site analysts upload the “index visit,” “30-day,” and “ED volume” files from their local REDCAP database to a Google Cloud Storage (GCS) environment maintained by Wayne State. Each site has one or more designated individuals who have been granted access to a secure, specific bucket in the GCS environment. Each site’s bucket contains three primary folders for each file type reported from sites – “index visit,” “30-day”, and “ED volume.” The three subfolders each contain respective subfolders for storing ingestion log information and archiving site files that have been successfully loaded or rejected according to whether they meet the data specification.

### Data processing

In the initial phase of the study, REDCap was utilized as the study data repository and transport mechanism until the volume of data generated exceeded the REDCAP server's operational capacity. At the onset of the study, all files uploaded from sites to the GCS buckets were processed using a series of Python utilities. The first was a validation stage in which an attempt was made to load incoming site files to a test project via an “Import Records” REDCap API method. The result of this attempted load was logged and recorded in the GCS bucket subfolder for each respective site. Files loaded successfully were transferred into a queue for the second processing stage, namely the transfer to CDC. In cases when files failed to load, the log was reviewed to determine the cause, or causes, of failure, and these details were communicated back to sites. Load failure could be caused in all file types (Index, 30-Day, or ED Volume) by data related errors, incorrect field names, additional fields not listed in the network-wide project specification, data type inconsistencies (i.e. transmission of text data for numeric fields), and data coding inconsistencies (i.e. using improper numeric coding for categorical responses). Thirty-Day Follow-up files could also fail validation due to the presence of unmatched records. By definition, 30-day follow-up data must provide information for an existing index record. If a record in a 30-day follow-up file does not have a matching index record in the cumulative database, it either represents a new, previously unidentified case, or it contains a malformed record ID. These newly discovered index cases often occur because of delay in charting by emergency physicians, especially on visits made near the end of the week, followed by delays from coding and billing services. By protocol, the index files are “trued up” on at least a quarterly basis to include these visits that were newly discovered at 30-day follow-up.

As more sites executed their contracts and completed their SQL coding, the weekly volume of data began to escalate. The volume of data eventually began to affect the performance of the required API methods for import and export. This resulted in frequent 503 HTTP errors where the resource demand generated by the API exceeded the REDCap server’s operational capacity. Even after adjustments to the REDCap server memory allocation and timeout settings, resource related errors occurred too frequently for REDCap to remain a tenable solution for data storage and transfer.

To overcome bottlenecks and extend data management capabilities, the central functions of REDCap at Wayne State (data validation, data aggregation, and data export generation) were replaced with a dedicated data parser and connected data handling and data management tools in the StudyMaker cloud platform. Accordingly, Parquet was deployed to parse data and prepare data exports in convenient formats to improve data transmission to the CDC central data lake (software and systems maintained by StudyMaker, Newton, MA). This new architecture retains full compatibility with REDCap, so that no changes were required by the Sites, and so that Sites can continue to use REDCap locally for data validation and assessment.

### Assessing data fidelity

**Fig. 4:**
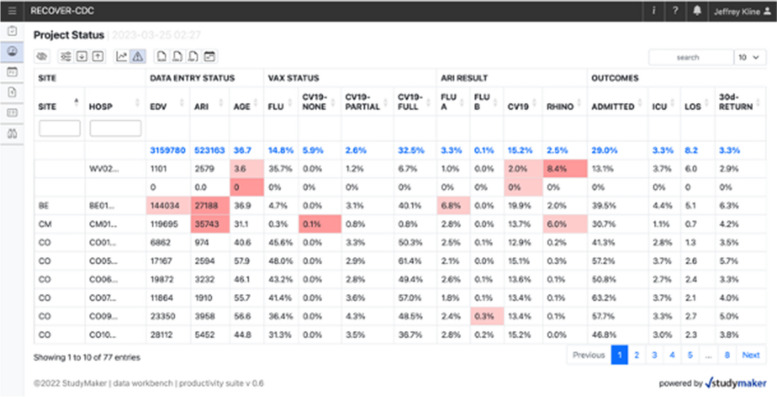
Representation of the weekly counter verification and validation dashboard, with visualization of outliers

For this report, fidelity refers to the consistency and accuracy of data upload from each site. To monitor the successful transmission and accuracy of data submitted by sites, files uploaded from sites are automatically detected, tested for compliance and presence of mandatory data. In the event of a missing or late file, the system automatically generates an email to the site PI and lead coder. We produced a dashboard-based monitoring system that demonstrates all inbound data from networks, including the time of upload and the percentage of fields reported as missing or empty (that latter is also known as a null value). Fields could be missing or empty for least three reasons: (1) Truly missing: data that should have been present in the EMR but were absent either from clerical omission or the data were unknown (an example of the latter, could be for an unconscious patient with no prior medical record) (2) Empty or null: failure to find the data because of SQL coding error, or (3) Pseudomissing: if the patient only had an index visit, and no other contact within 30 days, most of the questions at 30 days had no data to fill.These dashboards are provided in a secure study portal for participants to (1) track their own data quality performance, (2) track trends and key endpoints from live data submitted to the network, and (3) explore the full dataset for research purposes through a graphical interface. Figure 4 shows an export of files loaded from week 48 of 2022 in the “Weekly counter” dashboard.

### Production

All files that successfully completed the validation phase were placed in the queue for transfer to the production environment. The production phase again utilized the REDCap “Import Records” API method to import validated records into the production environment. The production environment served as the cumulative data repository, and it was also the source from which CDC pulled the weekly records. CDC’s weekly data export was performed using a Python script (coded at WSU) to implement an “Export Records” REDCap API method. The CDC representative was granted a key to access the WSU REDCap production project via the API. The export script was written to dynamically filter the exported record set to contain records that were new, or updated, since the last export. The export script also dynamically modified a SAS → template program, stored at CDC, to convert the raw REDCap export into a SAS formatted dataset.

### Field coding

It is important to distinguish RESP-LENS from a retrospective human-based extraction of data. Instead, this is an entirely computerized method designed to reduce human error from routine data extraction and entry. Each index and 30-day file transferred contains fields that exist in two categories based upon logic. The first category includes fields that should always have data present (such as age, gender, and race) while the second category includes fields that may or may not have data present (such as the results of viral testing). However, for the first category, the reality of large datasets is that the fields that should always be present are not always present; as may be the case for noncommunicative patients with no personal identification who satisfied an ARI definition but cannot state their age, gender or race. In both cases, the EMR leaves the field as empty, (also referred to as a “null” value). Thus, all data fields have three states: coded and known, coded and uncertain (in the case of a John Doe, where it is a known unknown), and missing (or null, which raises the possibility of other entry error). Additionally, fields may contain either categorical (test done or not done, result positive or negative) or numerical values (e.g., vital signs). To address this configuration, as a convention, we adopted a convention of coding “999” as an indication of “not done” for categorical fields and “-999” to indicate not done for numeric fields. The negative sign was added to allow analysts to easily exclude all missing results as none of the measurements recorded in the study would legitimately have values less than zero. For missing dates “1/1/1900” is input. Additional codes were added to address indeterminate results of tests. A deeper ambiguity occurs in the rare case where a test is ordered, and no result can be found. The leadership deemed that this situation was equivalent to no test done, and as such, these results fields are coded as “999” or “-999” as appropriate. These conventions all represent tradeoffs to capture as much robust data as possible while limiting the complexity and total number of data fields collected.

### Site-specific variations

Although all sites employ viral assays that use polymerase chain reaction detection, each site had numerous pathways within the EMR to order viral testing, and these order mechanisms could change with time, depending on supply chain issues. For example, one of the 24 sites has 18 unique orders to test for SARS-CoV-2, while another site only has four orders. Sites vary in their testing protocols including the use of multiplex viral testing and testing requirements for admitted patients. In some cases, the testing protocols change from weekdays to weekends. As part of the weekly organizational calls, representatives from CDC and the network management review relative changes in viral test ordering, and if a site has an outlier of more than a 50% change from prior weeks, an inquiry is made to the site. This has led to discovery of unrecognized changes in the electronic location of test ordering in 13 sites. As new diagnostic pathways and orders are developed at the site level, it will remain the sites' responsibility to identify these to avoid missing cases and diagnostic data. Additionally, many sites have had challenges with workforce turnover, particularly on the informatics side, which has led to occasional interruptions in data transmission. These examples reinforce the need for centralized, weekly human oversight to ensure complete and valid data.

### Unexpected lessons learned

One issue that delayed initial contracting was that certain sites, particularly those that represented large networks of hospitals, required their own data use agreements, which added up to six months to the onboarding process. Some patients have multiple identifiers in the EHR due to registration errors or a lack of identifiers during an emergency presentation (e.g., "Jane or John Doe”). Accordingly, we used the FIN which is unique to each individual and visit as the internal tracking mechanism for the index visit. This was used to then update the hash code at the 30-day follow-up as the unique patient identifier. This 30-day follow-up leads to one of the most frequent challenges to the detection of ARI cases, namely the delay to ICD coding associated with the action of a patient being discharged or admitted to the hospital. This results in an increase in ARI cases at the time of 30-day follow-up—meaning that four weeks later, there are approximately 4% more cases discovered than had an ARI-defining ED diagnosis. Other anomalies include out-of-range variables that persistently remain coded in the EMR, probably representing human error. One example is age > 120 years, observed in 0.001% of cases. Coding errors required ongoing editing such as the mistaken use of “999” instead of “-999” and “1/1/1990″ to document missing numerals and dates, respectively.

### Preliminary results

The publicly available interactive dashboard for RESP-LENS is available at https://www.cdc.gov/surveillance/resp-lens/dashboard.html. Although data collection began in late 2021, this dashboard includes data starting in October, 2022. As of August 20, 2023, the network has tracked 5,192,826 ED patients, of whom 873,905 (16.8%) had an ARI-defining ICD-10 code. Of these 873,905 patients, 63% had a diagnostic test for SARS-CoV-2 and 53.5% had a test for influenza; positivity for SARS-CoV-2 was 13.9% and for influenza A was 3.3%, respectively. The average age of patients was 41 years (SD 16), and 52% were reported in the EMR as female gender. The racial distribution of patients was diverse: 52% White, 27% Black, 2.3% Asian, 0.9% native Hawaiian or other Pacific origin, and 0.5% American Indian or Alaskan origin; 13.5% reported other or unknown race and 2.0% reported more than one raceMissing or empty data has occurred in < 1% of demographic and viral testing data. Fields that are most commonly missing from the EMR (i.e., no data to find) include body mass index (not documented in 49%) and vaccination status (not documented in 59%).

### Utility and discussion

RESP-LENS has two broad aims: 1) provide timely, systematic national surveillance for ARI including both virologic and clinical data for ED visits on a weekly basis, and 2) create the infrastructure and methodology to report the epidemiology of viral infections, and their subtypes where possible, and associate them with specific diagnoses and outcomes. The network provides data to detect new outbreaks, understand the magnitude and distribution of respiratory viral infections in patients seeking unscheduled care, follow the natural history and sequela of viral infections, and evaluate control strategies [7]. Among the first outcomes investigated will be the strength of the association between viral infections and the new diagnosis of venous thromboembolism. The data will allow stratification of illness severity, based upon admission location, need for intubation (from CPT coding), and mortality within 30 days. Additionally, with relatively minor changes in SQL coding at each site, this network can serve as a platform for surveillance of other syndromes.

The COVID-19 era highlighted current deficiencies in US surveillance networks. The 2022 Senate Homeland Security and Governmental Affairs Committee report on COVID-19 concluded that the U.S. failed to sufficiently invest in public health preparedness across multiple administrations, and that U.S. public health surveillance systems for monitoring and detecting emerging infectious diseases are inadequate, antiquated and fragmented [9]. To our knowledge, RESP-LENS is the first ED-based, national surveillance network to link virologic, clinical and disposition data for multiple respiratory viruses in a single platform that provides data on a weekly basis. The advantage of this method includes the consistency and transparency offered by the coding strategy and the ability to link clinical data elements at the visit and patient level and provide the information weekly to inform situational awareness and guide public health action. Weekly data collection from hospital ED EMRs likely captures a different patient sample (in demographic and infection severity) than may be captured by health departments. The methodology used also allows the comparison of the outcomes of cases with ARI-defining ED diagnoses who are tested for viruses with those who are not tested. While new standards for data interchange from EMRs (notably the FHIR standard) provide promise to simplify future surveillance and data aggregation, the methodologies used in RESP-LENS extend the existing clinical research infrastructure to capture quality data at scale with continuous oversight and input of clinician-scientists [10].

### Planned work and future directions

We will be able to integrate county-level CDC SVI and other health equity relevant exposure data, but not at the census tract level. This effort will leverage methodology used in Wayne State’s PHOENIX Virtual Data Warehouse, which has already assembled troves of social determinants of health data for ZIP codes, and has established standard operating procedures for integrating the information with investigator-generated person-level data [8]. We hope that publication of this protocol (and subsequent original research manuscripts) will enhance the visibility of RESP-LENS and encourage further investments to pursue deeper data, including viral genomic sequencing. Depending upon funding, we hope to link records to state immunization databases and compare our trends with other CDC databases.

### Limitations

In addition to the unexpected lessons described above, other challenges include site-to-site variations and constant changes in test ordering, and local guidance on which patients should receive testing. The current methodology also remains vulnerable to late or missing uploads because it requires a human to remember to upload the files. Accordingly, a next step will be the creation of methodology to automate the upload process at each site. RESP-LENS is constrained by what is hard-coded in Cerner and Epic; their “other” or “more than one” race categories provide only those designations, and do not provide further racial or ethnic background. We plan to link records to valid state immunization databases, but until then, vaccination status relies upon documentation in the electronic record, which is either missing, incomplete, or lacking detail such as date of administration and vaccine manufacturer. The 30-day outcomes are restricted to the results in the medical record, and local data health exchanges. Clearly, we could miss deaths or severe outcomes that occurred in facilities outside the local systems. Other limitations include lack of viral genotyping, and outcomes only at 30 days, and lack of control over the clinical decision to perform viral testing [11].

## Conclusion

The RESP-LENS national network provides weekly data describing the frequency and outcomes of patients with ARI-defining ED diagnoses in all 10 DHHS regions in the US. This network addresses the critical gap in accurate, timely pan-respiratory surveillance linking virologic and clinical data.

### Supplementary Information

**Additional file 1.**

**Additional file 2.**

## Data Availability

Consistent with the purpose of surveillance, key situational awareness variables including weekly frequency data for viral testing and results by age group and HHS region are made publicly available from the CDC on several venues. The datasets are available at https://recover-cdc.com/login, and a login and password will be provided by the corresponding author on reasonable request. Researchers interested in accessing the data for publication will be asked to submit a project proposal which the data use committee will review. Requests are handled using a structured form that reports the title of the work, the investigators, the purpose and the analytical plan. Authorship order is decided before granting access to data, with the order determined by prior and anticipated contributions by the proposed authors. The data use committee will oversee authorship.
