# Supplementary material for: Database derived from an electronic medical record-based surveillance network of US emergency department patients with acute respiratory illness
Source: BMC Med Inform Decis Mak. 2023 Oct 17;23:224. doi: 10.1186/s12911-023-02310-4 (PMC10580574; doi:10.1186/s12911-023-02310-4)
Supplement: Supplementary file 1 — Additional file 1. [file 12911_2023_2310_MOESM1_ESM.docx]

| Supplemental Table 1. Sites, site PIs and hospitals at each site (August, 2023) | | | | |
| --- | --- | --- | --- | --- |
| **Site Name** | **State** | **Name** | **Hospital** | **Hospital Code** |
| Beaumont Health (William Beaumont Hospital) | MI | Danielle Turner-Lawrence | Royal Oak | BE01 |
|  |  |  | Troy | BE02 |
|  |  |  | Dearborn | BE03 |
|  |  |  | Farmington Hills | BE04 |
|  |  |  | Grosse Pointe | BE05 |
|  |  |  | Taylor | BE06 |
|  |  |  | Trenton | BE07 |
|  |  |  | Wayne | BE08 |
|  | | | | |
| George Washington University (Medical Faculty Associates) | DC | Andrew Meltzer | George Washington University Hospital | GW01 |
|  |  |  | United Medical Center | GW02 |
|  |  |  | Inova Fairfax | GW03 |
|  |  |  | Washington Hospital Center | GW04 |
|  |  |  | Georgetown University | GW05 |
|  |  |  | Southern Maryland | GW06 |
|  | | | | |
| Hennepin County Medical Center | MN | Mike Puskarich | Hennepin County Medical Center | HM01 |
|  | | | | |
| Intermountain Medical Center (IHC Health Services, Inc) | UT | Joseph Bledsoe | Intermountain Medical Center | IM01 |
|  |  |  | Utah Valley | IM02 |
|  |  |  | Primary Children's | IM03 |
|  |  |  | LDS Hospital | IM04 |
|  |  |  | Dixie Regional Medical Center | IM05 |
|  |  |  | Logan Regional | IM06 |
|  |  |  | Riverton | IM07 |
|  |  |  | American Fork | IM08 |
|  |  |  | Alta View | IM09 |
|  |  |  | Cedar City | IM10 |
|  |  |  | Park City | IM11 |
|  |  |  | Sevier Valley | IM12 |
|  |  |  | Cassia Regional | IM13 |
|  |  |  | Orem Community | IM14 |
|  |  |  | Fillmore Community | IM15 |
|  |  |  | Heber Valley | IM16 |
|  |  |  | Delta Community | IM17 |
|  |  |  | Sanpete Valley | IM18 |
|  |  |  | Bear River Valley | IM19 |
|  |  |  | Garfield Memorial | IM20 |
|  |  |  | Layton | IM21 |
|  |  |  | McKay-Dee Hospital | IM22 |
|  | | | | |
| Massachusetts General Hospital (The General Hospital Corporation) | MA | Christopher Kabrhel | Massachusetts General Hospital | MG01 |
|  | | | | |
| Icahn School of Medicine at Mount Sinai | NY | Ka Ming Gordon Ngai | Mount Sinai Hospital | MS01 |
|  |  |  | Mount Sinai Brooklyn | MS02 |
|  |  |  | Mount Sinai Morningside | MS03 |
|  |  |  | Mount Sinai West | MS04 |
|  |  |  | Mount Sinai Queens | MS05 |
|  |  |  | Mount Sinai Beth Israel | MS06 |
|  |  |  | Elmhurst Hospital | MS07 |
|  | | | | |
| Integrated Emergency Services | TX | James d'Ettiene | John Peter Smith Hospital | JP01 |
|  |  |  |  |  |
| Oregon Health & Science University | OR | Esther Choo | OHSU Adult ED | OH01 |
|  |  |  | OHSU Pediatric ED | OU02 |
|  |  |  | Columbia Memorial ED | OH03 |
|  |  |  | Hillsboro ED | OH04 |
|  |  |  | Adventist ED | OH05 |
|  | | | | |
| Penn State Hershey Medical Center | PA | Jeff Lubin | Penn State Hershey Medical Center | PS01 |
|  | | | | |
| University of Florida - Jacksonville | FL | Faheem Guirgis | UF Jacksonville Downtown ED | UF01 |
|  |  |  | UF Jacksonville North | UF02 |
|  |  |  | UF Gainesville Main ED | UF03 |
|  |  |  | UF Health Kanapaha FSED | UF04 |
|  |  |  | UF Health Springhill FSED | UF05 |
|  | | | | |
| Centura Health System Colorado | CO | Michael Roshon | Avista Adventist Hospital | CO01 |
|  |  |  | St. Anthony Hospital | CO02 |
|  |  |  | St. Anthony North Hospital | CO03 |
|  |  |  | St. Anthony Summit Medical Center | CO04 |
|  |  |  | Littleton Adventist Hospital | CO05 |
|  |  |  | Parker Adventist Hospital | CO06 |
|  |  |  | Porter Adventists Hospital | CO07 |
|  |  |  | OrthoColorado Hospital | CO08 |
|  |  |  | Penrose Hospital | CO09 |
|  |  |  | St. Francis Medical Center | CO10 |
|  |  |  | St. Mary-Corwin Medical Center | CO11 |
|  |  |  | St. Thomas More Hospital | CO12 |
|  |  |  | St. Catherine Hospital | CO13 |
|  |  |  | Mercy Hospital | CO14 |
|  |  |  | Longmont United Hospital | CO15 |
|  |  |  | Castle Rock Adventist Hospital | CO16 |
|  |  |  | Bob Wilson Memorial Hospital | CO17 |
|  | | | | |
| The Ohio State University | OH | Katherine Buck | University Hospital | OS01 |
|  |  |  | East Hospital | OS02 |
|  | | | | |
| Trustees of Indiana University | IN | Benton Hunter | IU Health Methodist Hospital | IU01 |
|  |  |  | IU Health Bloomington Hospital | IU02 |
|  |  |  | IU Health Bedford Hospital | IU03 |
|  |  |  | IU Health Morgan Hospital | IU04 |
|  |  |  | IU Health White Memorial Hospital | IU05 |
|  |  |  | Sydney & Lois Eskenazi Hospital | IU06 |
|  |  |  | IU Health Jay Hospital | IU07 |
|  |  |  | IU Health University Hospital | IU08 |
|  |  |  | Riley Hospital for Children | IU09 |
|  |  |  | IU Health North Hospital | IU10 |
|  |  |  | IU Health West Hospital | IU11 |
|  |  |  | IU Health Arnett Hospital | IU12 |
|  |  |  | IU Health Ball Memorial Hospital | IU13 |
|  |  |  | IU Health Blackford Hospital | IU14 |
|  |  |  | IU Health La Porte Hospital | IU15 |
|  |  |  | IU Health Paoli Hospital | IU16 |
|  |  |  | IU Health Saxony Hospital | IU17 |
|  |  |  | IU Health Starke Hospital | IU18 |
|  |  |  | IU Health Tipton Hospital | IU19 |
|  |  |  | IU Health Frankfort Hospital | IU20 |
|  | | | | |
| University of California San Diego | CA | Edward Castillo | UCSD Hillcrest Medical Center | SD01 |
|  |  |  | UCSD La Jolla Medical Center | SD02 |
|  | | | | |
| University of Chicago | IL | David Beiser | University of Chicago Medicine | CM01 |
|  | | | | |
| University of Colorado | CO | Kristen Nordenholz | All UC Health Hospitals | UC01 |
|  | | | | |
| University of Iowa | IO | Bryan Wilson | University of Iowa Hospitals and Clinics | UI01 |
|  | | | | |
| University of Mississippi | MS | James Galbraith | University Hospital | MI01 |
|  |  |  | Wallace Conerly Critical Care Hospital | MI02 |
|  |  |  | Winfred L. Wiser Hospital for Women and Infants | MI03 |
|  |  |  | Blair E. Batson Children's Hospital | MI04 |
|  |  |  | UMMC Holmes County | MI05 |
|  |  |  | UMMC Grenada | MI06 |
|  | | | | |
| University of Utah Health Sciences Center | UT | Chris Kelly | University of Utah Hospital | UU01 |
|  |  |  | South Jordan Health Center | UU02 |
|  | | | | |
| University of Wisconsin - Madison- System (The Board of Regents of the) | WI | Michael Pulia | University of Wisconsin Hospital | UW01 |
|  |  |  | The American Center | UW02 |
|  | | | | |
| University Medical Center New Orleans | LA | Stephen Lim | University Medical Center New Orleans | LS01 |
|  |  |  | Touro Infirmary Hospital | LS02 |
|  |  |  | West Jefferson Medical Center | LS03 |
|  |  |  | New Orleans East Hospital | LS04 |
|  | | | | |
| UT Southwestern | TX | Joby Thoppil | Clements University Hospital | UT01 |
|  |  |  | Parkland Hospital | UT02 |
|  | | | | |
| Washington University in St. Louis | MO | Stacey House | Barnes-Jewish Hospital | WU01 |
|  |  |  | Missouri Baptist Hospital | WU02 |
|  | | | | |
| West Virginia University | WV | Justine Pagenhardt | Ruby Memorial Hospital | WV01 |
|  | | | | |
| University of California - San Francisco | CA | Israel Green-Hopkins | UCSF Mission Bay Benioff Children's Hospital | SF01 |
|  |  |  | UCSF Parnassus | SF02 |

Supplemental Table 2. International classification of diseases 10 identifiers used to locate patients with acute respiratory infection.

| **ICD 10 Code** | **ICD 10 Description** |
| --- | --- |
| A22.1 | Pulmonary anthrax |
| A37.00 | Whooping cough due to Bordetella Pertussis without Pneumonia |
| A37.01 | Whooping cough: Bordetella pertussis, pneumonia |
| A37.10 | Whooping cough due to Bordetella parapertussis without pneumonia |
| A37.11 | Whooping cough: B. parapertussis, pneumonia |
| A37.80 | Whooping cough due to other Bordetella species without pneumonia |
| A37.81 | Whooping cough: other Bordetella, pneumonia |
| A37.90 | Whooping cough, unspecified species without pneumonia |
| A37.91 | Whooping cough, unspecified species, pneumonia |
| A48.1 | Legionnaires’ disease |
| B25.0 | Cytomegaloviral pneumonitis |
| B34.2 | Coronavirus infection, unspecified |
| B34.9 | Viral infection, unspecified |
| B44.0 | Invasive pulmonary aspergillosis |
| B44.81 | Allergic bronchopulmonary aspergillosis |
| B44.9 | Aspergillosis, unspecified |
| B97.2 | Coronavirus as the cause of diseases classified elsewhere |
| B97.4 | Respiratory syncytial virus as the cause of diseases classified elsewhere |
| J00 | Acute nasopharyngitis (common cold) |
| J01.00 | Acute maxillary sinusitis, unspecified |
| J01.01 | Acute recurrent maxillary sinusitis |
| J01.10 | Acute frontal sinusitis, unspecified |
| J01.11 | Acute recurrent frontal sinusitis |
| J01.20 | Acute ethmoidal sinusitis, unspecified |
| J01.21 | Acute recurrent ethmoidal sinusitis |
| J01.30 | Acute sphenoidal sinusitis, unspecified |
| J01.31 | Acute recurrent sphenoidal sinusitis |
| J01.40 | Acute pansinusitis, unspecified |
| J01.41 | Acute recurrent pansinusitis |
| J01.80 | Other acute sinusitis |
| J01.81 | Other acute recurrent sinusitis |
| J01.90 | Acute sinusitis, unspecified |
| J01.91 | Acute recurrent sinusitis, unspecified |
| J02.0 | Streptococcal pharyngitis |
| J02.8 | Acute pharyngitis due to other specified organisms |
| J02.9 | Acute pharyngitis, unspecified |
| J03.00 | Acute streptococcal tonsillitis, unspecified |
| J03.01 | Acute recurrent streptococcal tonsillitis |
| J03.80 | Acute tonsillitis due to other specified organisms |
| J03.81 | Acute recurrent tonsillitis due to other specified organisms |
| J03.90 | Acute tonsillitis, unspecified |
| J03.91 | Acute recurrent tonsillitis, unspecified |
| J04.0 | Acute laryngitis |
| J04.10 | Acute tracheitis without obstruction |
| J04.11 | Acute tracheitis with obstruction |
| J04.2 | Acute laryngotracheitis |
| J04.30 | Supraglottitis, unspecified, without obstruction |
| J04.31 | Supraglottitis, unspecified, with obstruction |
| J05.0 | Acute obstructive laryngitis [croup] |
| J05.10 | Acute epiglottitis without obstruction |
| J05.11 | Acute epiglottitis with obstruction |
| J06.0 | Acute laryngopharyngitis |
| J06.9 | Acute upper respiratory infection, unspecified |
| J09.X | Influenza due to identified novel influenza A virus - all sub codes |
| J10.X | Influenza due to other identified influenza virus - all sub codes |
| J11.X | Influenza due to unidentified influenza virus - all sub codes |
| J12.0 | Adenoviral pneumonia |
| J12.1 | Respiratory syncytial virus pneumonia |
| J12.2 | Parainfluenza virus pneumonia |
| J12.3 | Human metapneumovirus pneumonia |
| J12.81 | Pneumonia due to SARS-associated coronavirus |
| J12.82 | Pneumonia due to COVID-19 / SARS CoV-2 |
| J12.89 | Other viral pneumonia |
| J12.9 | Viral pneumonia, unspecified |
| J13 | Pneumonia due to Streptococcus pneumoniae |
| J14 | Pneumonia due to Hemophilus influenzae |
| J15.0 | Pneumonia due to Klebsiella pneumoniae |
| J15.1 | Pneumonia due to Pseudomonas |
| J15.20 | Pneumonia due to staphylococcus, unspecified |
| J15.211 | Pneumonia due to Methicillin susceptible Staphylococcus aureus |
| J15.212 | Pneumonia due to Methicillin resistant Staphylococcus aureus |
| J15.29 | Pneumonia due to other staphylococcus |
| J15.3 | Pneumonia due to streptococcus, group B |
| J15.4 | Pneumonia due to other streptococci |
| J15.5 | Pneumonia due to Escherichia coli |
| J15.6 | Pneumonia due to other Gram-negative bacteria |
| J15.7 | Pneumonia due to Mycoplasma pneumoniae |
| J15.8 | Pneumonia due to other specified bacteria |
| J15.9 | Unspecified bacterial pneumonia |
| J16.0 | Chlamydial pneumonia |
| J16.8 | Pneumonia due to other specified infectious organisms |
| J17 | Pneumonia in diseases classified elsewhere |
| J18.0 | Bronchopneumonia, unspecified organism |
| J18.1 | Lobar pneumonia, unspecified organism |
| J18.2 | Hypostatic pneumonia, unspecified organism |
| J18.8 | Other pneumonia, unspecified organism |
| J18.9 | Pneumonia, unspecified organism |
| J20.0 | Acute bronchitis due to Mycoplasma pneumoniae |
| J20.1 | Acute bronchitis due to Hemophilus influenzae |
| J20.2 | Acute bronchitis due to streptococcus |
| J20.3 | Acute bronchitis due to coxsackievirus |
| J20.4 | Acute bronchitis due to parainfluenza virus |
| J20.5 | Acute bronchitis due to respiratory syncytial virus |
| J20.6 | Acute bronchitis due to rhinovirus |
| J20.7 | Acute bronchitis due to echovirus |
| J20.8 | Acute bronchitis due to other specified organisms |
| J20.9 | Acute bronchitis, unspecified |
| J21.0 | Acute bronchiolitis due to respiratory syncytial virus |
| J21.1 | Acute bronchiolitis due to human metapneumovirus |
| J21.8 | Acute bronchiolitis due to other specified organisms |
| J21.9 | Acute bronchiolitis, unspecified |
| J22 | Unspecified acute lower respiratory tract infection |
| J39.8 | Other specified diseases of upper respiratory tract |
| J40 | Bronchitis, not specified as acute or chronic |
| J47.9 | Bronchiectasis with acute exacerbation |
| J80 | Acute Respiratory Distress Syndrome |
| J85.1 | Abscess of lung with pneumonia |
| J95.821 | Acute postprocedural respiratory failure |
| J96.0 | Acute respiratory failure |
| J96.00 | Acute respiratory failure, unspecified whether with hypoxia or hypercapnia |
| J96.01 | Acute respiratory failure with hypoxia |
| J96.02 | Acute respiratory failure with hypercapnia |
| J96.2 | Acute and chronic respiratory failure |
| J96.20 | Acute and chronic respiratory failure, unspecified whether with hypoxia or hypercapnia |
| J96.21 | Acute and chronic respiratory failure with hypoxia |
| J96.22 | Acute and chronic respiratory failure with hypercapnia |
| J96.91 | Respiratory failure, unspecified with hypoxia |
| J98.8 | Other specified respiratory disorders |
| R05 | Cough |
| R06.00 | Dyspnea, unspecified plus fever |
| R06.02 | Shortness of breath |
| R06.03 | Acute respiratory distress |
| R09.02 | Hypoxemia |
| R09.2 | Respiratory arrest |
| R43.0 | Anosmia |
| R43.1 | Parosmia |
| R43.2 | Parageusia |
| R50.9 | Fever, unspecified |
| U07.1 | COVID-19, virus identified |
| U07.2 | COVID-19, virus not identified |
