# Supplementary material for: Database derived from an electronic medical record-based surveillance network of US emergency department patients with acute respiratory illness
Source: BMC Med Inform Decis Mak. 2023 Oct 17;23:224. doi: 10.1186/s12911-023-02310-4 (PMC10580574; doi:10.1186/s12911-023-02310-4)
Supplement: Supplementary file 2 — Additional file 2. [file 12911_2023_2310_MOESM2_ESM.pdf]

Visit Information

Record ID	______________________________
Medical record number (to be removed upon export)	______________________________
Financial indicator number (the "FIN"; to be removed upon export)	______________________________
Hashcode of medical record number (to be kept upon export)	______________________________
Date of service	______________________________

State (this is for the hospital of the index ED visit)

- ◯ AK
- ◯ AL
- ◯ AZ
- ◯ AR
- ◯ CA
- ◯ CO
- ◯ CT
- ◯ DC
- ◯ DE
- ◯ FL
- ◯ GA
- ◯ HI
- ◯ ID
- ◯ IL
- ◯ IN
- ◯ IA
- ◯ KS
- ◯ KY
- ◯ LA
- ◯ ME
- ◯ MD
- ◯ MA
- ◯ MI
- ◯ MN
- ◯ MS
- ◯ MO
- ◯ MT
- ◯ NE
- ◯ NV
- ◯ NH
- ◯ NJ
- ◯ NM
- ◯ NY
- ◯ NC
- ◯ ND
- ◯ OH
- ◯ OK
- ◯ OR
- ◯ PA
- ◯ RI
- ◯ SC
- ◯ SD
- ◯ TN
- ◯ TX
- ◯ UT
- ◯ VT
- ◯ VA
- ◯ WA
- ◯ WV
- ◯ WI
- ◯ WY

First 4 of zip code - patient address (first 4 digits only, if you enter 5 it will give you an error)

- ◯ 0050
- ◯ 0054
- ◯ 0060
- ◯ 0061
- ◯ 0062
- ◯ 0063
- ◯ 0064
- ◯ 0065
- ◯ 0066
- ◯ 0067
- ◯ 0068
- ◯ 0069
- ◯ 0070
- ◯ 0071
- ◯ 0072
- ◯ 0073
- ◯ 0074
- ◯ 0075
- ◯ 0076
- ◯ 0077
- ◯ 0078
- ◯ 0079
- ◯ 0080
- ◯ 0082
- ◯ 0083
- ◯ 0084
- ◯ 0085
- ◯ 0090
- ◯ 0091
- ◯ 0092
- ◯ 0093
- ◯ 0094
- ◯ 0095
- ◯ 0096
- ◯ 0097
- ◯ 0098
- ◯ 0100
- ◯ 0101
- ◯ 0102
- ◯ 0103
- ◯ 0104
- ◯ 0105
- ◯ 0106
- ◯ 0107
- ◯ 0108
- ◯ 0109
- ◯ 0110
- ◯ 0111
- ◯ 0112
- ◯ 0113
- ◯ 0114
- ◯ 0115
- ◯ 0119
- ◯ 0120
- ◯ 0122
- ◯ 0123
- ◯ 0124
- ◯ 0125
- ◯ 0126
- ◯ 0127
- ◯ 0130
- ◯ 0133
- ◯ 0134
- ◯ 0135
- ◯ 0136
- ◯ 0137
- ◯ 0138
- ◯ 0142
- ◯ 0143

◯ 0144
◯ 0145
◯ 0146
◯ 0147
◯ 0150
◯ 0151
◯ 0152
◯ 0153
◯ 0154
◯ 0155
◯ 0156
◯ 0157
◯ 0158
◯ 0159
◯ 0160
◯ 0161
◯ 0165
◯ 0170
◯ 0171
◯ 0172
◯ 0173
◯ 0174
◯ 0175
◯ 0176
◯ 0177
◯ 0178
◯ 0180
◯ 0181
◯ 0182
◯ 0183
◯ 0184
◯ 0185
◯ 0186
◯ 0187
◯ 0188
◯ 0189
◯ 0190
◯ 0191
◯ 0192
◯ 0193
◯ 0194
◯ 0195
◯ 0196
◯ 0197
◯ 0198
◯ 0201
◯ 0202
◯ 0203
◯ 0204
◯ 0205
◯ 0206
◯ 0207
◯ 0208
◯ 0209
◯ 0210
◯ 0211
◯ 0212
◯ 0213
◯ 0214
◯ 0215
◯ 0216
◯ 0217
◯ 0218
◯ 0219
◯ 0220
◯ 0221
◯ 0222
◯ 0223
◯ 0224
◯ 0226
◯ 0228

- ◯ 0229
- ◯ 0230
- ◯ 0232
- ◯ 0233
- ◯ 0234
- ◯ 0235
- ◯ 0236
- ◯ 0237
- ◯ 0238
- ◯ 0242
- ◯ 0244
- ◯ 0245
- ◯ 0246
- ◯ 0247
- ◯ 0248
- ◯ 0249
- ◯ 0253
- ◯ 0254
- ◯ 0255
- ◯ 0256
- ◯ 0257
- ◯ 0258
- ◯ 0260
- ◯ 0263
- ◯ 0264
- ◯ 0265
- ◯ 0266
- ◯ 0267
- ◯ 0270
- ◯ 0271
- ◯ 0272
- ◯ 0273
- ◯ 0274
- ◯ 0276
- ◯ 0277
- ◯ 0278
- ◯ 0279
- ◯ 0280
- ◯ 0281
- ◯ 0282
- ◯ 0283
- ◯ 0284
- ◯ 0285
- ◯ 0286
- ◯ 0287
- ◯ 0288
- ◯ 0289
- ◯ 0290
- ◯ 0291
- ◯ 0292
- ◯ 0294
- ◯ 0303
- ◯ 0304
- ◯ 0305
- ◯ 0306
- ◯ 0307
- ◯ 0308
- ◯ 0310
- ◯ 0311
- ◯ 0321
- ◯ 0322
- ◯ 0323
- ◯ 0324
- ◯ 0325
- ◯ 0326
- ◯ 0327
- ◯ 0328
- ◯ 0329
- ◯ 0330
- ◯ 0343
- ◯ 0344

◯ 0345
◯ 0346
◯ 0347
◯ 0356
◯ 0357
◯ 0358
◯ 0359
◯ 0360
◯ 0374
◯ 0375
◯ 0376
◯ 0377
◯ 0378
◯ 0380
◯ 0381
◯ 0382
◯ 0383
◯ 0384
◯ 0385
◯ 0386
◯ 0387
◯ 0388
◯ 0389
◯ 0390
◯ 0391
◯ 0400
◯ 0401
◯ 0402
◯ 0403
◯ 0404
◯ 0405
◯ 0406
◯ 0407
◯ 0408
◯ 0409
◯ 0410
◯ 0411
◯ 0412
◯ 0421
◯ 0422
◯ 0423
◯ 0424
◯ 0425
◯ 0426
◯ 0427
◯ 0428
◯ 0429
◯ 0433
◯ 0434
◯ 0435
◯ 0436
◯ 0440
◯ 0441
◯ 0442
◯ 0443
◯ 0444
◯ 0445
◯ 0446
◯ 0447
◯ 0448
◯ 0449
◯ 0453
◯ 0454
◯ 0455
◯ 0456
◯ 0457
◯ 0460
◯ 0461
◯ 0462
◯ 0463
◯ 0464

- ◯ 0465
- ◯ 0466
- ◯ 0467
- ◯ 0468
- ◯ 0469
- ◯ 0473
- ◯ 0474
- ◯ 0475
- ◯ 0476
- ◯ 0477
- ◯ 0478
- ◯ 0484
- ◯ 0485
- ◯ 0486
- ◯ 0490
- ◯ 0491
- ◯ 0492
- ◯ 0493
- ◯ 0494
- ◯ 0495
- ◯ 0496
- ◯ 0497
- ◯ 0498
- ◯ 0499
- ◯ 0500
- ◯ 0503
- ◯ 0504
- ◯ 0505
- ◯ 0506
- ◯ 0507
- ◯ 0508
- ◯ 0509
- ◯ 0510
- ◯ 0514
- ◯ 0515
- ◯ 0516
- ◯ 0520
- ◯ 0525
- ◯ 0526
- ◯ 0530
- ◯ 0534
- ◯ 0535
- ◯ 0536
- ◯ 0540
- ◯ 0543
- ◯ 0544
- ◯ 0545
- ◯ 0546
- ◯ 0547
- ◯ 0548
- ◯ 0549
- ◯ 0550
- ◯ 0560
- ◯ 0562
- ◯ 0563
- ◯ 0564
- ◯ 0565
- ◯ 0566
- ◯ 0567
- ◯ 0568
- ◯ 0570
- ◯ 0573
- ◯ 0574
- ◯ 0575
- ◯ 0576
- ◯ 0577
- ◯ 0581
- ◯ 0582
- ◯ 0583
- ◯ 0584
- ◯ 0585

- ◯ 0586
- ◯ 0587
- ◯ 0590
- ◯ 0600
- ◯ 0601
- ◯ 0602
- ◯ 0603
- ◯ 0604
- ◯ 0605
- ◯ 0606
- ◯ 0607
- ◯ 0608
- ◯ 0609
- ◯ 0610
- ◯ 0611
- ◯ 0612
- ◯ 0613
- ◯ 0614
- ◯ 0615
- ◯ 0616
- ◯ 0617
- ◯ 0618
- ◯ 0619
- ◯ 0622
- ◯ 0623
- ◯ 0624
- ◯ 0625
- ◯ 0626
- ◯ 0627
- ◯ 0628
- ◯ 0632
- ◯ 0633
- ◯ 0634
- ◯ 0635
- ◯ 0636
- ◯ 0637
- ◯ 0638
- ◯ 0639
- ◯ 0640
- ◯ 0641
- ◯ 0642
- ◯ 0643
- ◯ 0644
- ◯ 0645
- ◯ 0646
- ◯ 0647
- ◯ 0648
- ◯ 0649
- ◯ 0650
- ◯ 0651
- ◯ 0652
- ◯ 0653
- ◯ 0654
- ◯ 0660
- ◯ 0661
- ◯ 0667
- ◯ 0669
- ◯ 0670
- ◯ 0671
- ◯ 0672
- ◯ 0674
- ◯ 0675
- ◯ 0676
- ◯ 0677
- ◯ 0678
- ◯ 0679
- ◯ 0680
- ◯ 0681
- ◯ 0682
- ◯ 0683
- ◯ 0684

◯ 0685
◯ 0686
◯ 0687
◯ 0688
◯ 0689
◯ 0690
◯ 0691
◯ 0692
◯ 0700
◯ 0701
◯ 0702
◯ 0703
◯ 0704
◯ 0705
◯ 0706
◯ 0707
◯ 0708
◯ 0709
◯ 0710
◯ 0711
◯ 0717
◯ 0718
◯ 0719
◯ 0720
◯ 0730
◯ 0731
◯ 0739
◯ 0740
◯ 0741
◯ 0742
◯ 0743
◯ 0744
◯ 0745
◯ 0746
◯ 0747
◯ 0748
◯ 0749
◯ 0750
◯ 0751
◯ 0752
◯ 0753
◯ 0754
◯ 0760
◯ 0762
◯ 0763
◯ 0764
◯ 0765
◯ 0766
◯ 0767
◯ 0769
◯ 0770
◯ 0771
◯ 0772
◯ 0773
◯ 0774
◯ 0775
◯ 0776
◯ 0779
◯ 0780
◯ 0782
◯ 0783
◯ 0784
◯ 0785
◯ 0786
◯ 0787
◯ 0788
◯ 0789
◯ 0790
◯ 0792
◯ 0793
◯ 0794

- ◯ 0795
- ◯ 0796
- ◯ 0797
- ◯ 0798
- ◯ 0799
- ◯ 0800
- ◯ 0801
- ◯ 0802
- ◯ 0803
- ◯ 0804
- ◯ 0805
- ◯ 0806
- ◯ 0807
- ◯ 0808
- ◯ 0809
- ◯ 0810
- ◯ 0811
- ◯ 0820
- ◯ 0821
- ◯ 0822
- ◯ 0823
- ◯ 0824
- ◯ 0825
- ◯ 0826
- ◯ 0827
- ◯ 0830
- ◯ 0831
- ◯ 0832
- ◯ 0833
- ◯ 0834
- ◯ 0835
- ◯ 0836
- ◯ 0840
- ◯ 0850
- ◯ 0851
- ◯ 0852
- ◯ 0853
- ◯ 0854
- ◯ 0855
- ◯ 0856
- ◯ 0860
- ◯ 0861
- ◯ 0862
- ◯ 0863
- ◯ 0864
- ◯ 0865
- ◯ 0866
- ◯ 0869
- ◯ 0870
- ◯ 0872
- ◯ 0873
- ◯ 0874
- ◯ 0875
- ◯ 0880
- ◯ 0881
- ◯ 0882
- ◯ 0883
- ◯ 0884
- ◯ 0885
- ◯ 0886
- ◯ 0887
- ◯ 0888
- ◯ 0889
- ◯ 0890
- ◯ 0893
- ◯ 0898
- ◯ 0900
- ◯ 0901
- ◯ 0902
- ◯ 0903
- ◯ 0904

◯ 0905
◯ 0906
◯ 0907
◯ 0909
◯ 0910
◯ 0911
◯ 0912
◯ 0913
◯ 0914
◯ 0915
◯ 0916
◯ 0917
◯ 0918
◯ 0920
◯ 0921
◯ 0922
◯ 0924
◯ 0925
◯ 0926
◯ 0927
◯ 0928
◯ 0929
◯ 0930
◯ 0931
◯ 0932
◯ 0933
◯ 0934
◯ 0935
◯ 0936
◯ 0937
◯ 0938
◯ 0940
◯ 0941
◯ 0942
◯ 0944
◯ 0945
◯ 0946
◯ 0947
◯ 0948
◯ 0949
◯ 0950
◯ 0951
◯ 0952
◯ 0953
◯ 0954
◯ 0955
◯ 0956
◯ 0957
◯ 0958
◯ 0959
◯ 0960
◯ 0961
◯ 0962
◯ 0963
◯ 0964
◯ 0970
◯ 0971
◯ 0972
◯ 0973
◯ 0974
◯ 0975
◯ 0976
◯ 0977
◯ 0978
◯ 0980
◯ 0981
◯ 0982
◯ 0983
◯ 0984
◯ 0985
◯ 0986

◯ 0987
◯ 0988
◯ 0989
◯ 0990
◯ 0991
◯ 0997
◯ 1000
◯ 1001
◯ 1002
◯ 1003
◯ 1004
◯ 1005
◯ 1006
◯ 1007
◯ 1008
◯ 1009
◯ 1010
◯ 1011
◯ 1012
◯ 1013
◯ 1015
◯ 1016
◯ 1017
◯ 1018
◯ 1019
◯ 1020
◯ 1021
◯ 1024
◯ 1025
◯ 1026
◯ 1027
◯ 1028
◯ 1030
◯ 1031
◯ 1045
◯ 1046
◯ 1047
◯ 1050
◯ 1051
◯ 1052
◯ 1053
◯ 1054
◯ 1055
◯ 1056
◯ 1057
◯ 1058
◯ 1059
◯ 1060
◯ 1061
◯ 1070
◯ 1071
◯ 1080
◯ 1090
◯ 1091
◯ 1092
◯ 1093
◯ 1094
◯ 1095
◯ 1096
◯ 1097
◯ 1098
◯ 1099
◯ 1100
◯ 1101
◯ 1102
◯ 1103
◯ 1104
◯ 1105
◯ 1109
◯ 1110
◯ 1112

◯ 1120
◯ 1121
◯ 1122
◯ 1123
◯ 1124
◯ 1125
◯ 1135
◯ 1136
◯ 1137
◯ 1138
◯ 1140
◯ 1141
◯ 1142
◯ 1143
◯ 1145
◯ 1149
◯ 1150
◯ 1151
◯ 1152
◯ 1153
◯ 1154
◯ 1155
◯ 1156
◯ 1157
◯ 1158
◯ 1159
◯ 1169
◯ 1170
◯ 1171
◯ 1172
◯ 1173
◯ 1174
◯ 1175
◯ 1176
◯ 1177
◯ 1178
◯ 1179
◯ 1180
◯ 1181
◯ 1185
◯ 1190
◯ 1193
◯ 1194
◯ 1195
◯ 1196
◯ 1197
◯ 1198
◯ 1200
◯ 1201
◯ 1202
◯ 1203
◯ 1204
◯ 1205
◯ 1206
◯ 1207
◯ 1208
◯ 1209
◯ 1210
◯ 1211
◯ 1212
◯ 1213
◯ 1214
◯ 1215
◯ 1216
◯ 1217
◯ 1218
◯ 1219
◯ 1220
◯ 1221
◯ 1222
◯ 1223

◯ 1224
◯ 1225
◯ 1226
◯ 1228
◯ 1230
◯ 1232
◯ 1234
◯ 1240
◯ 1241
◯ 1242
◯ 1243
◯ 1244
◯ 1245
◯ 1246
◯ 1247
◯ 1248
◯ 1249
◯ 1250
◯ 1251
◯ 1252
◯ 1253
◯ 1254
◯ 1255
◯ 1256
◯ 1257
◯ 1258
◯ 1259
◯ 1260
◯ 1270
◯ 1271
◯ 1272
◯ 1273
◯ 1274
◯ 1275
◯ 1276
◯ 1277
◯ 1278
◯ 1279
◯ 1280
◯ 1281
◯ 1282
◯ 1283
◯ 1284
◯ 1285
◯ 1286
◯ 1287
◯ 1288
◯ 1290
◯ 1291
◯ 1292
◯ 1293
◯ 1294
◯ 1295
◯ 1296
◯ 1297
◯ 1298
◯ 1299
◯ 1302
◯ 1303
◯ 1304
◯ 1305
◯ 1306
◯ 1307
◯ 1308
◯ 1309
◯ 1310
◯ 1311
◯ 1312
◯ 1313
◯ 1314
◯ 1315

◯ 1316
◯ 1320
◯ 1321
◯ 1322
◯ 1323
◯ 1324
◯ 1325
◯ 1326
◯ 1329
◯ 1330
◯ 1331
◯ 1332
◯ 1333
◯ 1334
◯ 1335
◯ 1336
◯ 1340
◯ 1341
◯ 1342
◯ 1343
◯ 1344
◯ 1345
◯ 1346
◯ 1347
◯ 1348
◯ 1349
◯ 1350
◯ 1359
◯ 1360
◯ 1361
◯ 1362
◯ 1363
◯ 1364
◯ 1365
◯ 1366
◯ 1367
◯ 1368
◯ 1369
◯ 1373
◯ 1374
◯ 1375
◯ 1376
◯ 1377
◯ 1378
◯ 1379
◯ 1380
◯ 1381
◯ 1382
◯ 1383
◯ 1384
◯ 1385
◯ 1386
◯ 1390
◯ 1400
◯ 1401
◯ 1402
◯ 1403
◯ 1404
◯ 1405
◯ 1406
◯ 1407
◯ 1408
◯ 1409
◯ 1410
◯ 1411
◯ 1412
◯ 1413
◯ 1414
◯ 1415
◯ 1416
◯ 1417

◯ 1420
◯ 1421
◯ 1422
◯ 1423
◯ 1424
◯ 1426
◯ 1427
◯ 1428
◯ 1430
◯ 1441
◯ 1442
◯ 1443
◯ 1444
◯ 1445
◯ 1446
◯ 1447
◯ 1448
◯ 1450
◯ 1451
◯ 1452
◯ 1453
◯ 1454
◯ 1455
◯ 1456
◯ 1457
◯ 1458
◯ 1459
◯ 1460
◯ 1461
◯ 1462
◯ 1463
◯ 1464
◯ 1465
◯ 1469
◯ 1470
◯ 1471
◯ 1472
◯ 1473
◯ 1474
◯ 1475
◯ 1476
◯ 1477
◯ 1478
◯ 1480
◯ 1481
◯ 1482
◯ 1483
◯ 1484
◯ 1485
◯ 1486
◯ 1487
◯ 1488
◯ 1489
◯ 1490
◯ 1500
◯ 1501
◯ 1502
◯ 1503
◯ 1504
◯ 1505
◯ 1506
◯ 1507
◯ 1508
◯ 1509
◯ 1510
◯ 1511
◯ 1512
◯ 1513
◯ 1514
◯ 1520
◯ 1521

◯ 1522
◯ 1523
◯ 1524
◯ 1525
◯ 1526
◯ 1527
◯ 1528
◯ 1529
◯ 1530
◯ 1531
◯ 1532
◯ 1533
◯ 1534
◯ 1535
◯ 1536
◯ 1537
◯ 1538
◯ 1540
◯ 1541
◯ 1542
◯ 1543
◯ 1544
◯ 1545
◯ 1546
◯ 1547
◯ 1548
◯ 1549
◯ 1550
◯ 1551
◯ 1552
◯ 1553
◯ 1554
◯ 1555
◯ 1556
◯ 1560
◯ 1561
◯ 1562
◯ 1563
◯ 1564
◯ 1565
◯ 1566
◯ 1567
◯ 1568
◯ 1569
◯ 1570
◯ 1571
◯ 1572
◯ 1573
◯ 1574
◯ 1575
◯ 1576
◯ 1577
◯ 1578
◯ 1580
◯ 1582
◯ 1583
◯ 1584
◯ 1585
◯ 1586
◯ 1587
◯ 1590
◯ 1591
◯ 1592
◯ 1593
◯ 1594
◯ 1595
◯ 1596
◯ 1600
◯ 1601
◯ 1602
◯ 1603

◯ 1604
◯ 1605
◯ 1606
◯ 1610
◯ 1611
◯ 1612
◯ 1613
◯ 1614
◯ 1615
◯ 1616
◯ 1617
◯ 1620
◯ 1621
◯ 1622
◯ 1623
◯ 1624
◯ 1625
◯ 1626
◯ 1630
◯ 1631
◯ 1632
◯ 1633
◯ 1634
◯ 1635
◯ 1636
◯ 1637
◯ 1638
◯ 1640
◯ 1641
◯ 1642
◯ 1643
◯ 1644
◯ 1647
◯ 1650
◯ 1651
◯ 1652
◯ 1653
◯ 1654
◯ 1655
◯ 1656
◯ 1660
◯ 1661
◯ 1662
◯ 1663
◯ 1664
◯ 1665
◯ 1666
◯ 1667
◯ 1668
◯ 1669
◯ 1670
◯ 1672
◯ 1673
◯ 1674
◯ 1675
◯ 1680
◯ 1682
◯ 1683
◯ 1684
◯ 1685
◯ 1686
◯ 1687
◯ 1688
◯ 1690
◯ 1691
◯ 1692
◯ 1693
◯ 1694
◯ 1695
◯ 1700
◯ 1701

- ◯ 1702
- ◯ 1703
- ◯ 1704
- ◯ 1705
- ◯ 1706
- ◯ 1707
- ◯ 1708
- ◯ 1709
- ◯ 1710
- ◯ 1711
- ◯ 1712
- ◯ 1713
- ◯ 1714
- ◯ 1717
- ◯ 1720
- ◯ 1721
- ◯ 1722
- ◯ 1723
- ◯ 1724
- ◯ 1725
- ◯ 1726
- ◯ 1727
- ◯ 1730
- ◯ 1731
- ◯ 1732
- ◯ 1733
- ◯ 1734
- ◯ 1735
- ◯ 1736
- ◯ 1737
- ◯ 1740
- ◯ 1750
- ◯ 1751
- ◯ 1752
- ◯ 1753
- ◯ 1754
- ◯ 1755
- ◯ 1756
- ◯ 1757
- ◯ 1758
- ◯ 1760
- ◯ 1761
- ◯ 1762
- ◯ 1769
- ◯ 1770
- ◯ 1772
- ◯ 1773
- ◯ 1774
- ◯ 1775
- ◯ 1776
- ◯ 1777
- ◯ 1780
- ◯ 1781
- ◯ 1782
- ◯ 1783
- ◯ 1784
- ◯ 1785
- ◯ 1786
- ◯ 1787
- ◯ 1788
- ◯ 1790
- ◯ 1792
- ◯ 1793
- ◯ 1794
- ◯ 1795
- ◯ 1796
- ◯ 1797
- ◯ 1798
- ◯ 1800
- ◯ 1801
- ◯ 1802

◯ 1803
◯ 1804
◯ 1805
◯ 1806
◯ 1807
◯ 1808
◯ 1809
◯ 1810
◯ 1819
◯ 1820
◯ 1821
◯ 1822
◯ 1823
◯ 1824
◯ 1825
◯ 1830
◯ 1832
◯ 1833
◯ 1834
◯ 1835
◯ 1836
◯ 1837
◯ 1840
◯ 1841
◯ 1842
◯ 1843
◯ 1844
◯ 1845
◯ 1846
◯ 1847
◯ 1850
◯ 1851
◯ 1854
◯ 1857
◯ 1860
◯ 1861
◯ 1862
◯ 1863
◯ 1864
◯ 1865
◯ 1866
◯ 1869
◯ 1870
◯ 1871
◯ 1876
◯ 1877
◯ 1880
◯ 1881
◯ 1882
◯ 1883
◯ 1884
◯ 1885
◯ 1890
◯ 1891
◯ 1892
◯ 1893
◯ 1894
◯ 1895
◯ 1896
◯ 1897
◯ 1898
◯ 1899
◯ 1900
◯ 1901
◯ 1902
◯ 1903
◯ 1904
◯ 1905
◯ 1906
◯ 1907
◯ 1908

◯ 1909
◯ 1910
◯ 1911
◯ 1912
◯ 1913
◯ 1914
◯ 1915
◯ 1916
◯ 1917
◯ 1918
◯ 1919
◯ 1924
◯ 1925
◯ 1930
◯ 1931
◯ 1932
◯ 1933
◯ 1934
◯ 1935
◯ 1936
◯ 1937
◯ 1938
◯ 1939
◯ 1940
◯ 1941
◯ 1942
◯ 1943
◯ 1944
◯ 1945
◯ 1946
◯ 1947
◯ 1948
◯ 1949
◯ 1950
◯ 1951
◯ 1952
◯ 1953
◯ 1954
◯ 1955
◯ 1956
◯ 1960
◯ 1961
◯ 1970
◯ 1971
◯ 1972
◯ 1973
◯ 1980
◯ 1981
◯ 1985
◯ 1988
◯ 1989
◯ 1990
◯ 1993
◯ 1994
◯ 1995
◯ 1996
◯ 1997
◯ 1998
◯ 2000
◯ 2001
◯ 2002
◯ 2003
◯ 2004
◯ 2005
◯ 2006
◯ 2007
◯ 2008
◯ 2009
◯ 2010
◯ 2011
◯ 2012

◯ 2013
◯ 2014
◯ 2015
◯ 2016
◯ 2017
◯ 2018
◯ 2019
◯ 2020
◯ 2021
◯ 2022
◯ 2023
◯ 2024
◯ 2025
◯ 2026
◯ 2027
◯ 2028
◯ 2029
◯ 2030
◯ 2031
◯ 2033
◯ 2034
◯ 2035
◯ 2037
◯ 2038
◯ 2039
◯ 2040
◯ 2041
◯ 2042
◯ 2043
◯ 2044
◯ 2045
◯ 2046
◯ 2047
◯ 2050
◯ 2051
◯ 2052
◯ 2053
◯ 2054
◯ 2055
◯ 2056
◯ 2057
◯ 2058
◯ 2059
◯ 2060
◯ 2061
◯ 2062
◯ 2063
◯ 2064
◯ 2065
◯ 2066
◯ 2067
◯ 2068
◯ 2069
◯ 2070
◯ 2071
◯ 2072
◯ 2073
◯ 2074
◯ 2075
◯ 2076
◯ 2077
◯ 2078
◯ 2079
◯ 2080
◯ 2081
◯ 2082
◯ 2083
◯ 2084
◯ 2085
◯ 2086
◯ 2087

◯ 2088
◯ 2089
◯ 2090
◯ 2091
◯ 2099
◯ 2100
◯ 2101
◯ 2102
◯ 2103
◯ 2104
◯ 2105
◯ 2106
◯ 2107
◯ 2108
◯ 2109
◯ 2110
◯ 2111
◯ 2112
◯ 2113
◯ 2114
◯ 2115
◯ 2116
◯ 2120
◯ 2121
◯ 2122
◯ 2123
◯ 2124
◯ 2125
◯ 2126
◯ 2127
◯ 2128
◯ 2129
◯ 2140
◯ 2141
◯ 2150
◯ 2152
◯ 2153
◯ 2154
◯ 2155
◯ 2156
◯ 2160
◯ 2161
◯ 2162
◯ 2163
◯ 2164
◯ 2165
◯ 2166
◯ 2167
◯ 2169
◯ 2170
◯ 2171
◯ 2172
◯ 2173
◯ 2174
◯ 2175
◯ 2176
◯ 2177
◯ 2178
◯ 2179
◯ 2180
◯ 2181
◯ 2182
◯ 2183
◯ 2184
◯ 2185
◯ 2186
◯ 2187
◯ 2189
◯ 2190
◯ 2191
◯ 2192

- ◯ 2193
- ◯ 2200
- ◯ 2201
- ◯ 2202
- ◯ 2203
- ◯ 2204
- ◯ 2206
- ◯ 2207
- ◯ 2208
- ◯ 2209
- ◯ 2210
- ◯ 2211
- ◯ 2212
- ◯ 2213
- ◯ 2215
- ◯ 2216
- ◯ 2217
- ◯ 2218
- ◯ 2219
- ◯ 2220
- ◯ 2221
- ◯ 2222
- ◯ 2223
- ◯ 2224
- ◯ 2230
- ◯ 2231
- ◯ 2232
- ◯ 2233
- ◯ 2235
- ◯ 2240
- ◯ 2241
- ◯ 2242
- ◯ 2243
- ◯ 2244
- ◯ 2245
- ◯ 2246
- ◯ 2247
- ◯ 2248
- ◯ 2250
- ◯ 2251
- ◯ 2252
- ◯ 2253
- ◯ 2254
- ◯ 2255
- ◯ 2256
- ◯ 2257
- ◯ 2258
- ◯ 2260
- ◯ 2261
- ◯ 2262
- ◯ 2263
- ◯ 2264
- ◯ 2265
- ◯ 2266
- ◯ 2270
- ◯ 2271
- ◯ 2272
- ◯ 2273
- ◯ 2274
- ◯ 2280
- ◯ 2281
- ◯ 2282
- ◯ 2283
- ◯ 2284
- ◯ 2285
- ◯ 2290
- ◯ 2291
- ◯ 2292
- ◯ 2293
- ◯ 2294
- ◯ 2295

○ 2296
○ 2297
○ 2298
○ 2300
○ 2301
○ 2302
○ 2303
○ 2304
○ 2305
○ 2306
○ 2307
○ 2308
○ 2309
○ 2310
○ 2311
○ 2312
○ 2313
○ 2314
○ 2315
○ 2316
○ 2317
○ 2318
○ 2319
○ 2321
○ 2322
○ 2323
○ 2324
○ 2325
○ 2326
○ 2327
○ 2328
○ 2329
○ 2330
○ 2331
○ 2332
○ 2333
○ 2334
○ 2335
○ 2338
○ 2339
○ 2340
○ 2341
○ 2342
○ 2343
○ 2344
○ 2345
○ 2346
○ 2347
○ 2348
○ 2350
○ 2351
○ 2352
○ 2354
○ 2355
○ 2360
○ 2361
○ 2362
○ 2363
○ 2365
○ 2366
○ 2367
○ 2368
○ 2369
○ 2370
○ 2380
○ 2382
○ 2383
○ 2384
○ 2385
○ 2386
○ 2387

◯ 2388
◯ 2389
◯ 2390
◯ 2391
◯ 2392
◯ 2393
◯ 2394
◯ 2395
◯ 2396
◯ 2397
◯ 2400
◯ 2401
◯ 2402
◯ 2403
◯ 2404
◯ 2405
◯ 2406
◯ 2407
◯ 2408
◯ 2409
◯ 2410
◯ 2411
◯ 2412
◯ 2413
◯ 2414
◯ 2415
◯ 2416
◯ 2417
◯ 2418
◯ 2420
◯ 2421
◯ 2422
◯ 2423
◯ 2424
◯ 2425
◯ 2426
◯ 2427
◯ 2428
◯ 2429
◯ 2430
◯ 2431
◯ 2432
◯ 2433
◯ 2434
◯ 2435
◯ 2436
◯ 2437
◯ 2438
◯ 2440
◯ 2441
◯ 2442
◯ 2443
◯ 2444
◯ 2445
◯ 2446
◯ 2447
◯ 2448
◯ 2450
◯ 2451
◯ 2452
◯ 2453
◯ 2454
◯ 2455
◯ 2456
◯ 2457
◯ 2458
◯ 2459
◯ 2460
◯ 2461
◯ 2462
◯ 2463

◯ 2464
◯ 2465
◯ 2470
◯ 2471
◯ 2472
◯ 2473
◯ 2474
◯ 2475
◯ 2480
◯ 2481
◯ 2482
◯ 2483
◯ 2484
◯ 2485
◯ 2486
◯ 2487
◯ 2488
◯ 2489
◯ 2490
◯ 2491
◯ 2492
◯ 2493
◯ 2494
◯ 2495
◯ 2496
◯ 2497
◯ 2498
◯ 2499
◯ 2500
◯ 2501
◯ 2502
◯ 2503
◯ 2504
◯ 2505
◯ 2506
◯ 2507
◯ 2508
◯ 2509
◯ 2510
◯ 2511
◯ 2512
◯ 2513
◯ 2514
◯ 2515
◯ 2516
◯ 2517
◯ 2518
◯ 2519
◯ 2520
◯ 2521
◯ 2523
◯ 2524
◯ 2525
◯ 2526
◯ 2527
◯ 2528
◯ 2530
◯ 2531
◯ 2532
◯ 2533
◯ 2535
◯ 2536
◯ 2537
◯ 2538
◯ 2539
◯ 2540
◯ 2541
◯ 2542
◯ 2543
◯ 2544
◯ 2550

- ◯ 2551
- ◯ 2552
- ◯ 2553
- ◯ 2554
- ◯ 2555
- ◯ 2556
- ◯ 2557
- ◯ 2560
- ◯ 2561
- ◯ 2562
- ◯ 2563
- ◯ 2564
- ◯ 2565
- ◯ 2566
- ◯ 2567
- ◯ 2568
- ◯ 2569
- ◯ 2570
- ◯ 2571
- ◯ 2572
- ◯ 2575
- ◯ 2577
- ◯ 2580
- ◯ 2581
- ◯ 2582
- ◯ 2583
- ◯ 2584
- ◯ 2585
- ◯ 2586
- ◯ 2587
- ◯ 2588
- ◯ 2590
- ◯ 2591
- ◯ 2592
- ◯ 2593
- ◯ 2594
- ◯ 2595
- ◯ 2596
- ◯ 2597
- ◯ 2598
- ◯ 2600
- ◯ 2603
- ◯ 2604
- ◯ 2605
- ◯ 2606
- ◯ 2607
- ◯ 2610
- ◯ 2612
- ◯ 2613
- ◯ 2614
- ◯ 2615
- ◯ 2616
- ◯ 2617
- ◯ 2618
- ◯ 2620
- ◯ 2621
- ◯ 2622
- ◯ 2623
- ◯ 2624
- ◯ 2625
- ◯ 2626
- ◯ 2627
- ◯ 2628
- ◯ 2629
- ◯ 2630
- ◯ 2632
- ◯ 2633
- ◯ 2634
- ◯ 2635
- ◯ 2636
- ◯ 2637

○ 2638
○ 2640
○ 2641
○ 2642
○ 2643
○ 2644
○ 2645
○ 2646
○ 2650
○ 2651
○ 2652
○ 2653
○ 2654
○ 2655
○ 2656
○ 2657
○ 2658
○ 2659
○ 2660
○ 2661
○ 2662
○ 2663
○ 2665
○ 2666
○ 2667
○ 2668
○ 2669
○ 2670
○ 2671
○ 2672
○ 2673
○ 2674
○ 2675
○ 2676
○ 2680
○ 2681
○ 2682
○ 2683
○ 2684
○ 2685
○ 2686
○ 2688
○ 2700
○ 2701
○ 2702
○ 2703
○ 2704
○ 2705
○ 2709
○ 2710
○ 2711
○ 2712
○ 2713
○ 2715
○ 2719
○ 2720
○ 2721
○ 2722
○ 2723
○ 2724
○ 2725
○ 2726
○ 2727
○ 2728
○ 2729
○ 2730
○ 2731
○ 2732
○ 2733
○ 2734
○ 2735

- ◯ 2736
- ◯ 2737
- ◯ 2740
- ◯ 2741
- ◯ 2742
- ◯ 2743
- ◯ 2745
- ◯ 2749
- ◯ 2750
- ◯ 2751
- ◯ 2752
- ◯ 2753
- ◯ 2754
- ◯ 2755
- ◯ 2756
- ◯ 2757
- ◯ 2758
- ◯ 2759
- ◯ 2760
- ◯ 2761
- ◯ 2762
- ◯ 2763
- ◯ 2764
- ◯ 2765
- ◯ 2766
- ◯ 2767
- ◯ 2769
- ◯ 2770
- ◯ 2771
- ◯ 2772
- ◯ 2780
- ◯ 2781
- ◯ 2782
- ◯ 2783
- ◯ 2784
- ◯ 2785
- ◯ 2786
- ◯ 2787
- ◯ 2788
- ◯ 2789
- ◯ 2790
- ◯ 2791
- ◯ 2792
- ◯ 2793
- ◯ 2794
- ◯ 2795
- ◯ 2796
- ◯ 2797
- ◯ 2798
- ◯ 2800
- ◯ 2801
- ◯ 2802
- ◯ 2803
- ◯ 2804
- ◯ 2805
- ◯ 2807
- ◯ 2808
- ◯ 2809
- ◯ 2810
- ◯ 2811
- ◯ 2812
- ◯ 2813
- ◯ 2814
- ◯ 2815
- ◯ 2816
- ◯ 2817
- ◯ 2820
- ◯ 2821
- ◯ 2822
- ◯ 2823
- ◯ 2824

- ◯ 2825
- ◯ 2826
- ◯ 2827
- ◯ 2828
- ◯ 2829
- ◯ 2830
- ◯ 2831
- ◯ 2832
- ◯ 2833
- ◯ 2834
- ◯ 2835
- ◯ 2836
- ◯ 2837
- ◯ 2838
- ◯ 2839
- ◯ 2840
- ◯ 2841
- ◯ 2842
- ◯ 2843
- ◯ 2844
- ◯ 2845
- ◯ 2846
- ◯ 2847
- ◯ 2848
- ◯ 2850
- ◯ 2851
- ◯ 2852
- ◯ 2853
- ◯ 2854
- ◯ 2855
- ◯ 2856
- ◯ 2857
- ◯ 2858
- ◯ 2859
- ◯ 2860
- ◯ 2861
- ◯ 2862
- ◯ 2863
- ◯ 2864
- ◯ 2865
- ◯ 2866
- ◯ 2867
- ◯ 2868
- ◯ 2869
- ◯ 2870
- ◯ 2871
- ◯ 2872
- ◯ 2873
- ◯ 2874
- ◯ 2875
- ◯ 2876
- ◯ 2877
- ◯ 2878
- ◯ 2879
- ◯ 2880
- ◯ 2881
- ◯ 2890
- ◯ 2900
- ◯ 2901
- ◯ 2902
- ◯ 2903
- ◯ 2904
- ◯ 2905
- ◯ 2906
- ◯ 2907
- ◯ 2908
- ◯ 2910
- ◯ 2911
- ◯ 2912
- ◯ 2913
- ◯ 2914

◯ 2915
◯ 2916
◯ 2917
◯ 2918
◯ 2920
◯ 2921
◯ 2922
◯ 2923
◯ 2924
◯ 2925
◯ 2926
◯ 2929
◯ 2930
◯ 2931
◯ 2932
◯ 2933
◯ 2934
◯ 2935
◯ 2936
◯ 2937
◯ 2938
◯ 2939
◯ 2940
◯ 2941
◯ 2942
◯ 2943
◯ 2944
◯ 2945
◯ 2946
◯ 2947
◯ 2948
◯ 2949
◯ 2950
◯ 2951
◯ 2952
◯ 2953
◯ 2954
◯ 2955
◯ 2956
◯ 2957
◯ 2958
◯ 2959
◯ 2960
◯ 2961
◯ 2962
◯ 2963
◯ 2964
◯ 2965
◯ 2966
◯ 2967
◯ 2968
◯ 2969
◯ 2970
◯ 2971
◯ 2972
◯ 2973
◯ 2974
◯ 2980
◯ 2981
◯ 2982
◯ 2983
◯ 2984
◯ 2985
◯ 2986
◯ 2989
◯ 2990
◯ 2991
◯ 2992
◯ 2993
◯ 2994
◯ 3000

◯ 3001
◯ 3002
◯ 3003
◯ 3004
◯ 3005
◯ 3006
◯ 3007
◯ 3008
◯ 3009
◯ 3010
◯ 3011
◯ 3012
◯ 3013
◯ 3014
◯ 3015
◯ 3016
◯ 3017
◯ 3018
◯ 3020
◯ 3021
◯ 3022
◯ 3023
◯ 3024
◯ 3025
◯ 3026
◯ 3027
◯ 3028
◯ 3029
◯ 3030
◯ 3031
◯ 3032
◯ 3033
◯ 3034
◯ 3035
◯ 3036
◯ 3037
◯ 3038
◯ 3039
◯ 3040
◯ 3041
◯ 3042
◯ 3043
◯ 3044
◯ 3045
◯ 3046
◯ 3047
◯ 3049
◯ 3050
◯ 3051
◯ 3052
◯ 3053
◯ 3054
◯ 3055
◯ 3056
◯ 3057
◯ 3058
◯ 3059
◯ 3060
◯ 3061
◯ 3062
◯ 3063
◯ 3064
◯ 3065
◯ 3066
◯ 3067
◯ 3068
◯ 3070
◯ 3071
◯ 3072
◯ 3073
◯ 3074

○ 3075
○ 3080
○ 3081
○ 3082
○ 3083
○ 3090
○ 3091
○ 3099
○ 3100
○ 3101
○ 3102
○ 3103
○ 3104
○ 3105
○ 3106
○ 3107
○ 3108
○ 3109
○ 3110
○ 3111
○ 3112
○ 3113
○ 3114
○ 3115
○ 3116
○ 3119
○ 3120
○ 3121
○ 3122
○ 3129
○ 3130
○ 3131
○ 3132
○ 3133
○ 3140
○ 3141
○ 3142
○ 3150
○ 3151
○ 3152
○ 3153
○ 3154
○ 3155
○ 3156
○ 3159
○ 3160
○ 3162
○ 3163
○ 3164
○ 3165
○ 3169
○ 3170
○ 3171
○ 3172
○ 3173
○ 3174
○ 3175
○ 3176
○ 3177
○ 3178
○ 3179
○ 3180
○ 3181
○ 3182
○ 3183
○ 3190
○ 3191
○ 3199
○ 3200
○ 3201
○ 3202

- ◯ 3203
- ◯ 3204
- ◯ 3205
- ◯ 3206
- ◯ 3207
- ◯ 3208
- ◯ 3209
- ◯ 3210
- ◯ 3211
- ◯ 3212
- ◯ 3213
- ◯ 3214
- ◯ 3215
- ◯ 3216
- ◯ 3217
- ◯ 3218
- ◯ 3219
- ◯ 3220
- ◯ 3221
- ◯ 3222
- ◯ 3223
- ◯ 3224
- ◯ 3225
- ◯ 3226
- ◯ 3227
- ◯ 3230
- ◯ 3231
- ◯ 3232
- ◯ 3233
- ◯ 3234
- ◯ 3235
- ◯ 3236
- ◯ 3239
- ◯ 3240
- ◯ 3241
- ◯ 3242
- ◯ 3243
- ◯ 3244
- ◯ 3245
- ◯ 3246
- ◯ 3250
- ◯ 3251
- ◯ 3252
- ◯ 3253
- ◯ 3254
- ◯ 3255
- ◯ 3256
- ◯ 3257
- ◯ 3258
- ◯ 3259
- ◯ 3260
- ◯ 3261
- ◯ 3262
- ◯ 3263
- ◯ 3264
- ◯ 3265
- ◯ 3266
- ◯ 3268
- ◯ 3269
- ◯ 3270
- ◯ 3271
- ◯ 3272
- ◯ 3273
- ◯ 3274
- ◯ 3275
- ◯ 3276
- ◯ 3277
- ◯ 3278
- ◯ 3279
- ◯ 3280
- ◯ 3281

◯ 3282
◯ 3283
◯ 3285
◯ 3286
◯ 3287
◯ 3288
◯ 3289
◯ 3290
◯ 3291
◯ 3292
◯ 3293
◯ 3294
◯ 3295
◯ 3296
◯ 3297
◯ 3300
◯ 3301
◯ 3302
◯ 3303
◯ 3304
◯ 3305
◯ 3306
◯ 3307
◯ 3308
◯ 3309
◯ 3310
◯ 3311
◯ 3312
◯ 3313
◯ 3314
◯ 3315
◯ 3316
◯ 3317
◯ 3318
◯ 3319
◯ 3320
◯ 3322
◯ 3323
◯ 3324
◯ 3325
◯ 3326
◯ 3328
◯ 3329
◯ 3330
◯ 3331
◯ 3332
◯ 3333
◯ 3334
◯ 3335
◯ 3338
◯ 3339
◯ 3340
◯ 3341
◯ 3342
◯ 3343
◯ 3344
◯ 3345
◯ 3346
◯ 3347
◯ 3348
◯ 3349
◯ 3350
◯ 3351
◯ 3352
◯ 3353
◯ 3354
◯ 3355
◯ 3356
◯ 3357
◯ 3358
◯ 3359

○ 3360
○ 3361
○ 3362
○ 3363
○ 3364
○ 3365
○ 3366
○ 3367
○ 3368
○ 3369
○ 3370
○ 3371
○ 3372
○ 3373
○ 3374
○ 3375
○ 3376
○ 3377
○ 3378
○ 3380
○ 3381
○ 3382
○ 3383
○ 3384
○ 3385
○ 3386
○ 3387
○ 3388
○ 3389
○ 3390
○ 3391
○ 3392
○ 3393
○ 3394
○ 3395
○ 3396
○ 3397
○ 3398
○ 3399
○ 3400
○ 3401
○ 3402
○ 3403
○ 3404
○ 3405
○ 3406
○ 3407
○ 3408
○ 3409
○ 3410
○ 3411
○ 3412
○ 3413
○ 3414
○ 3420
○ 3421
○ 3422
○ 3423
○ 3424
○ 3425
○ 3426
○ 3427
○ 3428
○ 3429
○ 3442
○ 3443
○ 3444
○ 3445
○ 3446
○ 3447
○ 3448

◯ 3449
◯ 3460
◯ 3461
◯ 3463
◯ 3465
◯ 3466
◯ 3467
◯ 3468
◯ 3469
◯ 3470
◯ 3471
◯ 3472
◯ 3473
◯ 3474
◯ 3475
◯ 3476
◯ 3477
◯ 3478
◯ 3479
◯ 3494
◯ 3495
◯ 3497
◯ 3498
◯ 3499
◯ 3500
◯ 3501
◯ 3502
◯ 3503
◯ 3504
◯ 3505
◯ 3506
◯ 3507
◯ 3508
◯ 3509
◯ 3511
◯ 3512
◯ 3513
◯ 3514
◯ 3515
◯ 3516
◯ 3517
◯ 3518
◯ 3520
◯ 3521
◯ 3522
◯ 3523
◯ 3524
◯ 3525
◯ 3526
◯ 3527
◯ 3528
◯ 3529
◯ 3540
◯ 3544
◯ 3545
◯ 3546
◯ 3547
◯ 3548
◯ 3549
◯ 3550
◯ 3554
◯ 3555
◯ 3556
◯ 3557
◯ 3558
◯ 3559
◯ 3560
◯ 3561
◯ 3562
◯ 3563
◯ 3564

◯ 3565
◯ 3566
◯ 3567
◯ 3569
◯ 3573
◯ 3574
◯ 3575
◯ 3576
◯ 3577
◯ 3580
◯ 3581
◯ 3582
◯ 3589
◯ 3590
◯ 3595
◯ 3596
◯ 3597
◯ 3598
◯ 3599
◯ 3600
◯ 3601
◯ 3602
◯ 3603
◯ 3604
◯ 3605
◯ 3606
◯ 3607
◯ 3608
◯ 3609
◯ 3610
◯ 3611
◯ 3612
◯ 3613
◯ 3614
◯ 3617
◯ 3619
◯ 3620
◯ 3625
◯ 3626
◯ 3627
◯ 3628
◯ 3630
◯ 3631
◯ 3632
◯ 3633
◯ 3634
◯ 3635
◯ 3636
◯ 3637
◯ 3640
◯ 3642
◯ 3643
◯ 3644
◯ 3645
◯ 3646
◯ 3647
◯ 3648
◯ 3650
◯ 3651
◯ 3652
◯ 3653
◯ 3654
◯ 3655
◯ 3656
◯ 3657
◯ 3658
◯ 3659
◯ 3660
◯ 3661
◯ 3662
◯ 3663

- ◯ 3664
- ◯ 3665
- ◯ 3666
- ◯ 3667
- ◯ 3668
- ◯ 3669
- ◯ 3670
- ◯ 3672
- ◯ 3673
- ◯ 3674
- ◯ 3675
- ◯ 3676
- ◯ 3677
- ◯ 3678
- ◯ 3679
- ◯ 3680
- ◯ 3683
- ◯ 3684
- ◯ 3685
- ◯ 3686
- ◯ 3687
- ◯ 3690
- ◯ 3691
- ◯ 3692
- ◯ 3701
- ◯ 3702
- ◯ 3703
- ◯ 3704
- ◯ 3705
- ◯ 3706
- ◯ 3707
- ◯ 3708
- ◯ 3709
- ◯ 3710
- ◯ 3711
- ◯ 3712
- ◯ 3713
- ◯ 3714
- ◯ 3715
- ◯ 3716
- ◯ 3717
- ◯ 3718
- ◯ 3719
- ◯ 3720
- ◯ 3721
- ◯ 3722
- ◯ 3723
- ◯ 3724
- ◯ 3725
- ◯ 3730
- ◯ 3731
- ◯ 3732
- ◯ 3733
- ◯ 3734
- ◯ 3735
- ◯ 3736
- ◯ 3737
- ◯ 3738
- ◯ 3739
- ◯ 3740
- ◯ 3741
- ◯ 3742
- ◯ 3745
- ◯ 3750
- ◯ 3754
- ◯ 3760
- ◯ 3761
- ◯ 3762
- ◯ 3764
- ◯ 3765
- ◯ 3766

○ 3768
○ 3769
○ 3770
○ 3771
○ 3772
○ 3773
○ 3774
○ 3775
○ 3776
○ 3777
○ 3780
○ 3781
○ 3782
○ 3783
○ 3784
○ 3785
○ 3786
○ 3787
○ 3788
○ 3789
○ 3790
○ 3791
○ 3792
○ 3793
○ 3794
○ 3795
○ 3799
○ 3800
○ 3801
○ 3802
○ 3803
○ 3804
○ 3805
○ 3806
○ 3807
○ 3808
○ 3810
○ 3811
○ 3812
○ 3813
○ 3814
○ 3815
○ 3816
○ 3817
○ 3818
○ 3819
○ 3820
○ 3822
○ 3823
○ 3824
○ 3825
○ 3826
○ 3827
○ 3828
○ 3830
○ 3831
○ 3832
○ 3833
○ 3834
○ 3835
○ 3836
○ 3837
○ 3838
○ 3839
○ 3840
○ 3842
○ 3844
○ 3845
○ 3846
○ 3847
○ 3848

- ◯ 3850
- ◯ 3854
- ◯ 3855
- ◯ 3856
- ◯ 3857
- ◯ 3858
- ◯ 3860
- ◯ 3861
- ◯ 3862
- ◯ 3863
- ◯ 3864
- ◯ 3865
- ◯ 3866
- ◯ 3867
- ◯ 3868
- ◯ 3870
- ◯ 3872
- ◯ 3873
- ◯ 3874
- ◯ 3875
- ◯ 3876
- ◯ 3877
- ◯ 3878
- ◯ 3880
- ◯ 3882
- ◯ 3883
- ◯ 3884
- ◯ 3885
- ◯ 3886
- ◯ 3887
- ◯ 3888
- ◯ 3890
- ◯ 3891
- ◯ 3892
- ◯ 3893
- ◯ 3894
- ◯ 3895
- ◯ 3896
- ◯ 3903
- ◯ 3904
- ◯ 3905
- ◯ 3906
- ◯ 3907
- ◯ 3908
- ◯ 3909
- ◯ 3910
- ◯ 3911
- ◯ 3912
- ◯ 3913
- ◯ 3914
- ◯ 3915
- ◯ 3916
- ◯ 3917
- ◯ 3918
- ◯ 3919
- ◯ 3920
- ◯ 3921
- ◯ 3922
- ◯ 3923
- ◯ 3925
- ◯ 3926
- ◯ 3927
- ◯ 3928
- ◯ 3929
- ◯ 3930
- ◯ 3932
- ◯ 3933
- ◯ 3934
- ◯ 3935
- ◯ 3936
- ◯ 3940

- ◯ 3942
- ◯ 3943
- ◯ 3944
- ◯ 3945
- ◯ 3946
- ◯ 3947
- ◯ 3948
- ◯ 3950
- ◯ 3952
- ◯ 3953
- ◯ 3954
- ◯ 3955
- ◯ 3956
- ◯ 3957
- ◯ 3958
- ◯ 3959
- ◯ 3960
- ◯ 3962
- ◯ 3963
- ◯ 3964
- ◯ 3965
- ◯ 3966
- ◯ 3970
- ◯ 3971
- ◯ 3973
- ◯ 3974
- ◯ 3975
- ◯ 3976
- ◯ 3977
- ◯ 3981
- ◯ 3982
- ◯ 3983
- ◯ 3984
- ◯ 3985
- ◯ 3986
- ◯ 3987
- ◯ 3988
- ◯ 3989
- ◯ 3990
- ◯ 4000
- ◯ 4001
- ◯ 4002
- ◯ 4003
- ◯ 4004
- ◯ 4005
- ◯ 4006
- ◯ 4007
- ◯ 4010
- ◯ 4011
- ◯ 4012
- ◯ 4014
- ◯ 4015
- ◯ 4016
- ◯ 4017
- ◯ 4020
- ◯ 4021
- ◯ 4022
- ◯ 4023
- ◯ 4024
- ◯ 4025
- ◯ 4026
- ◯ 4027
- ◯ 4028
- ◯ 4029
- ◯ 4031
- ◯ 4032
- ◯ 4033
- ◯ 4034
- ◯ 4035
- ◯ 4036
- ◯ 4037

◯ 4038
◯ 4039
◯ 4040
◯ 4041
◯ 4042
◯ 4043
◯ 4044
◯ 4045
◯ 4046
◯ 4047
◯ 4048
◯ 4049
◯ 4050
◯ 4051
◯ 4052
◯ 4053
◯ 4054
◯ 4055
◯ 4057
◯ 4058
◯ 4059
◯ 4060
◯ 4061
◯ 4062
◯ 4070
◯ 4072
◯ 4073
◯ 4074
◯ 4075
◯ 4076
◯ 4077
◯ 4080
◯ 4081
◯ 4082
◯ 4083
◯ 4084
◯ 4085
◯ 4086
◯ 4087
◯ 4090
◯ 4091
◯ 4092
◯ 4093
◯ 4094
◯ 4095
◯ 4096
◯ 4097
◯ 4098
◯ 4099
◯ 4100
◯ 4101
◯ 4102
◯ 4103
◯ 4104
◯ 4105
◯ 4106
◯ 4107
◯ 4108
◯ 4109
◯ 4110
◯ 4111
◯ 4112
◯ 4113
◯ 4114
◯ 4115
◯ 4116
◯ 4117
◯ 4118
◯ 4120
◯ 4121
◯ 4122

◯ 4123
◯ 4124
◯ 4125
◯ 4126
◯ 4127
◯ 4130
◯ 4131
◯ 4133
◯ 4134
◯ 4135
◯ 4136
◯ 4138
◯ 4139
◯ 4140
◯ 4141
◯ 4142
◯ 4145
◯ 4146
◯ 4147
◯ 4150
◯ 4151
◯ 4152
◯ 4153
◯ 4154
◯ 4155
◯ 4156
◯ 4157
◯ 4160
◯ 4161
◯ 4162
◯ 4163
◯ 4164
◯ 4165
◯ 4166
◯ 4170
◯ 4171
◯ 4172
◯ 4173
◯ 4174
◯ 4175
◯ 4176
◯ 4177
◯ 4180
◯ 4181
◯ 4182
◯ 4183
◯ 4184
◯ 4185
◯ 4186
◯ 4200
◯ 4202
◯ 4203
◯ 4204
◯ 4205
◯ 4206
◯ 4207
◯ 4208
◯ 4210
◯ 4212
◯ 4213
◯ 4214
◯ 4215
◯ 4216
◯ 4217
◯ 4220
◯ 4221
◯ 4222
◯ 4223
◯ 4224
◯ 4225
◯ 4226

◯ 4227
◯ 4228
◯ 4230
◯ 4232
◯ 4233
◯ 4234
◯ 4235
◯ 4236
◯ 4237
◯ 4240
◯ 4241
◯ 4242
◯ 4243
◯ 4244
◯ 4245
◯ 4246
◯ 4250
◯ 4251
◯ 4252
◯ 4253
◯ 4254
◯ 4255
◯ 4256
◯ 4260
◯ 4262
◯ 4263
◯ 4264
◯ 4265
◯ 4270
◯ 4271
◯ 4272
◯ 4273
◯ 4274
◯ 4275
◯ 4276
◯ 4277
◯ 4278
◯ 4300
◯ 4301
◯ 4302
◯ 4303
◯ 4304
◯ 4305
◯ 4306
◯ 4307
◯ 4308
◯ 4309
◯ 4310
◯ 4311
◯ 4312
◯ 4313
◯ 4314
◯ 4315
◯ 4316
◯ 4319
◯ 4320
◯ 4321
◯ 4322
◯ 4323
◯ 4324
◯ 4325
◯ 4326
◯ 4327
◯ 4328
◯ 4329
◯ 4330
◯ 4331
◯ 4332
◯ 4333
◯ 4334
◯ 4335

○ 4336
○ 4340
○ 4341
○ 4342
○ 4343
○ 4344
○ 4345
○ 4346
○ 4350
○ 4351
○ 4352
○ 4353
○ 4354
○ 4355
○ 4356
○ 4357
○ 4360
○ 4361
○ 4362
○ 4363
○ 4365
○ 4366
○ 4368
○ 4369
○ 4370
○ 4371
○ 4372
○ 4373
○ 4374
○ 4375
○ 4376
○ 4377
○ 4378
○ 4379
○ 4380
○ 4381
○ 4382
○ 4383
○ 4384
○ 4390
○ 4391
○ 4392
○ 4393
○ 4394
○ 4395
○ 4396
○ 4397
○ 4398
○ 4400
○ 4401
○ 4402
○ 4403
○ 4404
○ 4405
○ 4406
○ 4407
○ 4408
○ 4409
○ 4410
○ 4411
○ 4412
○ 4413
○ 4414
○ 4418
○ 4419
○ 4420
○ 4421
○ 4422
○ 4423
○ 4424
○ 4425

- ◯ 4426
- ◯ 4427
- ◯ 4428
- ◯ 4430
- ◯ 4431
- ◯ 4432
- ◯ 4433
- ◯ 4437
- ◯ 4439
- ◯ 4440
- ◯ 4441
- ◯ 4442
- ◯ 4443
- ◯ 4444
- ◯ 4445
- ◯ 4446
- ◯ 4447
- ◯ 4448
- ◯ 4449
- ◯ 4450
- ◯ 4451
- ◯ 4455
- ◯ 4460
- ◯ 4461
- ◯ 4462
- ◯ 4463
- ◯ 4464
- ◯ 4465
- ◯ 4466
- ◯ 4467
- ◯ 4468
- ◯ 4469
- ◯ 4470
- ◯ 4471
- ◯ 4472
- ◯ 4473
- ◯ 4475
- ◯ 4476
- ◯ 4479
- ◯ 4480
- ◯ 4481
- ◯ 4482
- ◯ 4483
- ◯ 4484
- ◯ 4485
- ◯ 4486
- ◯ 4487
- ◯ 4488
- ◯ 4489
- ◯ 4490
- ◯ 4500
- ◯ 4501
- ◯ 4503
- ◯ 4504
- ◯ 4505
- ◯ 4506
- ◯ 4507
- ◯ 4510
- ◯ 4511
- ◯ 4512
- ◯ 4513
- ◯ 4514
- ◯ 4515
- ◯ 4516
- ◯ 4517
- ◯ 4520
- ◯ 4521
- ◯ 4522
- ◯ 4523
- ◯ 4524
- ◯ 4525

○ 4526
○ 4527
○ 4528
○ 4529
○ 4530
○ 4531
○ 4532
○ 4533
○ 4534
○ 4535
○ 4536
○ 4537
○ 4538
○ 4539
○ 4540
○ 4541
○ 4542
○ 4543
○ 4544
○ 4545
○ 4546
○ 4547
○ 4548
○ 4549
○ 4550
○ 4560
○ 4561
○ 4562
○ 4563
○ 4564
○ 4565
○ 4566
○ 4567
○ 4568
○ 4569
○ 4570
○ 4571
○ 4572
○ 4573
○ 4574
○ 4575
○ 4576
○ 4577
○ 4578
○ 4580
○ 4581
○ 4582
○ 4583
○ 4584
○ 4585
○ 4586
○ 4587
○ 4588
○ 4589
○ 4599
○ 4600
○ 4601
○ 4603
○ 4604
○ 4605
○ 4606
○ 4607
○ 4608
○ 4610
○ 4611
○ 4612
○ 4613
○ 4614
○ 4615
○ 4616
○ 4617

- ◯ 4618
- ◯ 4620
- ◯ 4621
- ◯ 4622
- ◯ 4623
- ◯ 4624
- ◯ 4625
- ◯ 4626
- ◯ 4627
- ◯ 4628
- ◯ 4629
- ◯ 4630
- ◯ 4631
- ◯ 4632
- ◯ 4634
- ◯ 4635
- ◯ 4636
- ◯ 4637
- ◯ 4638
- ◯ 4639
- ◯ 4640
- ◯ 4641
- ◯ 4650
- ◯ 4651
- ◯ 4652
- ◯ 4653
- ◯ 4654
- ◯ 4655
- ◯ 4656
- ◯ 4657
- ◯ 4658
- ◯ 4659
- ◯ 4660
- ◯ 4661
- ◯ 4662
- ◯ 4663
- ◯ 4666
- ◯ 4668
- ◯ 4669
- ◯ 4670
- ◯ 4671
- ◯ 4672
- ◯ 4673
- ◯ 4674
- ◯ 4675
- ◯ 4676
- ◯ 4677
- ◯ 4678
- ◯ 4679
- ◯ 4680
- ◯ 4681
- ◯ 4682
- ◯ 4683
- ◯ 4684
- ◯ 4685
- ◯ 4686
- ◯ 4688
- ◯ 4689
- ◯ 4690
- ◯ 4691
- ◯ 4692
- ◯ 4693
- ◯ 4694
- ◯ 4695
- ◯ 4696
- ◯ 4697
- ◯ 4698
- ◯ 4699
- ◯ 4700
- ◯ 4701
- ◯ 4702

- ◯ 4703
- ◯ 4704
- ◯ 4706
- ◯ 4710
- ◯ 4711
- ◯ 4712
- ◯ 4713
- ◯ 4714
- ◯ 4715
- ◯ 4716
- ◯ 4717
- ◯ 4719
- ◯ 4720
- ◯ 4722
- ◯ 4723
- ◯ 4724
- ◯ 4725
- ◯ 4726
- ◯ 4727
- ◯ 4728
- ◯ 4730
- ◯ 4732
- ◯ 4733
- ◯ 4734
- ◯ 4735
- ◯ 4736
- ◯ 4737
- ◯ 4738
- ◯ 4739
- ◯ 4740
- ◯ 4742
- ◯ 4743
- ◯ 4744
- ◯ 4745
- ◯ 4746
- ◯ 4747
- ◯ 4750
- ◯ 4751
- ◯ 4752
- ◯ 4753
- ◯ 4754
- ◯ 4755
- ◯ 4756
- ◯ 4757
- ◯ 4758
- ◯ 4759
- ◯ 4760
- ◯ 4761
- ◯ 4762
- ◯ 4763
- ◯ 4764
- ◯ 4765
- ◯ 4766
- ◯ 4767
- ◯ 4768
- ◯ 4770
- ◯ 4771
- ◯ 4772
- ◯ 4773
- ◯ 4774
- ◯ 4775
- ◯ 4780
- ◯ 4783
- ◯ 4784
- ◯ 4785
- ◯ 4786
- ◯ 4787
- ◯ 4788
- ◯ 4790
- ◯ 4791
- ◯ 4792

◯ 4793
◯ 4794
◯ 4795
◯ 4796
◯ 4797
◯ 4798
◯ 4799
◯ 4800
◯ 4801
◯ 4802
◯ 4803
◯ 4804
◯ 4805
◯ 4806
◯ 4807
◯ 4808
◯ 4809
◯ 4810
◯ 4811
◯ 4812
◯ 4813
◯ 4814
◯ 4815
◯ 4816
◯ 4817
◯ 4818
◯ 4819
◯ 4820
◯ 4821
◯ 4822
◯ 4823
◯ 4824
◯ 4825
◯ 4826
◯ 4827
◯ 4828
◯ 4830
◯ 4831
◯ 4832
◯ 4833
◯ 4834
◯ 4835
◯ 4836
◯ 4837
◯ 4838
◯ 4839
◯ 4840
◯ 4841
◯ 4842
◯ 4843
◯ 4844
◯ 4845
◯ 4846
◯ 4847
◯ 4848
◯ 4850
◯ 4851
◯ 4852
◯ 4853
◯ 4855
◯ 4860
◯ 4861
◯ 4862
◯ 4863
◯ 4864
◯ 4865
◯ 4866
◯ 4867
◯ 4868
◯ 4870
◯ 4871

◯ 4872
◯ 4873
◯ 4874
◯ 4875
◯ 4876
◯ 4877
◯ 4878
◯ 4880
◯ 4881
◯ 4882
◯ 4883
◯ 4884
◯ 4885
◯ 4886
◯ 4887
◯ 4888
◯ 4889
◯ 4890
◯ 4891
◯ 4892
◯ 4893
◯ 4895
◯ 4898
◯ 4900
◯ 4901
◯ 4902
◯ 4903
◯ 4904
◯ 4905
◯ 4906
◯ 4907
◯ 4908
◯ 4909
◯ 4910
◯ 4911
◯ 4912
◯ 4913
◯ 4920
◯ 4922
◯ 4923
◯ 4924
◯ 4925
◯ 4926
◯ 4927
◯ 4928
◯ 4930
◯ 4931
◯ 4932
◯ 4933
◯ 4934
◯ 4935
◯ 4940
◯ 4941
◯ 4942
◯ 4943
◯ 4944
◯ 4945
◯ 4946
◯ 4950
◯ 4951
◯ 4952
◯ 4953
◯ 4954
◯ 4955
◯ 4956
◯ 4958
◯ 4959
◯ 4960
◯ 4961
◯ 4962
◯ 4963

- ◯ 4964
- ◯ 4965
- ◯ 4966
- ◯ 4967
- ◯ 4968
- ◯ 4969
- ◯ 4970
- ◯ 4971
- ◯ 4972
- ◯ 4973
- ◯ 4974
- ◯ 4975
- ◯ 4976
- ◯ 4977
- ◯ 4978
- ◯ 4979
- ◯ 4980
- ◯ 4981
- ◯ 4982
- ◯ 4983
- ◯ 4984
- ◯ 4985
- ◯ 4986
- ◯ 4987
- ◯ 4988
- ◯ 4989
- ◯ 4990
- ◯ 4991
- ◯ 4992
- ◯ 4993
- ◯ 4994
- ◯ 4995
- ◯ 4996
- ◯ 4997
- ◯ 5000
- ◯ 5001
- ◯ 5002
- ◯ 5003
- ◯ 5004
- ◯ 5005
- ◯ 5006
- ◯ 5007
- ◯ 5009
- ◯ 5010
- ◯ 5011
- ◯ 5012
- ◯ 5013
- ◯ 5014
- ◯ 5015
- ◯ 5016
- ◯ 5017
- ◯ 5020
- ◯ 5021
- ◯ 5022
- ◯ 5023
- ◯ 5024
- ◯ 5025
- ◯ 5026
- ◯ 5027
- ◯ 5030
- ◯ 5031
- ◯ 5032
- ◯ 5033
- ◯ 5034
- ◯ 5035
- ◯ 5036
- ◯ 5038
- ◯ 5039
- ◯ 5040
- ◯ 5042
- ◯ 5043

◯ 5044
◯ 5045
◯ 5046
◯ 5047
◯ 5048
◯ 5050
◯ 5051
◯ 5052
◯ 5053
◯ 5054
◯ 5055
◯ 5056
◯ 5057
◯ 5058
◯ 5059
◯ 5060
◯ 5061
◯ 5062
◯ 5063
◯ 5064
◯ 5065
◯ 5066
◯ 5067
◯ 5068
◯ 5070
◯ 5080
◯ 5083
◯ 5084
◯ 5085
◯ 5086
◯ 5093
◯ 5094
◯ 5095
◯ 5098
◯ 5100
◯ 5101
◯ 5102
◯ 5103
◯ 5104
◯ 5105
◯ 5106
◯ 5110
◯ 5111
◯ 5120
◯ 5123
◯ 5124
◯ 5125
◯ 5130
◯ 5133
◯ 5134
◯ 5135
◯ 5136
◯ 5140
◯ 5143
◯ 5144
◯ 5145
◯ 5146
◯ 5150
◯ 5151
◯ 5152
◯ 5153
◯ 5154
◯ 5155
◯ 5156
◯ 5157
◯ 5159
◯ 5160
◯ 5163
◯ 5164
◯ 5165
◯ 5200

- ◯ 5203
- ◯ 5204
- ◯ 5205
- ◯ 5206
- ◯ 5207
- ◯ 5209
- ◯ 5210
- ◯ 5213
- ◯ 5214
- ◯ 5215
- ◯ 5216
- ◯ 5217
- ◯ 5220
- ◯ 5221
- ◯ 5222
- ◯ 5223
- ◯ 5224
- ◯ 5225
- ◯ 5230
- ◯ 5231
- ◯ 5232
- ◯ 5233
- ◯ 5234
- ◯ 5235
- ◯ 5236
- ◯ 5240
- ◯ 5241
- ◯ 5249
- ◯ 5250
- ◯ 5253
- ◯ 5254
- ◯ 5255
- ◯ 5256
- ◯ 5257
- ◯ 5258
- ◯ 5259
- ◯ 5260
- ◯ 5261
- ◯ 5262
- ◯ 5263
- ◯ 5264
- ◯ 5265
- ◯ 5266
- ◯ 5270
- ◯ 5272
- ◯ 5273
- ◯ 5274
- ◯ 5275
- ◯ 5276
- ◯ 5277
- ◯ 5280
- ◯ 5300
- ◯ 5301
- ◯ 5302
- ◯ 5303
- ◯ 5304
- ◯ 5305
- ◯ 5306
- ◯ 5307
- ◯ 5308
- ◯ 5309
- ◯ 5310
- ◯ 5311
- ◯ 5312
- ◯ 5313
- ◯ 5314
- ◯ 5315
- ◯ 5316
- ◯ 5317
- ◯ 5318
- ◯ 5319

- ◯ 5320
- ◯ 5321
- ◯ 5322
- ◯ 5323
- ◯ 5325
- ◯ 5326
- ◯ 5327
- ◯ 5328
- ◯ 5329
- ◯ 5340
- ◯ 5350
- ◯ 5351
- ◯ 5352
- ◯ 5353
- ◯ 5354
- ◯ 5355
- ◯ 5356
- ◯ 5357
- ◯ 5358
- ◯ 5359
- ◯ 5370
- ◯ 5371
- ◯ 5372
- ◯ 5374
- ◯ 5377
- ◯ 5378
- ◯ 5379
- ◯ 5380
- ◯ 5381
- ◯ 5382
- ◯ 5390
- ◯ 5391
- ◯ 5392
- ◯ 5393
- ◯ 5394
- ◯ 5395
- ◯ 5396
- ◯ 5400
- ◯ 5401
- ◯ 5402
- ◯ 5408
- ◯ 5410
- ◯ 5411
- ◯ 5412
- ◯ 5413
- ◯ 5414
- ◯ 5415
- ◯ 5416
- ◯ 5417
- ◯ 5418
- ◯ 5420
- ◯ 5421
- ◯ 5422
- ◯ 5423
- ◯ 5424
- ◯ 5430
- ◯ 5431
- ◯ 5432
- ◯ 5434
- ◯ 5440
- ◯ 5441
- ◯ 5442
- ◯ 5443
- ◯ 5444
- ◯ 5445
- ◯ 5446
- ◯ 5447
- ◯ 5448
- ◯ 5449
- ◯ 5450
- ◯ 5451

- ◯ 5452
- ◯ 5453
- ◯ 5454
- ◯ 5455
- ◯ 5456
- ◯ 5460
- ◯ 5461
- ◯ 5462
- ◯ 5463
- ◯ 5464
- ◯ 5465
- ◯ 5466
- ◯ 5467
- ◯ 5470
- ◯ 5472
- ◯ 5473
- ◯ 5474
- ◯ 5475
- ◯ 5476
- ◯ 5477
- ◯ 5480
- ◯ 5481
- ◯ 5482
- ◯ 5483
- ◯ 5484
- ◯ 5485
- ◯ 5486
- ◯ 5487
- ◯ 5488
- ◯ 5489
- ◯ 5490
- ◯ 5491
- ◯ 5492
- ◯ 5493
- ◯ 5494
- ◯ 5495
- ◯ 5496
- ◯ 5497
- ◯ 5498
- ◯ 5500
- ◯ 5501
- ◯ 5502
- ◯ 5503
- ◯ 5504
- ◯ 5505
- ◯ 5506
- ◯ 5507
- ◯ 5508
- ◯ 5509
- ◯ 5510
- ◯ 5511
- ◯ 5512
- ◯ 5513
- ◯ 5514
- ◯ 5515
- ◯ 5516
- ◯ 5517
- ◯ 5530
- ◯ 5531
- ◯ 5532
- ◯ 5533
- ◯ 5534
- ◯ 5535
- ◯ 5536
- ◯ 5537
- ◯ 5538
- ◯ 5539
- ◯ 5540
- ◯ 5541
- ◯ 5542
- ◯ 5543

○ 5544
○ 5545
○ 5546
○ 5547
○ 5548
○ 5555
○ 5556
○ 5557
○ 5559
○ 5560
○ 5561
○ 5570
○ 5571
○ 5572
○ 5573
○ 5574
○ 5575
○ 5576
○ 5577
○ 5578
○ 5579
○ 5580
○ 5581
○ 5590
○ 5591
○ 5592
○ 5593
○ 5594
○ 5595
○ 5596
○ 5597
○ 5598
○ 5599
○ 5600
○ 5601
○ 5602
○ 5603
○ 5604
○ 5605
○ 5606
○ 5607
○ 5608
○ 5609
○ 5610
○ 5611
○ 5612
○ 5613
○ 5614
○ 5615
○ 5616
○ 5617
○ 5618
○ 5620
○ 5621
○ 5622
○ 5623
○ 5624
○ 5625
○ 5626
○ 5627
○ 5628
○ 5629
○ 5630
○ 5631
○ 5632
○ 5633
○ 5634
○ 5635
○ 5636
○ 5637
○ 5638

○ 5639
○ 5640
○ 5642
○ 5643
○ 5644
○ 5645
○ 5646
○ 5647
○ 5648
○ 5650
○ 5651
○ 5652
○ 5653
○ 5654
○ 5655
○ 5656
○ 5657
○ 5658
○ 5659
○ 5660
○ 5661
○ 5662
○ 5663
○ 5664
○ 5665
○ 5666
○ 5667
○ 5668
○ 5670
○ 5671
○ 5672
○ 5673
○ 5674
○ 5675
○ 5676
○ 5690
○ 5691
○ 5692
○ 5693
○ 5694
○ 5695
○ 5696
○ 5697
○ 5699
○ 5700
○ 5701
○ 5702
○ 5703
○ 5704
○ 5705
○ 5706
○ 5707
○ 5710
○ 5711
○ 5718
○ 5719
○ 5720
○ 5721
○ 5722
○ 5723
○ 5724
○ 5725
○ 5726
○ 5727
○ 5730
○ 5731
○ 5732
○ 5733
○ 5734
○ 5735
○ 5736

◯ 5737
◯ 5738
◯ 5739
◯ 5740
◯ 5742
◯ 5743
◯ 5744
◯ 5745
◯ 5746
◯ 5747
◯ 5748
◯ 5750
◯ 5752
◯ 5753
◯ 5754
◯ 5755
◯ 5756
◯ 5757
◯ 5758
◯ 5760
◯ 5762
◯ 5763
◯ 5764
◯ 5765
◯ 5766
◯ 5770
◯ 5771
◯ 5772
◯ 5773
◯ 5774
◯ 5775
◯ 5776
◯ 5777
◯ 5778
◯ 5779
◯ 5800
◯ 5801
◯ 5802
◯ 5803
◯ 5804
◯ 5805
◯ 5806
◯ 5807
◯ 5808
◯ 5810
◯ 5812
◯ 5820
◯ 5821
◯ 5822
◯ 5823
◯ 5824
◯ 5825
◯ 5826
◯ 5827
◯ 5828
◯ 5830
◯ 5831
◯ 5832
◯ 5833
◯ 5834
◯ 5835
◯ 5836
◯ 5837
◯ 5838
◯ 5840
◯ 5841
◯ 5842
◯ 5843
◯ 5844
◯ 5845
◯ 5846

◯ 5847
◯ 5848
◯ 5849
◯ 5850
◯ 5852
◯ 5853
◯ 5854
◯ 5855
◯ 5856
◯ 5857
◯ 5858
◯ 5860
◯ 5862
◯ 5863
◯ 5864
◯ 5865
◯ 5870
◯ 5871
◯ 5872
◯ 5873
◯ 5874
◯ 5875
◯ 5876
◯ 5877
◯ 5878
◯ 5879
◯ 5880
◯ 5883
◯ 5884
◯ 5885
◯ 5900
◯ 5901
◯ 5902
◯ 5903
◯ 5904
◯ 5905
◯ 5906
◯ 5907
◯ 5908
◯ 5910
◯ 5911
◯ 5920
◯ 5921
◯ 5922
◯ 5923
◯ 5924
◯ 5925
◯ 5926
◯ 5927
◯ 5930
◯ 5931
◯ 5932
◯ 5933
◯ 5934
◯ 5935
◯ 5940
◯ 5941
◯ 5942
◯ 5943
◯ 5944
◯ 5945
◯ 5946
◯ 5947
◯ 5948
◯ 5950
◯ 5952
◯ 5953
◯ 5954
◯ 5960
◯ 5962
◯ 5963

◯ 5964
◯ 5970
◯ 5971
◯ 5972
◯ 5973
◯ 5974
◯ 5975
◯ 5976
◯ 5977
◯ 5980
◯ 5981
◯ 5982
◯ 5983
◯ 5984
◯ 5985
◯ 5986
◯ 5987
◯ 5990
◯ 5991
◯ 5992
◯ 5993
◯ 6000
◯ 6001
◯ 6002
◯ 6003
◯ 6004
◯ 6005
◯ 6006
◯ 6007
◯ 6008
◯ 6009
◯ 6010
◯ 6011
◯ 6012
◯ 6013
◯ 6014
◯ 6015
◯ 6016
◯ 6017
◯ 6018
◯ 6019
◯ 6020
◯ 6030
◯ 6039
◯ 6040
◯ 6041
◯ 6042
◯ 6043
◯ 6044
◯ 6045
◯ 6046
◯ 6047
◯ 6048
◯ 6049
◯ 6050
◯ 6051
◯ 6052
◯ 6053
◯ 6054
◯ 6055
◯ 6056
◯ 6057
◯ 6058
◯ 6059
◯ 6060
◯ 6061
◯ 6062
◯ 6063
◯ 6064
◯ 6065
◯ 6066

◯ 6067
◯ 6068
◯ 6069
◯ 6070
◯ 6071
◯ 6080
◯ 6082
◯ 6090
◯ 6091
◯ 6092
◯ 6093
◯ 6094
◯ 6095
◯ 6096
◯ 6097
◯ 6100
◯ 6101
◯ 6102
◯ 6103
◯ 6104
◯ 6105
◯ 6106
◯ 6107
◯ 6108
◯ 6109
◯ 6110
◯ 6111
◯ 6112
◯ 6113
◯ 6120
◯ 6123
◯ 6124
◯ 6125
◯ 6126
◯ 6127
◯ 6128
◯ 6129
◯ 6130
◯ 6131
◯ 6132
◯ 6133
◯ 6134
◯ 6135
◯ 6136
◯ 6137
◯ 6140
◯ 6141
◯ 6142
◯ 6143
◯ 6144
◯ 6145
◯ 6146
◯ 6147
◯ 6148
◯ 6149
◯ 6150
◯ 6151
◯ 6152
◯ 6153
◯ 6154
◯ 6155
◯ 6156
◯ 6157
◯ 6160
◯ 6161
◯ 6162
◯ 6163
◯ 6164
◯ 6165
◯ 6170
◯ 6171

- ◯ 6172
- ◯ 6173
- ◯ 6174
- ◯ 6175
- ◯ 6176
- ◯ 6177
- ◯ 6179
- ◯ 6180
- ◯ 6181
- ◯ 6182
- ◯ 6183
- ◯ 6184
- ◯ 6185
- ◯ 6186
- ◯ 6187
- ◯ 6188
- ◯ 6191
- ◯ 6192
- ◯ 6193
- ◯ 6194
- ◯ 6195
- ◯ 6200
- ◯ 6201
- ◯ 6202
- ◯ 6203
- ◯ 6204
- ◯ 6205
- ◯ 6206
- ◯ 6207
- ◯ 6208
- ◯ 6209
- ◯ 6220
- ◯ 6221
- ◯ 6222
- ◯ 6223
- ◯ 6224
- ◯ 6225
- ◯ 6226
- ◯ 6227
- ◯ 6228
- ◯ 6229
- ◯ 6230
- ◯ 6231
- ◯ 6232
- ◯ 6233
- ◯ 6234
- ◯ 6235
- ◯ 6236
- ◯ 6237
- ◯ 6238
- ◯ 6240
- ◯ 6241
- ◯ 6242
- ◯ 6243
- ◯ 6244
- ◯ 6245
- ◯ 6246
- ◯ 6247
- ◯ 6248
- ◯ 6250
- ◯ 6251
- ◯ 6252
- ◯ 6253
- ◯ 6254
- ◯ 6255
- ◯ 6256
- ◯ 6257
- ◯ 6260
- ◯ 6261
- ◯ 6262
- ◯ 6263

○ 6264
○ 6265
○ 6266
○ 6267
○ 6268
○ 6269
○ 6270
○ 6271
○ 6272
○ 6273
○ 6275
○ 6276
○ 6277
○ 6278
○ 6279
○ 6280
○ 6281
○ 6282
○ 6283
○ 6284
○ 6285
○ 6286
○ 6287
○ 6288
○ 6289
○ 6290
○ 6291
○ 6292
○ 6293
○ 6294
○ 6295
○ 6296
○ 6297
○ 6298
○ 6299
○ 6300
○ 6301
○ 6302
○ 6303
○ 6304
○ 6305
○ 6306
○ 6307
○ 6308
○ 6309
○ 6310
○ 6311
○ 6312
○ 6313
○ 6314
○ 6315
○ 6316
○ 6317
○ 6318
○ 6319
○ 6330
○ 6333
○ 6334
○ 6335
○ 6336
○ 6337
○ 6338
○ 6339
○ 6340
○ 6343
○ 6344
○ 6345
○ 6346
○ 6347
○ 6350
○ 6353

○ 6354
○ 6355
○ 6356
○ 6360
○ 6362
○ 6363
○ 6364
○ 6365
○ 6366
○ 6367
○ 6370
○ 6373
○ 6374
○ 6375
○ 6376
○ 6377
○ 6378
○ 6380
○ 6382
○ 6383
○ 6384
○ 6385
○ 6386
○ 6387
○ 6388
○ 6390
○ 6393
○ 6394
○ 6395
○ 6396
○ 6400
○ 6401
○ 6402
○ 6403
○ 6404
○ 6405
○ 6406
○ 6407
○ 6408
○ 6409
○ 6410
○ 6411
○ 6412
○ 6413
○ 6414
○ 6415
○ 6416
○ 6417
○ 6418
○ 6419
○ 6440
○ 6442
○ 6443
○ 6444
○ 6445
○ 6446
○ 6447
○ 6448
○ 6449
○ 6450
○ 6460
○ 6462
○ 6463
○ 6464
○ 6465
○ 6466
○ 6467
○ 6468
○ 6470
○ 6472
○ 6473

- ◯ 6474
- ◯ 6475
- ◯ 6476
- ◯ 6477
- ◯ 6478
- ◯ 6479
- ◯ 6480
- ◯ 6483
- ◯ 6484
- ◯ 6485
- ◯ 6486
- ◯ 6487
- ◯ 6499
- ◯ 6500
- ◯ 6501
- ◯ 6502
- ◯ 6503
- ◯ 6504
- ◯ 6505
- ◯ 6506
- ◯ 6507
- ◯ 6508
- ◯ 6510
- ◯ 6511
- ◯ 6520
- ◯ 6521
- ◯ 6523
- ◯ 6524
- ◯ 6525
- ◯ 6526
- ◯ 6527
- ◯ 6528
- ◯ 6529
- ◯ 6530
- ◯ 6532
- ◯ 6533
- ◯ 6534
- ◯ 6535
- ◯ 6536
- ◯ 6540
- ◯ 6543
- ◯ 6544
- ◯ 6545
- ◯ 6546
- ◯ 6547
- ◯ 6548
- ◯ 6550
- ◯ 6552
- ◯ 6553
- ◯ 6554
- ◯ 6555
- ◯ 6556
- ◯ 6557
- ◯ 6558
- ◯ 6559
- ◯ 6560
- ◯ 6561
- ◯ 6562
- ◯ 6563
- ◯ 6564
- ◯ 6565
- ◯ 6566
- ◯ 6567
- ◯ 6568
- ◯ 6569
- ◯ 6570
- ◯ 6571
- ◯ 6572
- ◯ 6573
- ◯ 6574
- ◯ 6575

◯ 6576
◯ 6577
◯ 6578
◯ 6579
◯ 6580
◯ 6581
◯ 6589
◯ 6600
◯ 6601
◯ 6602
◯ 6603
◯ 6604
◯ 6605
◯ 6606
◯ 6607
◯ 6608
◯ 6609
◯ 6610
◯ 6611
◯ 6616
◯ 6620
◯ 6621
◯ 6622
◯ 6625
◯ 6627
◯ 6628
◯ 6640
◯ 6641
◯ 6642
◯ 6643
◯ 6644
◯ 6645
◯ 6650
◯ 6651
◯ 6652
◯ 6653
◯ 6654
◯ 6655
◯ 6660
◯ 6661
◯ 6662
◯ 6663
◯ 6664
◯ 6666
◯ 6667
◯ 6668
◯ 6669
◯ 6670
◯ 6671
◯ 6672
◯ 6673
◯ 6674
◯ 6675
◯ 6676
◯ 6677
◯ 6678
◯ 6680
◯ 6683
◯ 6684
◯ 6685
◯ 6686
◯ 6687
◯ 6690
◯ 6693
◯ 6694
◯ 6695
◯ 6696
◯ 6697
◯ 6700
◯ 6701
◯ 6702

- ◯ 6703
- ◯ 6704
- ◯ 6705
- ◯ 6706
- ◯ 6707
- ◯ 6710
- ◯ 6711
- ◯ 6712
- ◯ 6713
- ◯ 6714
- ◯ 6715
- ◯ 6720
- ◯ 6721
- ◯ 6722
- ◯ 6723
- ◯ 6726
- ◯ 6727
- ◯ 6730
- ◯ 6733
- ◯ 6734
- ◯ 6735
- ◯ 6736
- ◯ 6740
- ◯ 6741
- ◯ 6742
- ◯ 6743
- ◯ 6744
- ◯ 6745
- ◯ 6746
- ◯ 6747
- ◯ 6748
- ◯ 6749
- ◯ 6750
- ◯ 6751
- ◯ 6752
- ◯ 6753
- ◯ 6754
- ◯ 6755
- ◯ 6756
- ◯ 6757
- ◯ 6758
- ◯ 6760
- ◯ 6762
- ◯ 6763
- ◯ 6764
- ◯ 6765
- ◯ 6766
- ◯ 6767
- ◯ 6770
- ◯ 6773
- ◯ 6774
- ◯ 6775
- ◯ 6776
- ◯ 6780
- ◯ 6783
- ◯ 6784
- ◯ 6785
- ◯ 6786
- ◯ 6787
- ◯ 6788
- ◯ 6790
- ◯ 6795
- ◯ 6800
- ◯ 6801
- ◯ 6802
- ◯ 6803
- ◯ 6804
- ◯ 6805
- ◯ 6806
- ◯ 6807
- ◯ 6810

◯ 6811
◯ 6812
◯ 6813
◯ 6814
◯ 6815
◯ 6816
◯ 6817
◯ 6818
◯ 6819
◯ 6830
◯ 6831
◯ 6832
◯ 6833
◯ 6834
◯ 6835
◯ 6836
◯ 6837
◯ 6838
◯ 6840
◯ 6841
◯ 6842
◯ 6843
◯ 6844
◯ 6845
◯ 6846
◯ 6850
◯ 6851
◯ 6852
◯ 6853
◯ 6854
◯ 6858
◯ 6860
◯ 6862
◯ 6863
◯ 6864
◯ 6865
◯ 6866
◯ 6870
◯ 6871
◯ 6872
◯ 6873
◯ 6874
◯ 6875
◯ 6876
◯ 6877
◯ 6878
◯ 6879
◯ 6880
◯ 6881
◯ 6882
◯ 6883
◯ 6884
◯ 6885
◯ 6886
◯ 6887
◯ 6888
◯ 6890
◯ 6892
◯ 6893
◯ 6894
◯ 6895
◯ 6896
◯ 6897
◯ 6898
◯ 6900
◯ 6902
◯ 6903
◯ 6904
◯ 6910
◯ 6912
◯ 6913

- ◯ 6914
- ◯ 6915
- ◯ 6916
- ◯ 6917
- ◯ 6920
- ◯ 6921
- ◯ 6922
- ◯ 6930
- ◯ 6933
- ◯ 6934
- ◯ 6935
- ◯ 6936
- ◯ 7000
- ◯ 7001
- ◯ 7003
- ◯ 7004
- ◯ 7005
- ◯ 7006
- ◯ 7007
- ◯ 7008
- ◯ 7009
- ◯ 7011
- ◯ 7012
- ◯ 7013
- ◯ 7014
- ◯ 7015
- ◯ 7016
- ◯ 7017
- ◯ 7018
- ◯ 7019
- ◯ 7030
- ◯ 7031
- ◯ 7033
- ◯ 7034
- ◯ 7035
- ◯ 7036
- ◯ 7037
- ◯ 7038
- ◯ 7039
- ◯ 7040
- ◯ 7042
- ◯ 7043
- ◯ 7044
- ◯ 7045
- ◯ 7046
- ◯ 7047
- ◯ 7050
- ◯ 7051
- ◯ 7052
- ◯ 7053
- ◯ 7054
- ◯ 7055
- ◯ 7056
- ◯ 7057
- ◯ 7058
- ◯ 7059
- ◯ 7060
- ◯ 7061
- ◯ 7062
- ◯ 7063
- ◯ 7064
- ◯ 7065
- ◯ 7066
- ◯ 7070
- ◯ 7071
- ◯ 7072
- ◯ 7073
- ◯ 7074
- ◯ 7075
- ◯ 7076
- ◯ 7077

◯ 7078
◯ 7079
◯ 7080
◯ 7081
◯ 7082
◯ 7083
◯ 7087
◯ 7088
◯ 7089
◯ 7100
◯ 7101
◯ 7102
◯ 7103
◯ 7104
◯ 7105
◯ 7106
◯ 7107
◯ 7108
◯ 7110
◯ 7111
◯ 7112
◯ 7113
◯ 7114
◯ 7115
◯ 7116
◯ 7117
◯ 7120
◯ 7121
◯ 7122
◯ 7123
◯ 7124
◯ 7125
◯ 7126
◯ 7127
◯ 7128
◯ 7129
◯ 7130
◯ 7131
◯ 7132
◯ 7133
◯ 7134
◯ 7135
◯ 7136
◯ 7137
◯ 7140
◯ 7141
◯ 7142
◯ 7143
◯ 7144
◯ 7145
◯ 7146
◯ 7147
◯ 7148
◯ 7149
◯ 7160
◯ 7161
◯ 7163
◯ 7164
◯ 7165
◯ 7166
◯ 7167
◯ 7170
◯ 7171
◯ 7172
◯ 7173
◯ 7174
◯ 7175
◯ 7176
◯ 7177
◯ 7180
◯ 7182

◯ 7183
◯ 7184
◯ 7185
◯ 7186
◯ 7190
◯ 7191
◯ 7192
◯ 7193
◯ 7194
◯ 7195
◯ 7196
◯ 7197
◯ 7199
◯ 7200
◯ 7201
◯ 7202
◯ 7203
◯ 7204
◯ 7205
◯ 7206
◯ 7207
◯ 7208
◯ 7209
◯ 7210
◯ 7211
◯ 7212
◯ 7213
◯ 7214
◯ 7215
◯ 7216
◯ 7217
◯ 7218
◯ 7219
◯ 7220
◯ 7221
◯ 7222
◯ 7223
◯ 7225
◯ 7226
◯ 7229
◯ 7230
◯ 7231
◯ 7232
◯ 7233
◯ 7234
◯ 7235
◯ 7236
◯ 7237
◯ 7238
◯ 7239
◯ 7240
◯ 7241
◯ 7242
◯ 7243
◯ 7244
◯ 7245
◯ 7246
◯ 7247
◯ 7248
◯ 7250
◯ 7251
◯ 7252
◯ 7253
◯ 7254
◯ 7255
◯ 7256
◯ 7257
◯ 7258
◯ 7260
◯ 7261
◯ 7262

- ◯ 7263
- ◯ 7264
- ◯ 7265
- ◯ 7266
- ◯ 7267
- ◯ 7268
- ◯ 7270
- ◯ 7271
- ◯ 7272
- ◯ 7273
- ◯ 7274
- ◯ 7275
- ◯ 7276
- ◯ 7277
- ◯ 7280
- ◯ 7281
- ◯ 7282
- ◯ 7283
- ◯ 7284
- ◯ 7285
- ◯ 7286
- ◯ 7290
- ◯ 7291
- ◯ 7292
- ◯ 7293
- ◯ 7294
- ◯ 7295
- ◯ 7300
- ◯ 7301
- ◯ 7302
- ◯ 7303
- ◯ 7304
- ◯ 7305
- ◯ 7306
- ◯ 7307
- ◯ 7308
- ◯ 7309
- ◯ 7310
- ◯ 7311
- ◯ 7312
- ◯ 7313
- ◯ 7314
- ◯ 7315
- ◯ 7316
- ◯ 7317
- ◯ 7318
- ◯ 7319
- ◯ 7330
- ◯ 7334
- ◯ 7340
- ◯ 7342
- ◯ 7343
- ◯ 7344
- ◯ 7345
- ◯ 7346
- ◯ 7348
- ◯ 7349
- ◯ 7350
- ◯ 7352
- ◯ 7353
- ◯ 7354
- ◯ 7355
- ◯ 7356
- ◯ 7357
- ◯ 7360
- ◯ 7362
- ◯ 7363
- ◯ 7364
- ◯ 7365
- ◯ 7366
- ◯ 7367

◯ 7370
◯ 7371
◯ 7372
◯ 7373
◯ 7374
◯ 7375
◯ 7376
◯ 7377
◯ 7380
◯ 7383
◯ 7384
◯ 7385
◯ 7386
◯ 7390
◯ 7393
◯ 7394
◯ 7395
◯ 7396
◯ 7400
◯ 7401
◯ 7402
◯ 7403
◯ 7404
◯ 7405
◯ 7406
◯ 7407
◯ 7408
◯ 7410
◯ 7411
◯ 7412
◯ 7413
◯ 7414
◯ 7415
◯ 7416
◯ 7417
◯ 7418
◯ 7419
◯ 7430
◯ 7433
◯ 7434
◯ 7435
◯ 7436
◯ 7437
◯ 7440
◯ 7442
◯ 7443
◯ 7444
◯ 7445
◯ 7446
◯ 7447
◯ 7450
◯ 7452
◯ 7453
◯ 7454
◯ 7455
◯ 7456
◯ 7457
◯ 7460
◯ 7463
◯ 7464
◯ 7465
◯ 7470
◯ 7472
◯ 7473
◯ 7474
◯ 7475
◯ 7476
◯ 7480
◯ 7481
◯ 7482
◯ 7483

◯ 7484
◯ 7485
◯ 7486
◯ 7487
◯ 7488
◯ 7490
◯ 7493
◯ 7494
◯ 7495
◯ 7496
◯ 7500
◯ 7501
◯ 7502
◯ 7503
◯ 7504
◯ 7505
◯ 7506
◯ 7507
◯ 7508
◯ 7509
◯ 7510
◯ 7511
◯ 7512
◯ 7513
◯ 7514
◯ 7515
◯ 7516
◯ 7517
◯ 7518
◯ 7520
◯ 7521
◯ 7522
◯ 7523
◯ 7524
◯ 7525
◯ 7526
◯ 7527
◯ 7528
◯ 7530
◯ 7531
◯ 7532
◯ 7533
◯ 7534
◯ 7535
◯ 7536
◯ 7537
◯ 7538
◯ 7539
◯ 7540
◯ 7541
◯ 7542
◯ 7543
◯ 7544
◯ 7545
◯ 7546
◯ 7547
◯ 7548
◯ 7549
◯ 7550
◯ 7555
◯ 7556
◯ 7557
◯ 7559
◯ 7560
◯ 7561
◯ 7563
◯ 7564
◯ 7565
◯ 7566
◯ 7567
◯ 7568

- ◯ 7569
- ◯ 7570
- ◯ 7571
- ◯ 7575
- ◯ 7576
- ◯ 7577
- ◯ 7578
- ◯ 7579
- ◯ 7580
- ◯ 7583
- ◯ 7584
- ◯ 7585
- ◯ 7586
- ◯ 7588
- ◯ 7590
- ◯ 7591
- ◯ 7592
- ◯ 7593
- ◯ 7594
- ◯ 7595
- ◯ 7596
- ◯ 7597
- ◯ 7598
- ◯ 7599
- ◯ 7600
- ◯ 7601
- ◯ 7602
- ◯ 7603
- ◯ 7604
- ◯ 7605
- ◯ 7606
- ◯ 7607
- ◯ 7608
- ◯ 7609
- ◯ 7610
- ◯ 7611
- ◯ 7612
- ◯ 7613
- ◯ 7614
- ◯ 7615
- ◯ 7616
- ◯ 7617
- ◯ 7618
- ◯ 7619
- ◯ 7620
- ◯ 7621
- ◯ 7622
- ◯ 7623
- ◯ 7624
- ◯ 7625
- ◯ 7626
- ◯ 7627
- ◯ 7630
- ◯ 7631
- ◯ 7635
- ◯ 7636
- ◯ 7637
- ◯ 7638
- ◯ 7640
- ◯ 7642
- ◯ 7643
- ◯ 7644
- ◯ 7645
- ◯ 7646
- ◯ 7647
- ◯ 7648
- ◯ 7649
- ◯ 7650
- ◯ 7651
- ◯ 7652
- ◯ 7653

◯ 7654
◯ 7655
◯ 7656
◯ 7657
◯ 7659
◯ 7662
◯ 7663
◯ 7664
◯ 7665
◯ 7666
◯ 7667
◯ 7668
◯ 7669
◯ 7670
◯ 7671
◯ 7679
◯ 7680
◯ 7682
◯ 7683
◯ 7684
◯ 7685
◯ 7686
◯ 7687
◯ 7688
◯ 7689
◯ 7690
◯ 7693
◯ 7694
◯ 7695
◯ 7700
◯ 7701
◯ 7702
◯ 7703
◯ 7704
◯ 7705
◯ 7706
◯ 7707
◯ 7708
◯ 7709
◯ 7720
◯ 7721
◯ 7722
◯ 7723
◯ 7724
◯ 7725
◯ 7726
◯ 7727
◯ 7728
◯ 7729
◯ 7730
◯ 7731
◯ 7732
◯ 7733
◯ 7734
◯ 7735
◯ 7736
◯ 7737
◯ 7738
◯ 7739
◯ 7740
◯ 7741
◯ 7742
◯ 7743
◯ 7744
◯ 7745
◯ 7746
◯ 7747
◯ 7748
◯ 7749
◯ 7750
◯ 7751

◯ 7752
◯ 7753
◯ 7754
◯ 7755
◯ 7756
◯ 7757
◯ 7758
◯ 7759
◯ 7761
◯ 7762
◯ 7763
◯ 7764
◯ 7765
◯ 7766
◯ 7767
◯ 7770
◯ 7771
◯ 7772
◯ 7780
◯ 7783
◯ 7784
◯ 7785
◯ 7786
◯ 7787
◯ 7788
◯ 7790
◯ 7795
◯ 7796
◯ 7797
◯ 7798
◯ 7799
◯ 7800
◯ 7801
◯ 7802
◯ 7803
◯ 7804
◯ 7805
◯ 7806
◯ 7807
◯ 7810
◯ 7811
◯ 7812
◯ 7813
◯ 7814
◯ 7815
◯ 7816
◯ 7820
◯ 7821
◯ 7822
◯ 7823
◯ 7824
◯ 7825
◯ 7826
◯ 7827
◯ 7828
◯ 7829
◯ 7833
◯ 7834
◯ 7835
◯ 7836
◯ 7837
◯ 7838
◯ 7839
◯ 7840
◯ 7841
◯ 7842
◯ 7846
◯ 7847
◯ 7848
◯ 7850
◯ 7851

◯ 7852
◯ 7853
◯ 7854
◯ 7855
◯ 7856
◯ 7857
◯ 7858
◯ 7859
◯ 7860
◯ 7861
◯ 7862
◯ 7863
◯ 7864
◯ 7865
◯ 7866
◯ 7867
◯ 7868
◯ 7869
◯ 7870
◯ 7871
◯ 7872
◯ 7873
◯ 7874
◯ 7875
◯ 7876
◯ 7877
◯ 7878
◯ 7879
◯ 7880
◯ 7882
◯ 7883
◯ 7884
◯ 7885
◯ 7886
◯ 7887
◯ 7888
◯ 7893
◯ 7894
◯ 7895
◯ 7896
◯ 7900
◯ 7901
◯ 7902
◯ 7903
◯ 7904
◯ 7905
◯ 7906
◯ 7907
◯ 7908
◯ 7909
◯ 7910
◯ 7911
◯ 7912
◯ 7915
◯ 7916
◯ 7917
◯ 7918
◯ 7920
◯ 7922
◯ 7923
◯ 7924
◯ 7925
◯ 7926
◯ 7931
◯ 7932
◯ 7933
◯ 7934
◯ 7935
◯ 7936
◯ 7937
◯ 7938

- ◯ 7940
- ◯ 7941
- ◯ 7942
- ◯ 7943
- ◯ 7945
- ◯ 7946
- ◯ 7949
- ◯ 7950
- ◯ 7951
- ◯ 7952
- ◯ 7953
- ◯ 7954
- ◯ 7955
- ◯ 7956
- ◯ 7960
- ◯ 7969
- ◯ 7970
- ◯ 7971
- ◯ 7972
- ◯ 7973
- ◯ 7974
- ◯ 7975
- ◯ 7976
- ◯ 7977
- ◯ 7978
- ◯ 7982
- ◯ 7983
- ◯ 7984
- ◯ 7985
- ◯ 7990
- ◯ 7991
- ◯ 7992
- ◯ 7993
- ◯ 7994
- ◯ 7995
- ◯ 7996
- ◯ 7997
- ◯ 7998
- ◯ 7999
- ◯ 8000
- ◯ 8001
- ◯ 8002
- ◯ 8003
- ◯ 8004
- ◯ 8010
- ◯ 8011
- ◯ 8012
- ◯ 8013
- ◯ 8015
- ◯ 8016
- ◯ 8020
- ◯ 8021
- ◯ 8022
- ◯ 8023
- ◯ 8024
- ◯ 8025
- ◯ 8026
- ◯ 8027
- ◯ 8028
- ◯ 8029
- ◯ 8030
- ◯ 8031
- ◯ 8040
- ◯ 8041
- ◯ 8042
- ◯ 8043
- ◯ 8044
- ◯ 8045
- ◯ 8046
- ◯ 8047
- ◯ 8048

◯ 8049
◯ 8050
◯ 8051
◯ 8052
◯ 8053
◯ 8054
◯ 8055
◯ 8060
◯ 8061
◯ 8062
◯ 8063
◯ 8064
◯ 8065
◯ 8070
◯ 8072
◯ 8073
◯ 8074
◯ 8075
◯ 8080
◯ 8081
◯ 8082
◯ 8083
◯ 8084
◯ 8086
◯ 8090
◯ 8091
◯ 8092
◯ 8093
◯ 8094
◯ 8095
◯ 8096
◯ 8097
◯ 8099
◯ 8100
◯ 8101
◯ 8102
◯ 8103
◯ 8104
◯ 8105
◯ 8106
◯ 8107
◯ 8108
◯ 8109
◯ 8110
◯ 8112
◯ 8113
◯ 8114
◯ 8115
◯ 8120
◯ 8121
◯ 8122
◯ 8123
◯ 8124
◯ 8125
◯ 8129
◯ 8130
◯ 8132
◯ 8133
◯ 8140
◯ 8141
◯ 8142
◯ 8143
◯ 8150
◯ 8152
◯ 8160
◯ 8161
◯ 8162
◯ 8163
◯ 8164
◯ 8165
◯ 8200

◯ 8201
◯ 8205
◯ 8206
◯ 8207
◯ 8208
◯ 8219
◯ 8220
◯ 8221
◯ 8222
◯ 8224
◯ 8230
◯ 8231
◯ 8232
◯ 8233
◯ 8240
◯ 8241
◯ 8242
◯ 8243
◯ 8244
◯ 8245
◯ 8250
◯ 8251
◯ 8252
◯ 8260
◯ 8261
◯ 8262
◯ 8263
◯ 8264
◯ 8270
◯ 8271
◯ 8272
◯ 8273
◯ 8280
◯ 8283
◯ 8284
◯ 8290
◯ 8292
◯ 8293
◯ 8294
◯ 8300
◯ 8301
◯ 8302
◯ 8310
◯ 8311
◯ 8312
◯ 8320
◯ 8321
◯ 8322
◯ 8323
◯ 8324
◯ 8325
◯ 8326
◯ 8327
◯ 8328
◯ 8330
◯ 8331
◯ 8332
◯ 8333
◯ 8334
◯ 8335
◯ 8340
◯ 8341
◯ 8342
◯ 8343
◯ 8344
◯ 8345
◯ 8346
◯ 8350
◯ 8352
◯ 8353
◯ 8354

◯ 8355
◯ 8360
◯ 8361
◯ 8362
◯ 8363
◯ 8364
◯ 8365
◯ 8366
◯ 8367
◯ 8368
◯ 8370
◯ 8371
◯ 8372
◯ 8373
◯ 8375
◯ 8379
◯ 8380
◯ 8381
◯ 8382
◯ 8383
◯ 8384
◯ 8385
◯ 8386
◯ 8387
◯ 8400
◯ 8401
◯ 8402
◯ 8403
◯ 8404
◯ 8405
◯ 8406
◯ 8407
◯ 8408
◯ 8409
◯ 8410
◯ 8411
◯ 8412
◯ 8413
◯ 8414
◯ 8415
◯ 8416
◯ 8417
◯ 8418
◯ 8419
◯ 8420
◯ 8424
◯ 8430
◯ 8431
◯ 8432
◯ 8433
◯ 8434
◯ 8440
◯ 8441
◯ 8450
◯ 8451
◯ 8452
◯ 8453
◯ 8454
◯ 8460
◯ 8462
◯ 8463
◯ 8464
◯ 8465
◯ 8466
◯ 8470
◯ 8471
◯ 8472
◯ 8473
◯ 8474
◯ 8475
◯ 8476

- ◯ 8477
- ◯ 8478
- ◯ 8479
- ◯ 8500
- ◯ 8501
- ◯ 8502
- ◯ 8503
- ◯ 8504
- ◯ 8505
- ◯ 8506
- ◯ 8507
- ◯ 8508
- ◯ 8511
- ◯ 8512
- ◯ 8513
- ◯ 8514
- ◯ 8517
- ◯ 8519
- ◯ 8520
- ◯ 8521
- ◯ 8522
- ◯ 8523
- ◯ 8524
- ◯ 8525
- ◯ 8526
- ◯ 8527
- ◯ 8528
- ◯ 8529
- ◯ 8530
- ◯ 8531
- ◯ 8532
- ◯ 8533
- ◯ 8534
- ◯ 8535
- ◯ 8536
- ◯ 8537
- ◯ 8538
- ◯ 8539
- ◯ 8550
- ◯ 8553
- ◯ 8554
- ◯ 8555
- ◯ 8560
- ◯ 8561
- ◯ 8562
- ◯ 8563
- ◯ 8564
- ◯ 8565
- ◯ 8567
- ◯ 8570
- ◯ 8571
- ◯ 8572
- ◯ 8573
- ◯ 8574
- ◯ 8575
- ◯ 8577
- ◯ 8590
- ◯ 8591
- ◯ 8592
- ◯ 8593
- ◯ 8594
- ◯ 8600
- ◯ 8601
- ◯ 8602
- ◯ 8603
- ◯ 8604
- ◯ 8605
- ◯ 8630
- ◯ 8631
- ◯ 8632
- ◯ 8633

◯ 8634
◯ 8635
◯ 8640
◯ 8641
◯ 8642
◯ 8643
◯ 8644
◯ 8650
◯ 8651
◯ 8652
◯ 8653
◯ 8654
◯ 8655
◯ 8700
◯ 8701
◯ 8702
◯ 8703
◯ 8704
◯ 8705
◯ 8706
◯ 8707
◯ 8708
◯ 8710
◯ 8711
◯ 8712
◯ 8713
◯ 8714
◯ 8715
◯ 8717
◯ 8718
◯ 8719
◯ 8730
◯ 8731
◯ 8732
◯ 8734
◯ 8735
◯ 8736
◯ 8737
◯ 8740
◯ 8741
◯ 8742
◯ 8745
◯ 8746
◯ 8749
◯ 8750
◯ 8751
◯ 8752
◯ 8753
◯ 8754
◯ 8755
◯ 8756
◯ 8757
◯ 8758
◯ 8759
◯ 8765
◯ 8770
◯ 8771
◯ 8772
◯ 8773
◯ 8774
◯ 8775
◯ 8780
◯ 8782
◯ 8783
◯ 8790
◯ 8793
◯ 8794
◯ 8800
◯ 8801
◯ 8802
◯ 8803

- ◯ 8804
- ◯ 8805
- ◯ 8806
- ◯ 8807
- ◯ 8808
- ◯ 8810
- ◯ 8811
- ◯ 8812
- ◯ 8813
- ◯ 8820
- ◯ 8821
- ◯ 8822
- ◯ 8823
- ◯ 8824
- ◯ 8825
- ◯ 8826
- ◯ 8830
- ◯ 8831
- ◯ 8832
- ◯ 8833
- ◯ 8834
- ◯ 8835
- ◯ 8840
- ◯ 8841
- ◯ 8842
- ◯ 8843
- ◯ 8851
- ◯ 8852
- ◯ 8853
- ◯ 8854
- ◯ 8855
- ◯ 8856
- ◯ 8857
- ◯ 8858
- ◯ 8859
- ◯ 8888
- ◯ 8890
- ◯ 8900
- ◯ 8901
- ◯ 8902
- ◯ 8903
- ◯ 8904
- ◯ 8905
- ◯ 8906
- ◯ 8907
- ◯ 8908
- ◯ 8910
- ◯ 8911
- ◯ 8912
- ◯ 8913
- ◯ 8914
- ◯ 8915
- ◯ 8916
- ◯ 8917
- ◯ 8918
- ◯ 8919
- ◯ 8930
- ◯ 8931
- ◯ 8940
- ◯ 8941
- ◯ 8942
- ◯ 8943
- ◯ 8944
- ◯ 8945
- ◯ 8946
- ◯ 8949
- ◯ 8950
- ◯ 8951
- ◯ 8952
- ◯ 8953
- ◯ 8955

◯ 8957
◯ 8959
◯ 8970
◯ 8971
◯ 8972
◯ 8980
◯ 8981
◯ 8982
◯ 8983
◯ 8988
◯ 9000
◯ 9001
◯ 9002
◯ 9003
◯ 9004
◯ 9005
◯ 9006
◯ 9007
◯ 9008
◯ 9009
◯ 9013
◯ 9018
◯ 9020
◯ 9021
◯ 9022
◯ 9023
◯ 9024
◯ 9025
◯ 9026
◯ 9027
◯ 9028
◯ 9029
◯ 9030
◯ 9031
◯ 9040
◯ 9041
◯ 9050
◯ 9051
◯ 9060
◯ 9061
◯ 9062
◯ 9063
◯ 9064
◯ 9065
◯ 9066
◯ 9067
◯ 9068
◯ 9070
◯ 9071
◯ 9072
◯ 9073
◯ 9074
◯ 9075
◯ 9080
◯ 9081
◯ 9082
◯ 9083
◯ 9084
◯ 9085
◯ 9089
◯ 9100
◯ 9101
◯ 9102
◯ 9103
◯ 9104
◯ 9106
◯ 9107
◯ 9110
◯ 9111
◯ 9112
◯ 9118

◯ 9119
◯ 9120
◯ 9121
◯ 9122
◯ 9130
◯ 9131
◯ 9132
◯ 9133
◯ 9134
◯ 9135
◯ 9136
◯ 9137
◯ 9138
◯ 9139
◯ 9140
◯ 9141
◯ 9142
◯ 9143
◯ 9147
◯ 9148
◯ 9149
◯ 9150
◯ 9151
◯ 9152
◯ 9160
◯ 9161
◯ 9170
◯ 9171
◯ 9172
◯ 9173
◯ 9174
◯ 9175
◯ 9176
◯ 9177
◯ 9178
◯ 9179
◯ 9180
◯ 9189
◯ 9190
◯ 9191
◯ 9192
◯ 9193
◯ 9194
◯ 9195
◯ 9196
◯ 9197
◯ 9198
◯ 9200
◯ 9201
◯ 9202
◯ 9203
◯ 9204
◯ 9205
◯ 9206
◯ 9207
◯ 9208
◯ 9209
◯ 9210
◯ 9211
◯ 9212
◯ 9213
◯ 9214
◯ 9215
◯ 9216
◯ 9217
◯ 9218
◯ 9219
◯ 9220
◯ 9221
◯ 9222
◯ 9223

○ 9224
○ 9225
○ 9226
○ 9227
○ 9228
○ 9230
○ 9231
○ 9232
○ 9233
○ 9234
○ 9235
○ 9236
○ 9237
○ 9238
○ 9239
○ 9240
○ 9241
○ 9242
○ 9250
○ 9251
○ 9252
○ 9253
○ 9254
○ 9255
○ 9256
○ 9257
○ 9258
○ 9259
○ 9260
○ 9261
○ 9262
○ 9263
○ 9264
○ 9265
○ 9266
○ 9267
○ 9268
○ 9269
○ 9270
○ 9271
○ 9272
○ 9273
○ 9278
○ 9279
○ 9280
○ 9281
○ 9282
○ 9283
○ 9284
○ 9285
○ 9286
○ 9287
○ 9288
○ 9289
○ 9300
○ 9301
○ 9302
○ 9303
○ 9304
○ 9306
○ 9309
○ 9310
○ 9311
○ 9312
○ 9313
○ 9314
○ 9315
○ 9316
○ 9319
○ 9320
○ 9321

◯ 9322
◯ 9323
◯ 9324
◯ 9325
◯ 9326
◯ 9327
◯ 9328
◯ 9329
◯ 9330
◯ 9331
◯ 9338
◯ 9339
◯ 9340
◯ 9341
◯ 9342
◯ 9343
◯ 9344
◯ 9345
◯ 9346
◯ 9347
◯ 9348
◯ 9350
◯ 9351
◯ 9352
◯ 9353
◯ 9354
◯ 9355
◯ 9356
◯ 9358
◯ 9359
◯ 9360
◯ 9361
◯ 9362
◯ 9363
◯ 9364
◯ 9365
◯ 9366
◯ 9367
◯ 9370
◯ 9371
◯ 9372
◯ 9373
◯ 9374
◯ 9375
◯ 9376
◯ 9377
◯ 9378
◯ 9379
◯ 9384
◯ 9388
◯ 9390
◯ 9391
◯ 9392
◯ 9393
◯ 9394
◯ 9395
◯ 9396
◯ 9400
◯ 9401
◯ 9402
◯ 9403
◯ 9404
◯ 9406
◯ 9407
◯ 9408
◯ 9410
◯ 9411
◯ 9412
◯ 9413
◯ 9414
◯ 9415

◯ 9416
◯ 9417
◯ 9418
◯ 9420
◯ 9421
◯ 9422
◯ 9423
◯ 9424
◯ 9425
◯ 9426
◯ 9427
◯ 9428
◯ 9429
◯ 9430
◯ 9440
◯ 9449
◯ 9450
◯ 9451
◯ 9452
◯ 9453
◯ 9454
◯ 9455
◯ 9456
◯ 9457
◯ 9458
◯ 9459
◯ 9460
◯ 9461
◯ 9462
◯ 9464
◯ 9465
◯ 9466
◯ 9470
◯ 9471
◯ 9472
◯ 9480
◯ 9482
◯ 9485
◯ 9490
◯ 9491
◯ 9492
◯ 9493
◯ 9494
◯ 9495
◯ 9496
◯ 9497
◯ 9499
◯ 9500
◯ 9501
◯ 9502
◯ 9503
◯ 9504
◯ 9505
◯ 9506
◯ 9507
◯ 9510
◯ 9511
◯ 9512
◯ 9513
◯ 9514
◯ 9515
◯ 9516
◯ 9517
◯ 9519
◯ 9520
◯ 9521
◯ 9522
◯ 9523
◯ 9524
◯ 9525
◯ 9526

○ 9529
○ 9530
○ 9531
○ 9532
○ 9533
○ 9534
○ 9535
○ 9536
○ 9537
○ 9538
○ 9539
○ 9540
○ 9541
○ 9542
○ 9543
○ 9544
○ 9545
○ 9546
○ 9547
○ 9548
○ 9549
○ 9550
○ 9551
○ 9552
○ 9553
○ 9554
○ 9555
○ 9556
○ 9557
○ 9558
○ 9559
○ 9560
○ 9561
○ 9562
○ 9563
○ 9564
○ 9565
○ 9566
○ 9567
○ 9568
○ 9569
○ 9570
○ 9571
○ 9572
○ 9573
○ 9574
○ 9575
○ 9576
○ 9577
○ 9579
○ 9581
○ 9582
○ 9583
○ 9584
○ 9585
○ 9586
○ 9589
○ 9590
○ 9591
○ 9592
○ 9593
○ 9594
○ 9595
○ 9596
○ 9597
○ 9598
○ 9599
○ 9600
○ 9601
○ 9602
○ 9603

◯ 9604
◯ 9605
◯ 9606
◯ 9607
◯ 9608
◯ 9609
◯ 9610
◯ 9611
◯ 9612
◯ 9613
◯ 9614
◯ 9615
◯ 9616
◯ 9620
◯ 9621
◯ 9622
◯ 9625
◯ 9626
◯ 9627
◯ 9628
◯ 9629
◯ 9630
◯ 9631
◯ 9632
◯ 9633
◯ 9634
◯ 9635
◯ 9636
◯ 9637
◯ 9638
◯ 9640
◯ 9650
◯ 9651
◯ 9652
◯ 9653
◯ 9654
◯ 9655
◯ 9656
◯ 9657
◯ 9659
◯ 9660
◯ 9661
◯ 9662
◯ 9663
◯ 9664
◯ 9665
◯ 9666
◯ 9667
◯ 9668
◯ 9669
◯ 9670
◯ 9671
◯ 9672
◯ 9673
◯ 9674
◯ 9675
◯ 9676
◯ 9677
◯ 9678
◯ 9679
◯ 9680
◯ 9681
◯ 9682
◯ 9683
◯ 9684
◯ 9685
◯ 9686
◯ 9689
◯ 9691
◯ 9692
◯ 9693

◯ 9694
◯ 9695
◯ 9696
◯ 9697
◯ 9700
◯ 9701
◯ 9702
◯ 9703
◯ 9704
◯ 9705
◯ 9706
◯ 9707
◯ 9708
◯ 9710
◯ 9711
◯ 9712
◯ 9713
◯ 9714
◯ 9720
◯ 9721
◯ 9722
◯ 9723
◯ 9724
◯ 9725
◯ 9726
◯ 9728
◯ 9729
◯ 9730
◯ 9731
◯ 9732
◯ 9733
◯ 9734
◯ 9735
◯ 9736
◯ 9737
◯ 9738
◯ 9739
◯ 9740
◯ 9741
◯ 9742
◯ 9743
◯ 9744
◯ 9745
◯ 9746
◯ 9747
◯ 9748
◯ 9749
◯ 9750
◯ 9752
◯ 9753
◯ 9754
◯ 9760
◯ 9762
◯ 9763
◯ 9764
◯ 9770
◯ 9771
◯ 9772
◯ 9773
◯ 9774
◯ 9775
◯ 9776
◯ 9780
◯ 9781
◯ 9782
◯ 9783
◯ 9784
◯ 9785
◯ 9786
◯ 9787
◯ 9788

○ 9790
○ 9791
○ 9792
○ 9800
○ 9801
○ 9802
○ 9803
○ 9804
○ 9805
○ 9806
○ 9807
○ 9808
○ 9809
○ 9810
○ 9811
○ 9812
○ 9813
○ 9814
○ 9815
○ 9816
○ 9817
○ 9818
○ 9819
○ 9820
○ 9821
○ 9822
○ 9823
○ 9824
○ 9825
○ 9826
○ 9827
○ 9828
○ 9829
○ 9830
○ 9831
○ 9832
○ 9833
○ 9834
○ 9835
○ 9836
○ 9837
○ 9838
○ 9839
○ 9840
○ 9841
○ 9842
○ 9843
○ 9844
○ 9846
○ 9847
○ 9848
○ 9849
○ 9850
○ 9851
○ 9852
○ 9853
○ 9854
○ 9855
○ 9856
○ 9857
○ 9858
○ 9859
○ 9860
○ 9861
○ 9862
○ 9863
○ 9864
○ 9865
○ 9866
○ 9867
○ 9868

◯ 9880
◯ 9881
◯ 9882
◯ 9883
◯ 9884
◯ 9885
◯ 9886
◯ 9890
◯ 9892
◯ 9893
◯ 9894
◯ 9895
◯ 9900
◯ 9901
◯ 9902
◯ 9903
◯ 9904
◯ 9910
◯ 9911
◯ 9912
◯ 9913
◯ 9914
◯ 9915
◯ 9916
◯ 9917
◯ 9918
◯ 9920
◯ 9921
◯ 9922
◯ 9925
◯ 9926
◯ 9930
◯ 9932
◯ 9933
◯ 9934
◯ 9935
◯ 9936
◯ 9937
◯ 9940
◯ 9950
◯ 9951
◯ 9952
◯ 9953
◯ 9954
◯ 9955
◯ 9956
◯ 9957
◯ 9958
◯ 9959
◯ 9960
◯ 9961
◯ 9962
◯ 9963
◯ 9964
◯ 9965
◯ 9966
◯ 9967
◯ 9968
◯ 9969
◯ 9970
◯ 9971
◯ 9972
◯ 9973
◯ 9974
◯ 9975
◯ 9976
◯ 9977
◯ 9978
◯ 9979
◯ 9980
◯ 9981

◯ 9982
◯ 9983
◯ 9984
◯ 9985
◯ 9990
◯ 9991
◯ 9992
◯ 9995
◯ Homeless
◯ Missing

If your institution will not allow a 4-digit zip code please enter the first 3 digits here (leave the field above this for 4-digits blank)

◯ 005
◯ 006
◯ 007
◯ 008
◯ 009
◯ 010
◯ 011
◯ 012
◯ 013
◯ 014
◯ 015
◯ 016
◯ 017
◯ 018
◯ 019
◯ 020
◯ 021
◯ 022
◯ 023
◯ 024
◯ 025
◯ 026
◯ 027
◯ 028
◯ 029
◯ 030
◯ 031
◯ 032
◯ 033
◯ 034
◯ 035
◯ 036
◯ 037
◯ 038
◯ 039
◯ 040
◯ 041
◯ 042
◯ 043
◯ 044
◯ 045
◯ 046
◯ 047
◯ 048
◯ 049
◯ 050
◯ 051
◯ 052
◯ 053
◯ 054
◯ 055
◯ 056
◯ 057
◯ 058
◯ 059
◯ 060
◯ 061
◯ 062
◯ 063
◯ 064
◯ 065
◯ 066
◯ 067
◯ 068
◯ 069
◯ 070
◯ 071
◯ 072
◯ 073

○ 074
○ 075
○ 076
○ 077
○ 078
○ 079
○ 080
○ 081
○ 082
○ 083
○ 084
○ 085
○ 086
○ 087
○ 088
○ 089
○ 090
○ 091
○ 092
○ 093
○ 094
○ 095
○ 096
○ 097
○ 098
○ 099
○ 100
○ 101
○ 102
○ 103
○ 104
○ 105
○ 106
○ 107
○ 108
○ 109
○ 110
○ 111
○ 112
○ 113
○ 114
○ 115
○ 116
○ 117
○ 118
○ 119
○ 120
○ 121
○ 122
○ 123
○ 124
○ 125
○ 126
○ 127
○ 128
○ 129
○ 130
○ 131
○ 132
○ 133
○ 134
○ 135
○ 136
○ 137
○ 138
○ 139
○ 140
○ 141
○ 142
○ 143
○ 144

◯ 145
◯ 146
◯ 147
◯ 148
◯ 149
◯ 150
◯ 151
◯ 152
◯ 153
◯ 154
◯ 155
◯ 156
◯ 157
◯ 158
◯ 159
◯ 160
◯ 161
◯ 162
◯ 163
◯ 164
◯ 165
◯ 166
◯ 167
◯ 168
◯ 169
◯ 170
◯ 171
◯ 172
◯ 173
◯ 174
◯ 175
◯ 176
◯ 177
◯ 178
◯ 179
◯ 180
◯ 181
◯ 182
◯ 183
◯ 184
◯ 185
◯ 186
◯ 187
◯ 188
◯ 189
◯ 190
◯ 191
◯ 192
◯ 193
◯ 194
◯ 195
◯ 196
◯ 197
◯ 198
◯ 199
◯ 200
◯ 201
◯ 202
◯ 203
◯ 204
◯ 205
◯ 206
◯ 207
◯ 208
◯ 209
◯ 210
◯ 211
◯ 212
◯ 214
◯ 215
◯ 216

◯ 217
◯ 218
◯ 219
◯ 220
◯ 221
◯ 222
◯ 223
◯ 224
◯ 225
◯ 226
◯ 227
◯ 228
◯ 229
◯ 230
◯ 231
◯ 232
◯ 233
◯ 234
◯ 235
◯ 236
◯ 237
◯ 238
◯ 239
◯ 240
◯ 241
◯ 242
◯ 243
◯ 244
◯ 245
◯ 246
◯ 247
◯ 248
◯ 249
◯ 250
◯ 251
◯ 252
◯ 253
◯ 254
◯ 255
◯ 256
◯ 257
◯ 258
◯ 259
◯ 260
◯ 261
◯ 262
◯ 263
◯ 264
◯ 265
◯ 266
◯ 267
◯ 268
◯ 270
◯ 271
◯ 272
◯ 273
◯ 274
◯ 275
◯ 276
◯ 277
◯ 278
◯ 279
◯ 280
◯ 281
◯ 282
◯ 283
◯ 284
◯ 285
◯ 286
◯ 287
◯ 288

◯ 289
◯ 290
◯ 291
◯ 292
◯ 293
◯ 294
◯ 295
◯ 296
◯ 297
◯ 298
◯ 299
◯ 300
◯ 301
◯ 302
◯ 303
◯ 304
◯ 305
◯ 306
◯ 307
◯ 308
◯ 309
◯ 310
◯ 311
◯ 312
◯ 313
◯ 314
◯ 315
◯ 316
◯ 317
◯ 318
◯ 319
◯ 320
◯ 321
◯ 322
◯ 323
◯ 324
◯ 325
◯ 326
◯ 327
◯ 328
◯ 329
◯ 330
◯ 331
◯ 332
◯ 333
◯ 334
◯ 335
◯ 336
◯ 337
◯ 338
◯ 339
◯ 340
◯ 341
◯ 342
◯ 344
◯ 346
◯ 347
◯ 349
◯ 350
◯ 351
◯ 352
◯ 354
◯ 355
◯ 356
◯ 357
◯ 358
◯ 359
◯ 360
◯ 361
◯ 362
◯ 363

◯ 364
◯ 365
◯ 366
◯ 367
◯ 368
◯ 369
◯ 370
◯ 371
◯ 372
◯ 373
◯ 374
◯ 375
◯ 376
◯ 377
◯ 378
◯ 379
◯ 380
◯ 381
◯ 382
◯ 383
◯ 384
◯ 385
◯ 386
◯ 387
◯ 388
◯ 389
◯ 390
◯ 391
◯ 392
◯ 393
◯ 394
◯ 395
◯ 396
◯ 397
◯ 398
◯ 399
◯ 400
◯ 401
◯ 402
◯ 403
◯ 404
◯ 405
◯ 406
◯ 407
◯ 408
◯ 409
◯ 410
◯ 411
◯ 412
◯ 413
◯ 414
◯ 415
◯ 416
◯ 417
◯ 418
◯ 420
◯ 421
◯ 422
◯ 423
◯ 424
◯ 425
◯ 426
◯ 427
◯ 430
◯ 431
◯ 432
◯ 433
◯ 434
◯ 435
◯ 436
◯ 437

○ 438
○ 439
○ 440
○ 441
○ 442
○ 443
○ 444
○ 445
○ 446
○ 447
○ 448
○ 449
○ 450
○ 451
○ 452
○ 453
○ 454
○ 455
○ 456
○ 457
○ 458
○ 459
○ 460
○ 461
○ 462
○ 463
○ 464
○ 465
○ 466
○ 467
○ 468
○ 469
○ 470
○ 471
○ 472
○ 473
○ 474
○ 475
○ 476
○ 477
○ 478
○ 479
○ 480
○ 481
○ 482
○ 483
○ 484
○ 485
○ 486
○ 487
○ 488
○ 489
○ 490
○ 491
○ 492
○ 493
○ 494
○ 495
○ 496
○ 497
○ 498
○ 499
○ 500
○ 501
○ 502
○ 503
○ 504
○ 505
○ 506
○ 507
○ 508

○ 509
○ 510
○ 511
○ 512
○ 513
○ 514
○ 515
○ 516
○ 520
○ 521
○ 522
○ 523
○ 524
○ 525
○ 526
○ 527
○ 528
○ 530
○ 531
○ 532
○ 534
○ 535
○ 537
○ 538
○ 539
○ 540
○ 541
○ 542
○ 543
○ 544
○ 545
○ 546
○ 547
○ 548
○ 549
○ 550
○ 551
○ 553
○ 554
○ 555
○ 556
○ 557
○ 558
○ 559
○ 560
○ 561
○ 562
○ 563
○ 564
○ 565
○ 566
○ 567
○ 569
○ 570
○ 571
○ 572
○ 573
○ 574
○ 575
○ 576
○ 577
○ 580
○ 581
○ 582
○ 583
○ 584
○ 585
○ 586
○ 587
○ 588
○ 590

◯ 591
◯ 592
◯ 593
◯ 594
◯ 595
◯ 596
◯ 597
◯ 598
◯ 599
◯ 600
◯ 601
◯ 602
◯ 603
◯ 604
◯ 605
◯ 606
◯ 607
◯ 608
◯ 609
◯ 610
◯ 611
◯ 612
◯ 613
◯ 614
◯ 615
◯ 616
◯ 617
◯ 618
◯ 619
◯ 620
◯ 622
◯ 623
◯ 624
◯ 625
◯ 626
◯ 627
◯ 628
◯ 629
◯ 630
◯ 631
◯ 633
◯ 634
◯ 635
◯ 636
◯ 637
◯ 638
◯ 639
◯ 640
◯ 641
◯ 644
◯ 645
◯ 646
◯ 647
◯ 648
◯ 649
◯ 650
◯ 651
◯ 652
◯ 653
◯ 654
◯ 655
◯ 656
◯ 657
◯ 658
◯ 660
◯ 661
◯ 662
◯ 664
◯ 665
◯ 666
◯ 667

◯ 668
◯ 669
◯ 670
◯ 671
◯ 672
◯ 673
◯ 674
◯ 675
◯ 676
◯ 677
◯ 678
◯ 679
◯ 680
◯ 681
◯ 683
◯ 684
◯ 685
◯ 686
◯ 687
◯ 688
◯ 689
◯ 690
◯ 691
◯ 692
◯ 693
◯ 700
◯ 701
◯ 703
◯ 704
◯ 705
◯ 706
◯ 707
◯ 708
◯ 710
◯ 711
◯ 712
◯ 713
◯ 714
◯ 716
◯ 717
◯ 718
◯ 719
◯ 720
◯ 721
◯ 722
◯ 723
◯ 724
◯ 725
◯ 726
◯ 727
◯ 728
◯ 729
◯ 730
◯ 731
◯ 733
◯ 734
◯ 735
◯ 736
◯ 737
◯ 738
◯ 739
◯ 740
◯ 741
◯ 743
◯ 744
◯ 745
◯ 746
◯ 747
◯ 748
◯ 749
◯ 750

○ 751
○ 752
○ 753
○ 754
○ 755
○ 756
○ 757
○ 758
○ 759
○ 760
○ 761
○ 762
○ 763
○ 764
○ 765
○ 766
○ 767
○ 768
○ 769
○ 770
○ 772
○ 773
○ 774
○ 775
○ 776
○ 777
○ 778
○ 779
○ 780
○ 781
○ 782
○ 783
○ 784
○ 785
○ 786
○ 787
○ 788
○ 789
○ 790
○ 791
○ 792
○ 793
○ 794
○ 795
○ 796
○ 797
○ 798
○ 799
○ 800
○ 801
○ 802
○ 803
○ 804
○ 805
○ 806
○ 807
○ 808
○ 809
○ 810
○ 811
○ 812
○ 813
○ 814
○ 815
○ 816
○ 820
○ 821
○ 822
○ 823
○ 824
○ 825

○ 826
○ 827
○ 828
○ 829
○ 830
○ 831
○ 832
○ 833
○ 834
○ 835
○ 836
○ 837
○ 838
○ 840
○ 841
○ 842
○ 843
○ 844
○ 845
○ 846
○ 847
○ 850
○ 851
○ 852
○ 853
○ 855
○ 856
○ 857
○ 859
○ 860
○ 863
○ 864
○ 865
○ 870
○ 871
○ 873
○ 874
○ 875
○ 876
○ 877
○ 878
○ 879
○ 880
○ 881
○ 882
○ 883
○ 884
○ 885
○ 888
○ 889
○ 890
○ 891
○ 893
○ 894
○ 895
○ 897
○ 898
○ 900
○ 901
○ 902
○ 903
○ 904
○ 905
○ 906
○ 907
○ 908
○ 910
○ 911
○ 912
○ 913
○ 914

◯ 915
◯ 916
◯ 917
◯ 918
◯ 919
◯ 920
◯ 921
◯ 922
◯ 923
◯ 924
◯ 925
◯ 926
◯ 927
◯ 928
◯ 930
◯ 931
◯ 932
◯ 933
◯ 934
◯ 935
◯ 936
◯ 937
◯ 938
◯ 939
◯ 940
◯ 941
◯ 942
◯ 943
◯ 944
◯ 945
◯ 946
◯ 947
◯ 948
◯ 949
◯ 950
◯ 951
◯ 952
◯ 953
◯ 954
◯ 955
◯ 956
◯ 957
◯ 958
◯ 959
◯ 960
◯ 961
◯ 962
◯ 963
◯ 964
◯ 965
◯ 966
◯ 967
◯ 968
◯ 969
◯ 970
◯ 971
◯ 972
◯ 973
◯ 974
◯ 975
◯ 976
◯ 977
◯ 978
◯ 979
◯ 980
◯ 981
◯ 982
◯ 983
◯ 984
◯ 985
◯ 986

◯ 988
◯ 989
◯ 990
◯ 991
◯ 992
◯ 993
◯ 994
◯ 995
◯ 996
◯ 997
◯ 998
◯ 999
◯ Missing

Hospital Code List (use for finding your hospital code, table of contents is clickable)

[Attachment: "RECOVER-CDC All sites and Codes.xlsx"]

Hospital Code

- ◯ BE01: Beaumont Health (William Beaumont Hospital) - Royal Oak
- ◯ BE02: Beaumont Health (William Beaumont Hospital) - Troy
- ◯ BE03: Beaumont Health (William Beaumont Hospital) - Dearborn
- ◯ BE04: Beaumont Health (William Beaumont Hospital) - Farmington Hills
- ◯ BE05: Beaumont Health (William Beaumont Hospital) - Grosse Pointe
- ◯ BE06: Beaumont Health (William Beaumont Hospital) - Taylor
- ◯ BE07: Beaumont Health (William Beaumont Hospital) - Trenton
- ◯ BE08: Beaumont Health (William Beaumont Hospital) - Wayne
- ◯ GW01: George Washington University (Medical Faculty Associates) - George Washington University Hospital
- ◯ GW02: George Washington University (Medical Faculty Associates) - United Medical Center
- ◯ GW03: George Washington University (Medical Faculty Associates) - Inova Fairfax
- ◯ GW04: George Washington University (Medical Faculty Associates) - Washington Hospital Center
- ◯ GW05: George Washington University (Medical Faculty Associates) - Georgetown University
- ◯ GW06: George Washington University (Medical Faculty Associates) - Southern Maryland
- ◯ HM01: Hennepin County Medical Center - Hennepin County Medical Center
- ◯ IM01: Intermountain Medical Center (IHC Health Services, Inc) - Intermountain Medical Center
- ◯ IM02: Intermountain Medical Center (IHC Health Services, Inc) - Utah Valley
- ◯ IM03: Intermountain Medical Center (IHC Health Services, Inc) - Primary Children's
- ◯ IM04: Intermountain Medical Center (IHC Health Services, Inc) - LDS Hospital
- ◯ IM05: Intermountain Medical Center (IHC Health Services, Inc) - Dixie Regional Medical Center
- ◯ IM06: Intermountain Medical Center (IHC Health Services, Inc) - Logan Regional
- ◯ IM07: Intermountain Medical Center (IHC Health Services, Inc) - Riverton
- ◯ IM08: Intermountain Medical Center (IHC Health Services, Inc) - American Fork
- ◯ IM09: Intermountain Medical Center (IHC Health Services, Inc) - Alta View
- ◯ IM10: Intermountain Medical Center (IHC Health Services, Inc) - Cedar City
- ◯ IM11: Intermountain Medical Center (IHC Health Services, Inc) - Park City
- ◯ IM12: Intermountain Medical Center (IHC Health Services, Inc) - Sevier Valley
- ◯ IM13: Intermountain Medical Center (IHC Health Services, Inc) - Cassia Regional
- ◯ IM14: Intermountain Medical Center (IHC Health Services, Inc) - Orem Community
- ◯ IM15: Intermountain Medical Center (IHC Health Services, Inc) - Fillmore Community
- ◯ IM16: Intermountain Medical Center (IHC Health Services, Inc) - Heber Valley
- ◯ IM17: Intermountain Medical Center (IHC Health Services, Inc) - Delta Community
- ◯ IM18: Intermountain Medical Center (IHC Health Services, Inc) - Sanpete Valley
- ◯ IM19: Intermountain Medical Center (IHC Health Services, Inc) - Bear River Valley

◯ IM20: Intermountain Medical Center (IHC Health Services, Inc) - Garfield Memorial
◯ IM21: Intermountain Medical Center (IHC Health Services, Inc) - Layton
◯ IM22: Intermountain Medical Center (IHC Health Services, Inc) - McKay-Dee Hospital
◯ IM23: Intermountain Medical Center (IHC Health Services, Inc) - Spanish Fork
◯ MG01: Massachusetts General Hospital (The General Hospital Corporation) - Massachusetts General Hospital
◯ MS01: Icahn School of Medicine at Mount Sinai - Mount Sinai Hospital
◯ MS02: Icahn School of Medicine at Mount Sinai - Mount Sinai Brooklyn
◯ MS03: Icahn School of Medicine at Mount Sinai - Mount Sinai Morningside
◯ MS04: Icahn School of Medicine at Mount Sinai - Mount Sinai West
◯ MS05: Icahn School of Medicine at Mount Sinai - Mount Sinai Queens
◯ MS06: Icahn School of Medicine at Mount Sinai - Mount Sinai Beth Israel
◯ MS07: Icahn School of Medicine at Mount Sinai - Elmhurst Hospital
◯ OH01: Oregon Health & Science University - OHSU Adult ED
◯ OH02: Oregon Health & Science University - OHSU Pediatric ED
◯ OH03: Oregon Health & Science University - Columbia Memorial ED
◯ OH04: Oregon Health & Science University - Hillsboro ED
◯ OH05: Oregon Health & Science University - Adventist ED
◯ PS01: Penn State Hershey Medical Center - Penn State Hershey Medical Center
◯ BU01: Rhode Island Hospital_Brown University - Rhode Island Hospital
◯ BU02: Rhode Island Hospital_Brown University - Miriam Hospital
◯ BU03: Rhode Island Hospital_Brown University - Newport Hospital
◯ BU04: Rhode Island Hospital_Brown University - Hasbro Children's Hospital
◯ CO01: Centura Health System Colorado - Avista Adventist Hospital
◯ CO02: Centura Health System Colorado - St. Anthony Hospital
◯ CO03: Centura Health System Colorado - St. Anthony North Hospital
◯ CO04: Centura Health System Colorado - St. Anthony Summit Medical Center
◯ CO05: Centura Health System Colorado - Littleton Adventist Hospital
◯ CO06: Centura Health System Colorado - Parker Adventist Hospital
◯ CO07: Centura Health System Colorado - Porter Adventists Hospital
◯ CO08: Centura Health System Colorado - OrthoColorado Hospital
◯ CO09: Centura Health System Colorado - Penrose Hospital
◯ CO10: Centura Health System Colorado - St. Francis Medical Center
◯ CO11: Centura Health System Colorado - St. Mary-Corwin Medical Center
◯ CO12: Centura Health System Colorado - St. Thomas More Hospital
◯ CO13: Centura Health System Colorado - St. Catherine Hospital

◯ CO14: Centura Health System Colorado - Mercy Hospital
◯ CO15: Centura Health System Colorado - Longmont United Hospital
◯ CO16: Centura Health System Colorado - Castle Rock Adventist Hospital
◯ CO17: Centura Health System Colorado - Bob Wilson Memorial Hospital
◯ CO18: Centura Health System Colorado - Other Sites not Listed
◯ OS01: The Ohio State University - University Hospital
◯ OS02: The Ohio State University - East Hospital
◯ IU01: Trustees of Indiana University - IU Health Methodist Hospital
◯ IU02: Trustees of Indiana University - IU Health Bloomington Hospital
◯ IU03: Trustees of Indiana University - IU Health Bedford Hospital
◯ IU04: Trustees of Indiana University - IU Health Morgan Hospital
◯ IU05: Trustees of Indiana University - IU Health White Memorial Hospital
◯ IU06: Trustees of Indiana University - Sydney & Lois Eskenazi Hospital
◯ IU07: Trustees of Indiana University - IU Health Jay Hospital
◯ IU08: Trustees of Indiana University - IU Health University Hospital
◯ IU09: Trustees of Indiana University - Riley Hospital for Children
◯ IU10: Trustees of Indiana University - IU Health North Hospital
◯ IU11: Trustees of Indiana University - IU Health West Hospital
◯ IU12: Trustees of Indiana University - IU Health Arnett Hospital
◯ IU13: Trustees of Indiana University - IU Health Ball Memorial Hospital
◯ IU14: Trustees of Indiana University - IU Health Blackford Hospital
◯ IU15: Trustees of Indiana University - IU Health La Porte Hospital
◯ IU16: Trustees of Indiana University - IU Health Paoli Hospital
◯ IU17: Trustees of Indiana University - IU Health Saxony Hospital
◯ IU18: Trustees of Indiana University - IU Health Starke Hospital
◯ IU19: Trustees of Indiana University - IU Health Tipton Hospital
◯ IU20: Trustees of Indiana University - IU Health Frankfort Hospital
◯ SD01: University of California San Diego - UCSD Hillcrest Medical Center
◯ SD02: University of California San Diego - UCSD La Jolla Medical Center
◯ CM01: University of Chicago - University of Chicago Medicine
◯ UC01: University of Colorado - All UC Health Hospitals
◯ UI01: University of Iowa - University of Iowa Hospitals and Clinics
◯ MI01: University of Mississippi - University Hospital
◯ MI02: University of Mississippi - Wallace Conerly Critical Care Hospital
◯ MI03: University of Mississippi - Winfred L. Wiser Hospital for Women and Infants
◯ MI04: University of Mississippi - Blair E. Batson Children's Hospital

- ◯ MI05: University of Mississippi - UMMC Holmes County
- ◯ MI06: University of Mississippi - UMMC Grenada
- ◯ UU01: University of Utah Health Sciences Center - University of Utah Hospital
- ◯ UU02: University of Utah Health Sciences Center - South Jordan Health Center
- ◯ UW01: University of Wisconsin - Madison- System (The Board of Regents of the) - University of Wisconsin Hospital
- ◯ UW02: University of Wisconsin - Madison- System (The Board of Regents of the) - The American Center
- ◯ LS01: University Medical Center New Orleans - University Medical Center New Orleans
- ◯ LS02: University Medical Center New Orleans - Touro Infirmary Hospital
- ◯ LS03: University Medical Center New Orleans - West Jefferson Medical Center
- ◯ LS04: University Medical Center New Orleans - New Orleans East Hospital
- ◯ UT01: UT Southwestern - Clements University Hospital
- ◯ UT02: UT Southwestern - Parkland Hospital
- ◯ WU01: Washington University in St. Louis - Barnes-Jewish Hospital
- ◯ WU02: Washington University in St. Louis - St. Louis Children's Hospital
- ◯ WS01: Wayne State University - Detroit Receiving Hospital
- ◯ WS02: Wayne State University - Harper University Hospital
- ◯ WS03: Wayne State University - Sinai Grace Hospital
- ◯ WS04: Wayne State University - Huron Valley-Sinai Hospital
- ◯ WV01: West Virginia University - Ruby Memorial Hospital

Total emergency department volume during the week of survey

____

Method of arrival

____
(walk-in, private vehicle, EMS etc.)

Vital Signs at index visit

Vital Signs Guidance Document

Vital Signs Guidelines CHANGE AS OF 11-10-2021: VITAL SIGN BOUNDARY RANGES HAVE BEEN REMOVED; IMPORT WHATEVER IS CHARTED

1. Except for lowest pulse ox, use vital signs recorded at triage during the index visit in the emergency department. The index visit is the first visit in the week prior to data collection

2. Use discretion if a first vital sign appears erroneous, or is missing. If this is the case please use a more appropriate value measured later. An example would be an extreme value.

3. Expected ranges are listed for each vital. If you have a data point outside of the expected range please record the closest possible value. Example: Heart Rate = 35, record 39

4. Please note that the decimal places for the data entered must correspond to the expected range.

Arrived in cardiac arrest (if uncertain select 'No')	◯ Yes<br>◯ No
First heart rate recorded (0 to 250 beats per min)	______________<br>(beats per min)
First respiratory rate recorded (0 to 80 breaths per min)	______________<br>(breaths per min)
First O2 Saturation recorded (35 - 100%)	______________<br>(%)
Lowest O2 saturation while in ED (35 to 100%)	______________<br>(%)
First temperature recorded in degrees F (leave blank if site uses C)	______________
First temperature in celsius (leave blank if site uses F)	______________<br>(degrees C)
First Systolic Blood Pressure (-1 to 300 mmHg)	______________<br>(mmHg)

First Diastolic Blood Pressure (-1 to 300 mmHg)

____
(mmHg)

Demographics, vaccination status and ICD codes

Demographics Guidance Document	
Biological Sex	◯ Female<br>◯ Male<br>◯ Other<br>◯ Unknown<br>(as documented in EMR; order of preference: 1. Gender at birth, 2. Legal gender, 3. stated gender)
Race	◯ American Indian or Alaska Native<br>◯ Asian<br>◯ Black or African American<br>◯ Native Hawaiian or Other Pacific Islander<br>◯ White<br>◯ More than one race<br>◯ Unknown/Other<br>(These are official federal designations:"American Indian or Alaska Native. A person having origins in any of the original peoples of North and South America (including Central America), and who maintains tribal affiliation or community attachment. Asian. A person having origins in any of the original peoples of the Far East, Southeast Asia, or the Indian subcontinent including, for example, Cambodia, China, India, Japan, Korea, Malaysia, Pakistan, the Philippine Islands, Thailand, and Vietnam. Black or African American. A person having origins in any of the black racial groups of Africa. Terms such as ""Haitian"" or ""Negro"" can be used in addition to ""Black or African American."" Native Hawaiian or Other Pacific Islander. A person having origins in any of the original peoples of Hawaii, Guam, Samoa, or other Pacific Islands. White. A person having origins in any of the original peoples of Europe, the Middle East, or North Africa.")
Ethnicity	◯ Hispanic or Latino<br>◯ Not Hispanic or Latino<br>◯ Unknown<br>(Report the most appropriate, either stated ethnicity or ethnic group at your discretion. Ethnicity as asked is required for federally funded projects. Definition: Hispanic or Latino. A person of Cuban, Mexican, Puerto Rican, South or Central American, or other Spanish culture or origin, regardless of race. The term, "Spanish origin," can be used in addition to "Hispanic or Latino.")
Choose type of health insurance the patient had at the index emergency department visit	◯ Private or commercial health insurance (includes HMOs, PPOs, POS, managed care, fee for service)<br>◯ Medicaid<br>◯ Medicare<br>◯ Medicaid and Medicare<br>◯ Worker's compensation<br>◯ No health insurance (self-pay)<br>◯ Unknown

Age at time of visit (if < 1 year complete next queston)	__________________________________<br>(years)
If age < 1 year how many months old	◯ 0<br>◯ 1<br>◯ 2<br>◯ 3<br>◯ 4<br>◯ 5<br>◯ 6<br>◯ 7<br>◯ 8<br>◯ 9<br>◯ 10<br>◯ 11<br>(Round up to nearest month)
Import the text of the chief complaint	__________________________________<br>(This is the typed chief complaint but is not used for the case definition, which comes from the ICD code at discharge or admission from ED.)
Vaccination status for seasonal influenza	◯ Documented as unvaccinated against influenza during the current season (starts July 1 each year)<br>◯ Documented as vaccinated against influenza during the current season (starts July 1 each year)<br>◯ Influenza vaccination status unknown<br>(Vaccine from the fall "times out" on the following July 1)
COVID-19 Vaccination Status	◯ Vaccination status is not documented<br>◯ Vaccination status documented and confirms no vaccination<br>◯ Vaccination documented as positive (the patient was vaccinated) and whether fully or partially vaccinated is not documented<br>◯ Vaccination was given and known to be one dose of a two-dose regimen<br>◯ Full vaccination documented
COVID-19 Vaccine Manufacturer	◯ Manufacturer unknown<br>◯ Johnson & Johnson<br>◯ Pfizer<br>◯ Moderna<br>◯ More than one manufacturer<br>◯ Other vaccine (outside of US)
Import list of medications taken at home at time of the index ED visit	__________________________________<br>(Required per contract, comma separated)

Past Medical History at index visit

Past Medical History Data Guidelines

This section is based upon charted social and past medical history. "NA" is only an option for smoking, EtOH and opioid use disorder. Otherwise, if the past medical condition is not charted (e.g., diabetes mellitus) then default to no as the answer.

Smoker (Current smoker regardless of number of cigarettes; includes vaping, cigars or pipe smoking)
(Leave blank if not documented)

◯ Yes ◯ No ◯ NA (No information is available in the medical record)

Alcohol use

◯ Yes ◯ No ◯ NA (No information is available in the medical record)

Opioid use disorder
(ICD: F11. 90)

◯ Yes ◯ No ◯ NA (Information not available in the medical record)

Diabetes Mellitus	◯ Yes<br>◯ No<br>(ICD: E8-E13)
Systemic Hypertension (Regardless of treatment status; can also be diagnosied by diastolic BP >110 mm Hg in ED)	◯ Yes<br>◯ No<br>(ICD: I10X, I11X or I15X)
Prior ischemic heart disease (myocardial infarction, or any coronary revascularization including stent or CABG)	◯ Yes<br>◯ No<br>(I 24X or I 25X)

Obesity (by diagnosis, provider interpretation or body mass index >35 Kg/m2)

◯ Yes ◯ No

Weight	________________________
Height	________________________
Weight Units	◯ Pounds<br>◯ Ounces<br>◯ Kilograms<br>◯ Grams
Height Units	◯ Feet<br>◯ Inches<br>◯ Meters<br>◯ Centimeters

Height in meters

____

Weight in kilos

____

BMI - Calculated within REDCap

____
(This field DOES NOT get imported)

BMI - Reported in EMR

____

Hyperlipidemias (hyperlipidemia of any type, includes currently taking a statin)
(ICD: E78X)

◯ Yes ◯ No

Heart failure (either systolic or ejection fraction preserved)
(ICD: I50X or I 11.0)

◯ Yes ◯ No

Atrial fibrillation?

◯ Yes
◯ No
(ICD: I 48X)

Cancer (active or in remission)
(ICD: C00-D49)

◯ Yes ◯ No

Cancer now in remission

◯ Yes ◯ No ◯ Unknown

Chronic obstructive pulmonary disease (by history)
(ICD: J44X)

◯ Yes ◯ No

Asthma
(ICD: J45X)

◯ Yes ◯ No

Prior pulmonary embolism

◯ Yes
◯ No
(ICD: I26X)

Prior deep vein thrombosis

◯ Yes
◯ No
(ICD: I82X)

Other lung disease (pulmonary fibrosis, cystic fibrosis, bronchiectasis, pulmonary hypertension)

◯ Yes ◯ No

Organ transplantation (any organ)
(Z94X)

◯ Yes ◯ No

HIV
(B20)

◯ Yes ◯ No ◯ Unable to collect per institution

Medications at index visit

Instructions:
If medication is not documented (neither charted as present nor absent) then default to answer "no" or "none"

Current Medications Guidance Document

Ibuprofen on medication list?	◯ Yes<br>◯ No<br>(in last 24 hours)
Acetaminophen on medication list?	◯ Yes<br>◯ No<br>(in last 24 hours)
Aspirin on medication list?	◯ Yes<br>◯ No<br>(in last 24 hours)
Statin on medication list?	◯ None<br>◯ Atorvastatin (Lipitor)<br>◯ Fluvastatin (Lescol, Lescol XL)<br>◯ Lovastatin (Mevacor, Altoprev)<br>◯ Pravastatin (Pravachol)<br>◯ Rosuvastatin (Crestor)<br>◯ Simvastatin (Zocor)<br>◯ Pitavastatin (Livalo)
ACE inhibitor on medication list?	◯ None<br>◯ Benazepril (Lotensin, Lotensin Hct)<br>◯ Captopril (Capoten)<br>◯ Enalapril (Vasotec)<br>◯ Fosinopril (Monopril)<br>◯ Lisinopril (Prinivil, Zestril)<br>◯ Moexipril (Univasc)<br>◯ Perindopril (Aceon)<br>◯ Quinapril (Accupril)<br>◯ Ramipril (Altace)<br>◯ Trandolapril (Mavik)
Angiotensin receptor blocker on medication list?	◯ None<br>◯ Azilsartan (Edarbi)<br>◯ Candesartan (Atacand)<br>◯ Eprosartan<br>◯ Irbesartan (Avapro)<br>◯ Losartan (Cozaar)<br>◯ Olmesartan (Benicar)<br>◯ Telmisartan (Micardis)<br>◯ Valsartan (Diovan)
Thiazolidinedione on medication list?	◯ None<br>◯ Pioglizazone (actos)<br>◯ Rosiglitazone (avandia)

Anticoagulants on medication list	◯ Apixaban<br>◯ Dabigatran<br>◯ Edoxaban<br>◯ Low molecular weight heparin<br>◯ Rivaroxaban<br>◯ Unfractionated heparin<br>◯ Warfarin<br>◯ None

Test Results from index ED visit

Test Results Data Guidelines for ED visit

1. These data are populated on the first download 2. All lab values refer to the first done. If no evidence can be found in EMR of the test result, then please check no, or leave value blank, as appropriate. For example, we gave the option of populating up to two COVID, influenza and viral panel results, recognizing that it will be very unlikely that a patient gets two tests in one week, but this is possible. 3. Expected ranges are listed for each laboratory test. If you have a data point outside of the expected range please record the closest possible value. Example: if WBC = 95.0 x10^cells/uL, record 91.0 4. Please note that the decimal places for the data entered must correspond to the expected range. 5. These can be imported.

First SARS-COV-2 test results	◯ Not done<br>◯ Negative<br>◯ Positive<br>◯ Indeterminate<br>◯ Result unknown<br>(If a patient had at least one SARS-CoV-2 test at index, report the earliest result here. If a patient had no SARS-CoV-2 test at the index visit, but then had several tests within the week of query, report the test closest to the index visit.)
Date of first SARS-CoV-2 test result (if done)	______________________
Second SARS-COV-2 test results	◯ Not done<br>◯ Negative<br>◯ Positive<br>◯ Indeterminate<br>◯ Result unknown<br>(If a patient had at least two SARS-CoV-2 tests at the index visit, report the second test result here. If a patient had no SARS-CoV-2 test at the index visit, but then had several tests within the week of query, report the test immediately following the one report for "First SARS-CoV-2 test results".)
Date of second SARS-CoV-2 test result (if done)	______________________
First Influenza A (Type Unknown) test result	◯ Not done<br>◯ Negative<br>◯ Positive
First Influenza A (H1) test result	◯ Not done<br>◯ Negative<br>◯ Positive
First Influenza A (H3) test result	◯ Not done<br>◯ Negative<br>◯ Positive

First Influenza A (H5) test result	◯ Not done<br>◯ Negative<br>◯ Positive
First Influenza B test result	◯ Not done<br>◯ Negative<br>◯ Positive
Date of first influenza test (if done)	__________________________________
Second Influenza A (Type Unknown) test result	◯ Not done<br>◯ Negative<br>◯ Positive
Second Influenza A (H1) test result	◯ Not done<br>◯ Negative<br>◯ Positive
Second Influenza A (H3) test result	◯ Not done<br>◯ Negative<br>◯ Positive
Second Influenza A (H5) test result	◯ Not done<br>◯ Negative<br>◯ Positive
Second Influenza B test result	◯ Not done<br>◯ Negative<br>◯ Positive
Date of second influenza test (if done)	__________________________________
Other viral test within 24 hours of SARS-COV-2 testing (First test)?	◯ Yes<br>◯ No<br>(This and the subsequent questions will typically come from a viral panel or other multiplex platform)
Date of first "other" respiratory viral testing	__________________________________
Respiratory adenovirus	◯ Positive<br>◯ Negative<br>◯ Not Done
Other Coronavirus - Type Unknown	◯ Positive<br>◯ Negative<br>◯ Not Done
Other Coronavirus - HKU1	◯ Positive<br>◯ Negative<br>◯ Not Done

Other Coronavirus - NL63	◯ Positive<br>◯ Negative<br>◯ Not Done
Other Coronavirus - 229E	◯ Positive<br>◯ Negative<br>◯ Not Done
Other Coronavirus - OC43	◯ Positive<br>◯ Negative<br>◯ Not Done
Respiratory syncytial virus (RSV) - Type A	◯ Positive<br>◯ Negative<br>◯ Not Done
Respiratory syncytial virus (RSV) - Type B	◯ Positive<br>◯ Negative<br>◯ Not Done
Respiratory syncytial virus (RSV) - Type Unknown	◯ Positive<br>◯ Negative<br>◯ Not Done
Human Metapneumovirus	◯ Positive<br>◯ Negative<br>◯ Not Done
Human rhinovirus/enterovirus	◯ Positive<br>◯ Negative<br>◯ Not Done
Parainfluenza - Type Unknown	◯ Positive<br>◯ Negative<br>◯ Not Done
Parainfluenza - Virus 1	◯ Positive<br>◯ Negative<br>◯ Not Done
Parainfluenza - Virus 2	◯ Positive<br>◯ Negative<br>◯ Not Done
Parainfluenza - Virus 3	◯ Positive<br>◯ Negative<br>◯ Not Done
Parainfluenza - Virus 4	◯ Positive<br>◯ Negative<br>◯ Not Done
Influenza result from viral panel - Type A	◯ Positive<br>◯ Negative<br>◯ Not Done

Influenza result from viral panel - Type A H1	◯ Positive<br>◯ Negative<br>◯ Not Done
Influenza result from viral panel - Type A H3	◯ Positive<br>◯ Negative<br>◯ Not Done
Influenza result from viral panel - Type B	◯ Positive<br>◯ Negative<br>◯ Not Done
Pertussis - B Parapertussis	◯ Positive<br>◯ Negative<br>◯ Not Done
Pertussis - B Pertussis	◯ Positive<br>◯ Negative<br>◯ Not Done
Other Virus	◯ Positive<br>◯ Negative<br>◯ Not Done
Name Other Virus	__________________________________
Other respiratory viral test within 24 hours of SARS-COV-2 testing (SECONDtest)?	◯ Yes<br>◯ No<br>(This and the subsequent questions will typically come from a viral panel)
Date of SECOND other viral testing	__________________________________
Respiratory adenovirus (second test)	◯ Positive<br>◯ Negative<br>◯ Not Done
Other Coronavirus (second test) - Type Unknown	◯ Positive<br>◯ Negative<br>◯ Not Done
Other Coronavirus (second test) - HKU1	◯ Positive<br>◯ Negative<br>◯ Not Done
Other Coronavirus (second test) - NL63	◯ Positive<br>◯ Negative<br>◯ Not Done
Other Coronavirus (second test) - 229E	◯ Positive<br>◯ Negative<br>◯ Not Done

Other Coronavirus (second test) - OC43	◯ Positive<br>◯ Negative<br>◯ Not Done
Respiratory syncytial virus (RSV) - Type A (second test)	◯ Positive<br>◯ Negative<br>◯ Not Done
Respiratory syncytial virus (RSV) - Type B (second test)	◯ Positive<br>◯ Negative<br>◯ Not Done
Respiratory syncytial virus (RSV) - Type Unknown (second test)	◯ Positive<br>◯ Negative<br>◯ Not Done
Human Metapneumovirus (second test)	◯ Positive<br>◯ Negative<br>◯ Not Done
Human rhinovirus/enterorvirus (second test)	◯ Positive<br>◯ Negative<br>◯ Not Done
Parainfluenza (second test) - Type Unknown	◯ Positive<br>◯ Negative<br>◯ Not Done
Parainfluenza (second test) - Virus 1	◯ Positive<br>◯ Negative<br>◯ Not Done
Parainfluenza (second test) - Virus 2	◯ Positive<br>◯ Negative<br>◯ Not Done
Parainfluenza (second test) - Virus 3	◯ Positive<br>◯ Negative<br>◯ Not Done
Parainfluenza (second test) - Virus 4	◯ Positive<br>◯ Negative<br>◯ Not Done
Influenza result from viral panel (second test) - Type A	◯ Positive<br>◯ Negative<br>◯ Not Done
Influenza result from viral panel (second test) - Type A H1	◯ Positive<br>◯ Negative<br>◯ Not Done
Influenza result from viral panel (second test) - Type A H3	◯ Positive<br>◯ Negative<br>◯ Not Done

Influenza result from viral panel (second test) - Type B	◯ Positive<br>◯ Negative<br>◯ Not Done
Pertussis (second test) - B Parapertussis	◯ Positive<br>◯ Negative<br>◯ Not Done
Pertussis (second test) - B Pertussis	◯ Positive<br>◯ Negative<br>◯ Not Done
Chest radiograph done?	◯ Yes<br>◯ No<br>(If a patient had at least one chest radiograph at index, report 'Yes' here. If a patient had no chest radiograph at the index visit, but then had several within the week of query, report the 'Yes'.)
Chest radiograph findings, import conclusion or findings section	__________________________________<br>(Use radiologist read. If a patient had at least one chest radiograph at index, report the findings of the earliest radiograph here. If a patient had no chest radiograph at the index visit, but then had several within the week of query, report the findings of the radiograph closest to the index visit.)
Chest Radiograph Findings	◯ Normal<br>◯ Abnormal<br>◯ Not Done
Chest computed tomography done?	◯ Yes<br>◯ No
Venous ultrasound results (import findings or conclusions)	__________________________________<br>(If a patient had at least one venous ultrasound at index, report the earlier result here. If a patient had no venous US at the index visit, but then had several within the week of query, report the result closest to the index visit.)
Chest computed tomography findings, import conclusions or findings	__________________________________<br>(Use radiologist read. If a patient had at least one chest CT at index, report the earliest result here. If a patient had no chest CT at the index visit, but then had several within the week of query, report the result closest to the index visit.)

Chest computed tomography Findings	◯ Normal<br>◯ Abnormal<br>◯ Not Done
Pregnancy test (urine or serum) positive	◯ Yes<br>◯ No<br>◯ Not Done
WBC (0.0 - 90.0 103/uL)	____________________<br>(103/uL)
Lymphocyte count (0.0 - 60.0 103 cells/uL)	____________________<br>(This is a cell count, not a %, report in 103 cells/uL which is the same as 109 cells/L)
Hemoglobin (2.0 - 22.0 g/dL)	____________________<br>(g/dL)
Platelets (10 - 1M 103 cells/uL)	____________________<br>(103 cells/uL)
Aspartate transaminase (1 - 5,000 IU)	____________________<br>(IU)
Alanine transaminase (0 - 5,000 IU)	____________________<br>(IU)
Total bilirubin (0.0 - 20.0 mg/dL)	____________________<br>(mg/dL)
Albumin (1.0 - 7.0 g/dL)	____________________<br>(g/dL)
Alkaline Phosphatase (10 - 1,000 IU)	____________________<br>(IU)
Na (100 - 180 mEq/L)	____________________<br>(mEq/L)
Cl (70 - 160 mEq/L)	____________________<br>(mEq/L)
K (1.0 -15.0 mEq/L)	____________________<br>(mEq/L)

CO2 (1 - 40 mEq/L)

______________________ (mEq/L)

BUN (1 -150 mg/dL)

______________________ (mg/dL)

Cr (0.10 - 20.00 mg/dL)

______________________ (mg/dL)

Glucose (10 - 2,500 mg/dL)

______________________ (mg/dL)

First Documented Troponin

- ◯ Standard Assay
- ◯ High-sensitivity Assay
- ◯ No troponin recorded

First troponin (0.00 - 100.00 ng/mL)

______________________ (ng/mL - if your value has a < in front of it please record the next lowest significant number (i.e. < 0.03 = 0.02))

First High-Sensitivity Troponin (0.0 - 100,000.0 pg/mL)

______________________ (pg/mL - if your value has a < in front of it please record the next lowest significant number (i.e. < 0.03 = 0.02))

Troponin Type

- ◯ I
- ◯ T

d-dimer (200 - 100,000 ng/mL fibrinogen equivalent units)

______________________ (If the laboratory result is D-dimer units (typical threshold of abnormal is 200-250 ng/mL), please multiply times two . When the threshold for abnormal is 500 ng/mL for DVT or PE, this always means the machine is reporting FEUs)

C Reactive protein (0.1 - 40.0 mg/dL)

______________________ (mg/dL)

Procalcitonin (0.00 - 100.00 mcg/L)

______________________ (mcg/L)

Outcomes from index ED visit

Outcomes Data Guidlelines 1. These are outcomes that are available for qualifying ARI cases from the week prior	
Was the patient kept in, or designated as, "Observation Status" (< 24 hour stay) during the index visit	◯ Yes<br>◯ No
Was patient ADMITTED to the hospital on the index visit?	◯ Yes<br>◯ No
If patient admitted, please specific admission location in hospital	◯ Initial admit observation<br>◯ Initial admit regular or monitored floor<br>◯ Initial admit stepdown or progressive care<br>◯ Initial admit intensive care unit<br>(If patient admitted more than once in 30 days after testing, answer questions for admission closest in time to the SARS-CoV-2 testing used to locate patient)
Import all ICD codes coded from index ED visit	___________________________________<br>(This question may or may not have the same data as the be question on ICD codes in the demographics form)
Import all CPT codes from index ED visit	___________________________________<br>(comma separated)
Import all medications administered during the index ED visit.	___________________________________<br>(comma separated)

Index Metadata

Timestamp of Initial Index EHR Report ________________________________

Timestamp of Most Recent Index EHR Report ________________________________

Index Record Created
NOTE: SITES DO NOT NEED TO POPULATE THIS FIELD ________________________________
(NOTE: SITES DO NOT NEED TO POPULATE THIS FIELD)

Index Record Updated
NOTE: SITES DO NOT NEED TO POPULATE THIS FIELD ________________________________
(NOTE: SITES DO NOT NEED TO POPULATE THIS FIELD)

Index Source File
NOTE: SITES DO NOT NEED TO POPULATE THIS FIELD ________________________________
(NOTE: SITES DO NOT NEED TO POPULATE THIS FIELD)

Index Update File
NOTE: SITES DO NOT NEED TO POPULATE THIS FIELD ________________________________
(NOTE: SITES DO NOT NEED TO POPULATE THIS FIELD)

Test Results from 30 day outcomes

Test Results Data Guidelines

1. All lab values refer to the first done. If no evidence can be found in EMR of the test result, then please check no, or leave value blank, as appropriate. 2. Expected ranges are provided for lab values. If you have a data point outside of the expected range please record the closest possible value. Example: if WBC = 95.0 x10^cells/uL, record 91.0 3. Please note that the decimal places for the data entered must correspond to the expected range. 4. These can be imported.

First SARS-COV-2 test results

◯ Not done
◯ Negative
◯ Positive
◯ Indeterminate
◯ Unknown
(If patient only had one test and that was on the index visit, then that is the 'First SARS CoV-2 test result' for this question. If the patient had no test on the index visit, but had multiple tests after the index visit, report the 'First SARS CoV-2 test result' from the test done closest in time to the index visit. Then report the next test done in time as the 'Second SARS-CoV-2 test result' and so forth.)

Date of first SARS-CoV-2 test result (if done)

____

Second SARS-COV-2 test results

◯ Not done
◯ Negative
◯ Positive
◯ Indeterminate
◯ Unknown
(If patient only had one test and that was on the index visit, then that is the 'First SARS CoV-2 test result' for this question. If the patient had no test on the index visit, but had multiple tests after the index visit, report the 'First SARS CoV-2 test result' from the test done closest in time to the index visit. Then report the next test done in time as the 'Second SARS-CoV-2 test result' and so forth.)

Date of second SARS-CoV-2 test result (if done)

____

First Influenza A (Type Unknown) test result

◯ Not done
◯ Negative
◯ Positive

First Influenza A (H1) test result

◯ Not done
◯ Negative
◯ Positive

First Influenza A (H3) test result	◯ Not done<br>◯ Negative<br>◯ Positive
First Influenza A (H5) test result	◯ Not done<br>◯ Negative<br>◯ Positive
First Influenza B test result	◯ Not done<br>◯ Negative<br>◯ Positive
Date of first influenza test (if done)	______________________
SECOND Influenza A (Type Unknown) test result	◯ Not done<br>◯ Negative<br>◯ Positive
SECOND Influenza A (H1) test result	◯ Not done<br>◯ Negative<br>◯ Positive
SECOND Influenza A (H3) test result (if done)	◯ Not done<br>◯ Negative<br>◯ Positive
SECOND Influenza A (H5) test result	◯ Not done<br>◯ Negative<br>◯ Positive
SECOND Influenza B test result	◯ Not done<br>◯ Negative<br>◯ Positive
Date of second influenza test (if done)	______________________
Other viral test within 24 hours of SARS-COV-2 testing (e.g., viral panel)?	◯ Yes<br>◯ No<br>(This and the subsequent questions will typically come from a viral panel or other multiplex platform)
Date of first other viral testing	______________________
Adenovirus	◯ Positive<br>◯ Negative<br>◯ Not Done
Other Coronavirus - Type Unknown	◯ Positive<br>◯ Negative<br>◯ Not Done

Other Coronavirus - HKU1	◯ Positive<br>◯ Negative<br>◯ Not Done
Other Coronavirus - NL63	◯ Positive<br>◯ Negative<br>◯ Not Done
Other Coronavirus - 229E	◯ Positive<br>◯ Negative<br>◯ Not Done
Other Coronavirus - OC43	◯ Positive<br>◯ Negative<br>◯ Not Done
Respiratory syncytial virus (RSV) - Type A	◯ Positive<br>◯ Negative<br>◯ Not Done
Respiratory syncytial virus (RSV) - Type B	◯ Positive<br>◯ Negative<br>◯ Not Done
Respiratory syncytial virus (RSV) - Type Unknown	◯ Positive<br>◯ Negative<br>◯ Not Done
Human Metapneumovirus	◯ Positive<br>◯ Negative<br>◯ Not Done
Human Rhinovirus	◯ Positive<br>◯ Negative<br>◯ Not Done
Parainfluenza - Type Unknown	◯ Positive<br>◯ Negative<br>◯ Not Done
Parainfluenza - Virus 1	◯ Positive<br>◯ Negative<br>◯ Not Done
Parainfluenza - Virus 2	◯ Positive<br>◯ Negative<br>◯ Not Done
Parainfluenza - Virus 3	◯ Positive<br>◯ Negative<br>◯ Not Done
Parainfluenza - Virus 4	◯ Positive<br>◯ Negative<br>◯ Not Done

Influenza result from viral panel - Type A	◯ Positive<br>◯ Negative<br>◯ Not Done
Influenza result from viral panel - Type A H1	◯ Positive<br>◯ Negative<br>◯ Not Done
Influenza result from viral panel - Type A H3	◯ Positive<br>◯ Negative<br>◯ Not Done
Influenza result from viral panel - Type B	◯ Positive<br>◯ Negative<br>◯ Not Done
Pertussis - B Parapertussis	◯ Positive<br>◯ Negative<br>◯ Not Done
Pertussis - B Pertussis	◯ Positive<br>◯ Negative<br>◯ Not Done
Other Virus	◯ Positive<br>◯ Negative<br>◯ Not Done
Name Other Virus	__________________________________
Other viral test done within 30 days (e.g., viral panel) (second test)?	◯ Yes<br>◯ No<br>(Only populated if a second test is done within next 30 days. This and the subsequent questions will typically come from a viral panel or other multiplex platform)
Date of SECOND other viral testing	__________________________________
Adenovirus (second test)	◯ Positive<br>◯ Negative<br>◯ Not Done
Other Coronavirus - Type Unknown (second test)	◯ Positive<br>◯ Negative<br>◯ Not Done
Other Coronavirus (second test) - HKU1	◯ Positive<br>◯ Negative<br>◯ Not Done
Other Coronavirus (second test) - NL63	◯ Positive<br>◯ Negative<br>◯ Not Done

Other Coronavirus (second test) - 229E	◯ Positive<br>◯ Negative<br>◯ Not Done
Other Coronavirus (second test) - OC43	◯ Positive<br>◯ Negative<br>◯ Not Done
Respiratory syncytial virus (RSV) (second test) - Type A	◯ Positive<br>◯ Negative<br>◯ Not Done
Respiratory syncytial virus (RSV) (second test) - Type B	◯ Positive<br>◯ Negative<br>◯ Not Done
Respiratory syncytial virus (RSV) (second test) - Type A	◯ Positive<br>◯ Negative<br>◯ Not Done
Human Metapneumovirus (second test)	◯ Positive<br>◯ Negative<br>◯ Not Done
Human Rhinovirus (second test)	◯ Positive<br>◯ Negative<br>◯ Not Done
Parainfluenza (second test) - Type Unknown	◯ Positive<br>◯ Negative<br>◯ Not Done
Parainfluenza (second test) - Virus 1	◯ Positive<br>◯ Negative<br>◯ Not Done
Parainfluenza (second test) - Virus 2	◯ Positive<br>◯ Negative<br>◯ Not Done
Parainfluenza (second test) - Virus 3	◯ Positive<br>◯ Negative<br>◯ Not Done
Parainfluenza (second test) - Virus 4	◯ Positive<br>◯ Negative<br>◯ Not Done
Influenza result from viral panel (second test) - Type A	◯ Positive<br>◯ Negative<br>◯ Not Done
Influenza result from viral panel (second test) - Type A H1	◯ Positive<br>◯ Negative<br>◯ Not Done

Influenza result from viral panel (second test) - Type A H3	◯ Positive<br>◯ Negative<br>◯ Not Done
Influenza result from viral panel (second test) - Type B	◯ Positive<br>◯ Negative<br>◯ Not Done
Pertussis (second test) - Parapertussis B	◯ Positive<br>◯ Negative<br>◯ Not Done
Pertussis (second test) - Pertussis B	◯ Positive<br>◯ Negative<br>◯ Not Done
Chest radiograph done?	◯ Yes<br>◯ No<br>(If patient only had one chest radiograph and that was on the index visit, then that is the radiograph referred to for this question. If the patient had no radiograph on the index visit, but had multiple radiographs after the index visit, report the stauts as 'Yes' from the radiograph done closest in time to the index visit.)
Chest radiograph findings, import conclusion or findings section	______________________________<br>(Use radiologist read. If patient only had one chest radiograph and that was on the index visit, then report the findings for that radiograph for this question. If the patient had no radiograph on the index visit, but had multiple radiographs after the index visit, report the findings from the radiograph done closest in time to the index visit.)
Chest Radiograph Findings	◯ Normal<br>◯ Abnormal<br>◯ Not Done
Chest computed tomography done?	◯ Yes<br>◯ No
Venous ultrasound results (import findings or conclusions)	______________________________<br>(If patient only had one venous ultrasound and that was on the index visit, then report the findings for that venous US for this question. If the patient had no venous US on the index visit, but had multiple venous US after the index visit, report the findings from the venous US done closest in time to the index visit.)

Chest computed tomography findings, import conclusions or findings	______________________ ((Use radiologist read. If patient only had one chest CT and that was on the index visit, then report the findings for that CT for this question. If the patient had no chest CT on the index visit, but had multiple chest CTs after the index visit, report the findings from the CT done closest in time to the index visit.))
Chest computed tomography Findings	◯ Normal<br>◯ Abnormal<br>◯ Not Done
Pregnancy test (urine or serum) positive	◯ Yes<br>◯ No<br>◯ Not Done
WBC (0.0 - 90.0 103/uL)	______________________ (103/uL)
Lymphocyte count (0.0 - 60.0 103 cells/uL)	______________________ (This is a cell count, not a %, report in 103 cells/uL which is the same as 109 cells/L)
Hemoglobin (2.0 - 22.0 g/dL)	______________________ (g/dL)
Platelets (10 - 1M 103 cells/uL)	______________________ (103 cells/uL)
Aspartate transaminase (1 - 5,000 IU)	______________________ (IU)
Alanine transaminase (0 - 5,000 IU)	______________________ (IU)
Total bilirubin (0.0 - 20.0 mg/dL)	______________________ (mg/dL)
Albumin (1.0 - 7.0 g/dL)	______________________ (g/dL)
Alkaline Phosphatase (10 - 1,000 IU)	______________________ (IU)

Na (100 - 180 mEq/L)	______________ (mEq/L)
Cl (70 - 160 mEq/L)	______________ (mEq/L)
K (1.0 -15.0 mEq/L)	______________ (mEq/L)
CO2 (1 - 40 mEq/L)	______________ (mEq/L)
BUN (1 -150 mg/dL)	______________ (mg/dL)
Cr (0.10 - 20.00 mg/dL)	______________ (mg/dL)
Glucose (10 - 2,500 mg/dL)	______________ (mg/dL)
First Documented Troponin	◯ Standard Assay<br>◯ High-sensitivity Assay<br>◯ No troponin recorded
First troponin (0.00 - 100.00 ng/mL)	______________ (ng/mL - if your value has a < in front of it please record the next lowest significant number (i.e. < 0.03 = 0.02))
First High-Sensitivity Troponin (0.0 - 100,000.0 pg/mL)	______________ (pg/mL - if your value has a < in front of it please record the next lowest significant number (i.e. < 0.03 = 0.02))
Troponin Type	◯ I<br>◯ T
d-dimer (200 - 100,000 ng/mL fibrinogen equivalent units)	______________ (If the laboratory result is D-dimer units (typical threshold of abnormal is 200-250 ng/mL), please multiply times two . When the threshold for abnormal is 500 ng/mL for DVT or PE, this always means the machine is reporting FEUs)
C Reactive protein (0.1 - 40.0 mg/dL)	______________ (mg/dL)

Procalcitonin (0.00 - 100.00 mcg/L)

____
(mcg/L)

Outcomes Data Guidelines 1. Day of the index visit from four weeks prior is considered Day 0. 2. All outcomes are up to and including 30 days unless otherwise stated. 3. Outcomes data must be recorded > 30 days from the index visit

Admission definitions:

Index admission refers to an admission that occurs at the Index ED Visit. First admission refers to an admission that occurs at the first ED revisit, if an ED revisit occurred. Second admission, refers to an admission that occurs at the second ED revisit, if a second ED revisit occurred. Third admission, refers to an admission that occurs at the third ED revisit, if a third ED revisit occurred.

If patient admitted at Index, please specify admission location in hospital for the admission.

- ◯ Patient not admitted at Index Visit
- ◯ Initial admit observation
- ◯ Initial admit regular or monitored floor
- ◯ Initial admit stepdown or progressive care
- ◯ Initial admit intensive care unit

Date of Index admission

____

Hospital Length of Stay for Index admission (use -999 if no admission occurred)?

____
(import count from EMR; if still in hospital, count to day of query. If patient is still admitted at 30-day follow up, provide length of stay to that date, and indicate ongoing admission in the subsequent "Index Admission Ongoing" field.)

Index admission ongoing?

- ◯ Yes
- ◯ No

What was the patient's resuscitation/code status at the time of Index admission.

- ◯ Full
- ◯ DNR or (Do not resuscitate) or DNI (Do not intubate) or DNH (Do not hospitalize) or Comfort only
- ◯ Unknown

(Use the latest information in chart)

During Index admission, if admitted to non-ICU floor, was patient transferred to ICU during admission?

- ◯ Yes
- ◯ No

How many return vists for emergency care did the patient have within 30 days after discharge either after the index visit?

____
(import count)

Date of first repeat ED visit

____

Import all ICD codes from first repeat ED visit	__________________________________ (This question may or may not have the same data as the be question on ICD codes in the demographics form)
Was patient admitted on the first return ED visit?	◯ Yes<br>◯ No
If patient admitted at this ED Visit, please specify admission location in hospital for the first admission.	◯ Patient not admitted at this ED visit<br>◯ Patient admitted to observation<br>◯ Patient admitted to regular or monitored floor<br>◯ Patient admitted to stepdown or progressive care<br>◯ Patient admitted to intensive care unit<br>(If patient admitted more than once in 30 days after testing, answer questions for admission closest in time to the SARS-CoV-2 testing used to locate patient)
Date of first admission	__________________________________
Hospital Length of Stay for first admission (use -999 if no admission occurred)?	__________________________________ (import count from EMR; if still in hospital, count to day of query. If patient is still admitted at 30-day follow up, provide length of stay to that date, and indicate ongoing admission in the subsequent "First Admission Ongoing" field.)
First admission ongoing?	◯ Yes<br>◯ No
What was the patient's resuscitation/code status at the time of first admission.	◯ Full<br>◯ DNR or (Do not resuscitate) or DNI (Do not intubate) or DNH (Do not hospitalize) or Comfort only<br>◯ Unknown<br>(Use the latest information in chart)
During first admission, if admitted to non-ICU floor, was patient transferred to ICU during admission?	◯ Yes<br>◯ No
Date of second repeat ED visit	__________________________________
Import all ICD codes from second repeat ED visit	__________________________________ (This question may or may not have the same data as the be question on ICD codes in the demographics form)
Was patient admitted on the second return ED visit?	◯ Yes<br>◯ No

If patient admitted at this ED Visit, please specify admission location in hospital for the second admission.	◯ Patient not admitted at this ED visit<br>◯ Patient admitted to observation<br>◯ Patient admitted to regular or monitored floor<br>◯ Patient admitted to stepdown or progressive care<br>◯ Patient admitted to intensive care unit
Date of second admission	__________________________________
Hospital Length of Stay for second admission (use -999 if no admission occurred)?	__________________________________<br>(import count from EMR; if still in hospital, count to day of query. If patient is still admitted at 30-day follow up, provide length of stay to that date, and indicate ongoing admission in the subsequent "Second Admission Ongoing" field.)
Second admission ongoing?	◯ Yes<br>◯ No
What was the patient's resuscitation/code status at the time of second admission.	◯ Full<br>◯ DNR or (Do not resuscitate) or DNI (Do not intubate) or DNH (Do not hospitalize) or Comfort only<br>◯ Unknown<br>(Use the latest information in chart)
During second admission, if admitted to non-ICU floor, was patient transferred to ICU during admission?	◯ Yes<br>◯ No
Date of third repeat ED visit	__________________________________
Import all ICD codes from third repeat ED visit	__________________________________<br>(This question may or may not have the same data as the be question on ICD codes in the demographics form)
Was patient admitted on the third return ED visit?	◯ Yes<br>◯ No
If patient admitted at this ED Visit, please specify admission location in hospital for the third admission.	◯ Patient not admitted at this ED visit<br>◯ Patient admitted to observation<br>◯ Patient admitted to regular or monitored floor<br>◯ Patient admitted to stepdown or progressive care<br>◯ Patient admitted to intensive care unit
Date of third admission	__________________________________

Hospital Length of Stay for third admission (use -999 if no admission occurred)?	______________________ (import count from EMR; if still in hospital, count to day of query. If patient is still admitted at 30-day follow up, provide length of stay to that date, and indicate ongoing admission in the subsequent "Third Admission Ongoing" field.)
Third admission ongoing?	◯ Yes<br>◯ No
What was the patient's resuscitation/code status at the time of third admission.	◯ Full<br>◯ DNR or (Do not resuscitate) or DNI (Do not intubate) or DNH (Do not hospitalize) or Comfort only<br>◯ Unknown<br>(Use the latest information in chart)
During third admission, if admitted to non-ICU floor, was patient transferred to ICU during admission?	◯ Yes<br>◯ No
Did patient die within 30 days of index ED visit	◯ Yes<br>◯ No
If death, type the number of days from index ED visit	______________________ (Date of triage from index visit)
Import all ICD codes within 30 days (including index ED visit)	______________________ (comma separated)
Import all CPT codes recorded within 30 days (including index ED visit)	______________________ (comma separated)
Import list of all meds in the medication administration record at 30 days	______________________ (comma separated)

30-Day Metadata

Timestamp of Initial 30-Day Follow-up EHR Report	________________
Timestamp of Most Recent 30-Day Follow-up EHR Report	________________
30-Day Follow-Up Created<br>NOTE: SITES DO NOT NEED TO POPULATE THIS FIELD	________________<br>(NOTE: SITES DO NOT NEED TO POPULATE THIS FIELD)
Site<br>NOTE: SITES DO NOT NEED TO POPULATE THIS FIELD	◯ Beaumont Health (William Beaumont Hospital)<br>◯ George Washington University (Medical Faculty Associates)<br>◯ Hennepin County Medical Center<br>◯ Intermountain Medical Center (IHC Health Services, Inc)<br>◯ Massachusetts General Hospital (The General Hospital Corporation)<br>◯ Icahn School of Medicine at Mount Sinai<br>◯ Oregon Health & Science University<br>◯ Penn State Hershey Medical Center<br>◯ Centura Health System Colorado<br>◯ The Ohio State University<br>◯ Trustees of Indiana University<br>◯ University of California San Diego<br>◯ University of Chicago<br>◯ University of Colorado<br>◯ University of Iowa<br>◯ University of Mississippi<br>◯ University of Utah Health Sciences Center<br>◯ University of Wisconsin - Madison-System<br>◯ University Medical Center New Orleans<br>◯ UT Southwestern<br>◯ Washington University in St. Louis<br>◯ Wayne State University<br>◯ West Virginia University<br>(NOTE: SITES DO NOT NEED TO POPULATE THIS FIELD)
DOS Year<br>NOTE: SITES DO NOT NEED TO POPULATE THIS FIELD	◯ 2021<br>◯ 2022<br>◯ 2023<br>◯ 2024<br>◯ 2025<br>(NOTE: SITES DO NOT NEED TO POPULATE THIS FIELD)

DOS Week<br>NOTE: SITES DO NOT NEED TO POPULATE THIS FIELD	◯ 0<br>◯ 1<br>◯ 2<br>◯ 3<br>◯ 4<br>◯ 5<br>◯ 6<br>◯ 7<br>◯ 8<br>◯ 9<br>◯ 10<br>◯ 11<br>◯ 12<br>◯ 13<br>◯ 14<br>◯ 15<br>◯ 16<br>◯ 17<br>◯ 18<br>◯ 19<br>◯ 20<br>◯ 21<br>◯ 22<br>◯ 23<br>◯ 24<br>◯ 25<br>◯ 26<br>◯ 27<br>◯ 28<br>◯ 29<br>◯ 30<br>◯ 31<br>◯ 32<br>◯ 33<br>◯ 34<br>◯ 35<br>◯ 36<br>◯ 37<br>◯ 38<br>◯ 39<br>◯ 40<br>◯ 41<br>◯ 42<br>◯ 43<br>◯ 44<br>◯ 45<br>◯ 46<br>◯ 47<br>◯ 48<br>◯ 49<br>◯ 50<br>◯ 51<br>◯ 52<br>◯ 53<br>(NOTE: SITES DO NOT NEED TO POPULATE THIS FIELD)
EHR Platform<br>NOTE: SITES DO NOT NEED TO POPULATE THIS FIELD	◯ Cerner<br>◯ EPIC<br>(NOTE: SITES DO NOT NEED TO POPULATE THIS FIELD)
Follow-up Source File<br>NOTE: SITES DO NOT NEED TO POPULATE THIS FIELD	______________________________<br>(NOTE: SITES DO NOT NEED TO POPULATE THIS FIELD)

Follow-up Update File
NOTE: SITES DO NOT NEED TO POPULATE THIS FIELD

____
(NOTE: SITES DO NOT NEED TO POPULATE THIS FIELD)
